# Evaluation of Kratom Opioid Derivatives as Potential Treatment Option for Alcohol Use Disorder

**DOI:** 10.3389/fphar.2021.764885

**Published:** 2021-11-03

**Authors:** Anna M. Gutridge, Soumen Chakraborty, Balazs R. Varga, Elizabeth S. Rhoda, Alexander R. French, Arryn T. Blaine, Quinten H. Royer, Haoyue Cui, Jinling Yuan, Robert J. Cassell, Márk Szabó, Susruta Majumdar, Richard M. van Rijn

**Affiliations:** ^1^ Department of Medicinal Chemistry and Molecular Pharmacology, Purdue University, West Lafayette, IN, United States; ^2^ Center for Clinical Pharmacology, University of Heath Sciences and Pharmacy at St. Louis and Washington University School of Medicine, St. Louis, MO, United States; ^3^ Purdue Institute for Integrative Neuroscience, West Lafayette, IN, United States; ^4^ Purdue Institute for Drug Discovery, West Lafayette, IN, United States; ^5^ XiMo Hungary Ltd, Budapest, Hungary

**Keywords:** kratom, alcohol use disorder, nociception, seizures, reward, delta opioid receptor, biased signaling

## Abstract

**Background and Purpose:** *Mitragyna speciosa* extract and kratom alkaloids decrease alcohol consumption in mice at least in part through actions at the δ-opioid receptor (δOR). However, the most potent opioidergic kratom alkaloid, 7-hydroxymitragynine, exhibits rewarding properties and hyperlocomotion presumably due to preferred affinity for the mu opioid receptor (µOR). We hypothesized that opioidergic kratom alkaloids like paynantheine and speciogynine with reduced µOR potency could provide a starting point for developing opioids with an improved therapeutic window to treat alcohol use disorder.

**Experimental Approach:** We characterized paynantheine, speciociliatine, and four novel kratom-derived analogs for their ability to bind and activate δOR, µOR, and κOR. Select opioids were assessed in behavioral assays in male C57BL/6N WT and δOR knockout mice.

**Key Results:** Paynantheine (10 mg∙kg^−1^, i.p.) produced aversion in a limited conditioned place preference (CPP) paradigm but did not produce CPP with additional conditioning sessions. Paynantheine did not produce robust antinociception but did block morphine-induced antinociception and hyperlocomotion. Yet, at 10 and 30 mg∙kg^−1^ doses (i.p.), paynantheine did not counteract morphine CPP. 7-hydroxypaynantheine and 7-hydroxyspeciogynine displayed potency at δOR but limited µOR potency relative to 7-hydroxymitragynine *in vitro*, and dose-dependently decreased voluntary alcohol consumption in WT but not δOR in KO mice. 7-hydroxyspeciogynine has a maximally tolerated dose of at least 10 mg∙kg^−1^ (s.c.) at which it did not produce significant CPP neither alter general locomotion nor induce noticeable seizures.

**Conclusion and Implications:** Derivatizing kratom alkaloids with the goal of enhancing δOR potency and reducing off-target effects could provide a pathway to develop novel lead compounds to treat alcohol use disorder with an improved therapeutic window.

## Introduction

*Mitragyna speciosa*, commonly known as kratom, is growing increasingly popular in the United States, with nearly 1% of the population aged 12 and older using kratom in 2019 (Palamar, 2021). While kratom is most commonly used to self-manage pain or reduce dependence to opioids and opiates (Coe et al., 2019), a recent online survey revealed that 18% of kratom users indicate reducing or quitting alcohol consumption as a reason they use kratom (Coe et al., 2019). This indication is in line with reports of individuals claiming that kratom was useful for reducing their alcohol intake (Havemann-Reinecke, 2011; Singh et al., 2014; Suhaimi et al., 2021). We have previously demonstrated that systemic injections of the kratom extract and kratom alkaloids (7-hydroxymitragynine, paynantheine, speciogynine, and mitragynine) decrease voluntary alcohol drinking in mouse models of moderate and binge alcohol consumption, with the kratom alkaloid 7-hydroxymitragynine being the most efficacious (Gutridge et al., 2020). Kratom alkaloids differ from opium-derived opioids and clinically used synthetic opioids in that upon binding to opioid receptors they activate the Gα_i/o_ protein, without promoting β-arrestin recruitment to the receptor (Kruegel et al., 2016; Váradi et al., 2016; Faouzi et al., 2020; Chakraborty and Majumdar, 2021). Several preclinical studies in mice strongly suggest that β-arrestin recruitment at the delta opioid receptor (δOR) is a liability for enhanced alcohol use and should be avoided (Chiang et al., 2016; Robins et al., 2018; Gutridge et al., 2020). We have previously demonstrated that 7-hydroxymitragynine and other kratom alkaloids poorly recruit β-arrestin-2 at mu opioid receptors (µORs) and δORs and possess a degree of G-protein bias at this receptor (Gutridge et al., 2020). Moreover, our studies in δOR knockout mice revealed that 7-hydroxymitragynine’s modulation of alcohol consumption was due to its activity at the δOR (Gutridge et al., 2020).

However, a possible concern is that 7-hydroxymitragynine and other kratom alkaloids generally have comparable, if not higher, affinity and potency at the µOR (Takayama et al., 2002; Matsumoto et al., 2004).While this µOR potency may be responsible for the alkaloids’ ability to promote antinociception in mice (Matsumoto et al., 2004; Obeng et al., 2020; Wilson et al., 2020, 2021) and in humans (Vicknasingam et al., 2020), it appears that because of their µOR potency, kratom alkaloids, especially 7-hydroxymitragynine, are shown or predicted to share some of the same negative side effects associated with traditional opioids such as abuse liability. Accordingly, in rodent preclinical studies, 7-hydroxymitragynine has been shown to have rewarding qualities in models of conditioned place preference and self-administration, which indicates that it may have abuse liability (Yue et al., 2018; Hemby et al., 2019; Gutridge et al., 2020). Likewise, withdrawal symptoms following kratom exposure have also been recorded in rodents (Matsumoto et al., 2005; Wilson et al., 2021). Similarly, regular kratom use in humans leads to dependence problems in over 50% of users (Singh et al., 2014), and kratom withdrawal symptoms equally have been widely reported in humans (Singh et al., 2014; Saref et al., 2019; Stanciu et al., 2019; Anand and Hosanagar, 2021). Likely attributed to its potency at the µOR, another side effect of 7-hydroxymitragynine in mice is hyperlocomotion (Becker et al., 2000; Gutridge et al., 2020); this effect mirrors one of kratom’s traditional uses as a stimulant (Suwanlert, 1975; Ahmad and Aziz, 2012). Still, relative to traditional opioids such as morphine, the negative side effect profile of kratom and kratom opioids is slightly lessened in regards to reward, respiratory depression, and withdrawal symptoms (Hemby et al., 2019; Wilson et al., 2020, 2021). This reduction in side effect profile was first attributed to G-protein–biased activity of the kratom alkaloids at the µOR (Kruegel et al., 2016; Váradi et al., 2016), but new research suggests that partial agonism at the µOR likely drives these effects (Gillis et al., 2020; Bhowmik et al., 2021; Uprety et al., 2021). Despite the reduced µOR-mediated side effects relative to traditional opioids, kratom use is not without risk, and this is reflected in controversial efforts to place 7-hydroxymitragynine and mitragynine under Schedule I regulation by the Drug Enforcement Agency (DEA, 2016; Griffin and Webb, 2018).

An additional side effect of kratom use is seizure activity (Coonan and Tatum, 2021). In rats, abnormal EEG activity has been reported following chronic exposure to mitragynine, the most abundant alkaloid in kratom (Suhaimi et al., 2021). In humans, several individual case reports have highlighted seizure side effects induced by kratom use or withdrawal (Boyer et al., 2008; Nelsen et al., 2010; Tatum et al., 2018; Burke et al., 2019; Afzal et al., 2020; Valenti et al., 2021), and a retrospective analysis of kratom exposure reports in the National Poison Data System reveals that 6.1% of reports detail seizure side effects (Eggleston et al., 2019). Currently, the mechanism underlying these reported seizure effects of kratom have not been defined.

We hypothesized that compared to 7-hydroxymitragynine, derivatizing kratom analogs with reduced µOR potency relative to δOR potency would reduce restrictive side effects such as abuse liability and hyperlocomotion, leading to an increased therapeutic window. Prior efforts have been made to utilize unique kratom alkaloid scaffolds to develop improved therapeutic options (Kruegel et al., 2016; Chakraborty et al., 2021a; Wilson et al., 2021). Similarly, here we investigate four novel kratom-derived analogs as well as two naturally occurring kratom alkaloids for their ability to decrease alcohol consumption, while monitoring lead compounds for their ability to produce seizure activity, induce reward properties, and affect general locomotion.

## Tail Flick Thermal Nociception Assay

### Materials

Kratom “Red Indonesian Micro Powder” was purchased from Moon Kratom (Austin, TX, United States). Corynoxine and corynoxine B were purchased from BOC Sciences (NY, United States). Leu-enkephalin, forskolin, and morphine sulfate pentahydrate were purchased from Sigma-Aldrich (St. Louis, MO, United States). (2S)-2-[[2-[[(2R)-2-[[(2S)-2-Amino-3-(4-hydroxyphenyl)propanoyl] amino] propanoyl] amino]acetyl]-methylamino]-N-(2-hydroxyethyl)-3-phenylpropanamide (DAMGO), 2-(3,4-dichlorophenyl)-N-methyl-N-[(1R,2R)-2-pyrrolidin-1-ylcyclohexyl]acetamide (U50,488), and naloxone hydrochloride were purchased from Tocris Bioscience (Bio-Techne Corporation, Minneapolis, MN, United States). [3H]DAMGO (53.7 Ci/mmol, lot#2376538; 51.7 Ci/mmol, lot#2815607), [3H]U69,593 (60 Ci/mmol, lot#2367921 and lot#2644168; 49.2 Ci/mmol, lot#2791786), and [3H]DPDPE (49.2 CI/mmol, lot#2573313 and lot#2726659; 48.6 Ci/mmol, lot#2826289) were purchased from PerkinElmer (Waltham, MA, United States). For *in vivo* experiments, morphine and naloxone were prepared in a saline vehicle. Kratom-derived analogs were dissolved in a 1:1:8 ethanol:cremophor:saline vehicle for all behavioral experiments. For the two-bottle choice experiment in δOR KO mice, paynantheine was prepared in the same 1:1:8 ethanol:cremophor:saline vehicle. For all other experiments paynantheine and speciociliatine were dissolved in a slightly acidic saline solution that was adjusted to a pH of 6–7 before administration.

### Chemistry

#### General

All chemicals were purchased from Sigma-Aldrich Chemicals and used without further purification. Reactions were carried out in flame-dried reaction flasks under Argon. Reaction mixtures were purified by silica flash chromatography on E. Merck 230–400 mesh silica gel 60 using a Teledyne ISCO CombiFlash Rf instrument with UV detection at 280 and 254 nm. RediSep Rf silica gel normal phase columns were used. The yields reported are isolated yields. NMR spectra were recorded on a Varian 400/500 MHz NMR spectrometer. NMR spectra were processed with MestReNova software. The chemical shifts were reported as δ ppm relative to TMS using residual solvent peak as the reference unless otherwise noted (CDCl_3_^1^H: 7.26, ^13^C: 77.3). Peak multiplicity is reported as follows: s, singlet; d, doublet; t, triplet; q, quartet; and m, multiplet. Coupling constants (J) are expressed in Hz. High resolution mass spectra were obtained on a Bruker Daltonics 10 Tesla Apex Qe Fourier-Transform Ion Cyclotron Resonance–Mass Spectrometer by electrospray ionization (ESI). Accurate masses are reported for the molecular ion [M + Na]^+^.

#### Isolation of Mitragynine From *Mitragyna speciosa* (Kratom)

Mitragynine was extracted from the powdered leaves by following our previously reported methods (Gutridge et al., 2020). Kratom powder (500 g) was heated to reflux in MeOH 700 ml for 40 min. The suspension was filtered and the methanolic extraction process was repeated (3 × 500 ml). The solvent of the combined methanolic extract was removed under reduced pressure and the content was dried using high vacuum. The dry residue was resuspended in 20% acetic acid solution (1 L) and washed with petroleum ether (4 × 500 ml). The aqueous layer was then cooled on ice bath and basified (pH ∼9) with aqueous NaOH solution (3.5 M. ∼1 L) slowly. Alkaloids were extracted in DCM (4 × 400 ml) from the aqueous layer. The combined DCM layer was washed with brine 300 ml, dried over anhydrous Na_2_SO_4_, and filtered. The solvent was removed under reduced pressure, and the residue was dried under high vacuum to obtain the kratom extract (9.8 g). Then this crude kratom extract was subjected to silica gel column chromatography, using 0–15% MeOH in dichloromethane to isolate mitragynine (4.7 g), paynantheine (568 mg), speciogynine (343 mg), and speciociliatine (754 mg), along with some minor alkaloids.

#### 7-Hydroxypaynantheine (7OH Pay/7)

Paynantheine (100 mg, 0.25 mmol) was dissolved in acetonitrile (7 ml), and then water (2 ml) was added. The resulting suspension was cooled to 0°C, and PIFA (108 mg, 1.1 equiv) dissolved in acetonitrile (1.1 ml) was added slowly over the course of several minutes. The reaction mixture was stirred at 0°C for 45 min. Then saturated aqueous NaHCO_3_ solution was added, and the mixture extracted with EtOAc (3 × 15 ml). The organic phase was washed with brine (20 ml) and dried over anhydrous Na_2_SO_4_. The solvent was removed under reduced pressure. The residue was purified on a silica column using 10–75% EtOAc in hexanes as eluent. The fractions containing the product were evaporated to yield 42 mg (40%) of **9** as a light magenta amorphous powder. ^1^H δ (400 MHz, ppm): 7.31 (1H, s, 17); 7.29 (1H, t, 3J = 7.7 Hz, 11); 7.19 (1H, t, 3J = 7.7 Hz, 12); 6.74 (1H, d, ^3^ *J* = 7.7 Hz, 10); 5.57 (1H, ddd, ^3^ *J* = 18.0, 10.3, 7.2 Hz, 19); 4.99 (1H, dd, ^3^ *J* = 18.0, ^2^ *J* = 1.5 Hz, 18 *trans*); 4.94 (1H, dd, ^3^ *J* = 10.3, ^2^ *J* = 1.5 Hz, 18 *cis*); 3.86 (3H, s, 9-OMe); 3.79 (3H, s, 17-OMe); 3.68 (3H, s, 16-COOMe); 3.46 (1H, s, 7-OH); 3.23 (1H, m, 3); 3.03 (1H, m, 21/1); 3.01 (1H, m, 20); 2.85 (1H, m, 5/2); 2.73 (1H, m, 5/1); 2.72 (1H, m, 15); 2.66 (1H, m, 6/1); 2.39 (1H, m, 14/1); 2.30 (1H, m, 21/2); 2.05 (1H, m, 14/2); 1.70 (1H, m, 6/2). ^13^C δ (100 MHz, ppm): 183.5 (2); 168.8 (16-CO); 159.8 (17); 155.9 (9); 154.9 (13); 139.3 (19); 131.0 (11); 126.4 (8); 115.4 (18); 114.3 (12); 111.4 (16); 109.1 (10); 81.0 (7); 61.6 (21); 61.5 (17-OMe); 60.2 (3); 55.5 (9-OMe); 51.2 (16-COOMe); 49.8 (5); 42.8 (20); 38.2 (15); 35.9 (6); 30.4 (14). Relative configuration was determined based on the NOE cross peaks between the following 1H nuclei: 3 – 5/2; 3 – 14/2; 3 – 21/2; 3 – 5/2; 15 – 19; 19 – 21/2 (/1 always indicates the hydrogen pointing towards the reader from the paper; /2 indicate the hydrogen pointing behind the plain of the paper). HRMS (ESI-TOF) m/z: [M+Na]^+^ Calcd for C_23_H_28_N_2_NaO_5_ 435.189043; found. 435.189116.

#### Paynantheine Pseudoindoxyl (Pay PI/8)

7-Hydroxypaynantheine (**9**, 40 mg, 0.1 mmol) was dissolved in dry toluene (1.5 ml), and Zn(OTf)_2_ (70 mg, 2 equiv) was added. The reaction mixture was stirred in a sealed tube for 30 min at 115°C. To the cooled mixture were added 2 ml sat. aqueous NaHCO_3_ solution and water (5 ml) and the organics were extracted with EtOAc (10 ml). The organic layer was rinsed with brine (10 ml) and dried over anhydrous Na_2_SO_4_. After evaporation of the solvent under reduced pressure, the residue was purified by flash column chromatography on silica (gradient: 40–75% EtOAc in hexanes) to yield 15 mg (38%) of product as a light yellow gum. ^1^H δ (400 MHz, ppm): 7.32 (1H, t, ^3^ *J* = 8.2 Hz, 11); 7.18 (1H, s, 16); 6.37 (1H, d, ^3^ *J* = 8.2 Hz, 12); 6.13 (1H, d, ^3^J = 8.2 Hz, 10); 5.49 (1H, ddd, ^3^ *J* = 18.2, 10.3, 7.4 Hz, 19); 5.25 (1H, br s, 1); 4.95 (1H, d, ^3^ *J* = 18.2, 18 *trans*); 4.9 (1H, d, ^3^ *J* = 10.3, 18 *cis*); 3.89 (3H, s, 9-OCH3); 3.73 (3H, s, 17-OCH3); 3.62 (3H, s, 16-COOCH_3_); 3.23 (1H, m, 5/1); 3.11 (1H, m, 21/1); 2.87 (1H, m, 20); 2.49 (1H, m, 15); 2.39 (1H, m, 5/2); 2.39 (1H, m, 6/2); 2.34 (1H, m, 3); 1.98 (1H, m, 21/2); 1.94 (1H, m, 6/1); 1.79 (1H, br q ^3^ *J* = 11.3 Hz, 14/1); 1.26 (1H, br d, ^3^ *J* = 11.3 Hz, 14/2). ^13^C δ (100 MHz, ppm): 199.8 (7); 168.2 (16-C=O); 162.1 (13); 159.7 (17); 158.7 (9); 139.5 (19); 139 (11); 115.6 (18); 111.9 (16); 109.5 (8); 104 (12); 99.2 (10); 74.7 (2); 72.4 (3); 61.5 (17-O-CH_3_); 58.8 (21); 55.8 (9-OCH_3_); 53.2 (5); 51.1 (COO-CH_3_); 42.3 (20); 36.9 (15); 35.3 (6); 28.3 (14). Relative configuration was determined based on the NOE cross peaks between the following ^1^H nuclei: 1 – 6/1; 3 – 14/2; 1 – 14/1; 14/1 – 20; 15 – 19; 19 – 21/2. HRMS (ESI-TOF) m/z: [M+Na]^+^ Calcd for C_23_H_28_N_2_NaO_5_ 435.189043; found. 435.189219.

#### 7-Hydroxyspeciogynine (7OH Spg/9)

Speciogynine (200 mg, 0.5 mmol) was dissolved in acetonitrile (15 ml), and then water (5 ml) was added. The resulting suspension was cooled to 0°C, and PIFA (216 mg, 1.1 equiv) dissolved in acetonitrile (2.2 ml) was added slowly over the course of several minutes. The reaction mixture was stirred at 0°C for 1 h. Then saturated aqueous NaHCO_3_ solution was added, and the mixture extracted with EtOAc (3 × 40 ml). The organic phase was washed with brine (30 ml) and dried over anhydrous Na_2_SO_4_. The solvent was removed under reduced pressure. The residue was redissolved in DCM and was purified using silica column chromatography 10–75% EtOAc in hexanes. The fractions containing the product were evaporated to yield 107 mg (57%) of **9** as a light brown amorphous powder. ^1^H NMR (400 MHz, Chloroform-d) δ 7.36–7.29 (m, 1H), 7.26 (dd, J = 8.8, 7.2 Hz, 1H), 7.17 (d, J = 7.7 Hz, 1H), 6.71 (d, J = 8.3 Hz, 1H), 3.84 (s, 3H), 3.75 (s, 3H), 3.66 (s, 3H), 3.21 – 3.08 (m, 2H), 2.82 (t, J = 12.3 Hz, 1H), 2.77–2.69 (m, 1H), 2.64 (d, J = 14.4 Hz, 1H), 2.54 (t, J = 11.2 Hz, 1H), 2.30 (d, J = 11.9 Hz, 1H), 2.17 (t, J = 10.5 Hz, 1H), 2.06 (t, J = 11.2 Hz, 2H), 1.80 (s, 1H), 1.69 (td, J = 13.5, 4.5 Hz, 1H), 1.40 (s, 1H), 1.02 (d, J = 17.1 Hz, 1H), 0.82 (t, J = 7.4 Hz, 3H). ^13^C NMR (100 MHz, Chloroform-d) δ 183.9, 169.61, 160.10, 156.07, 155.15, 131.15, 126.52, 114.42, 111.44, 109.18, 81.16, 61.98, 61.49, 61.52, 55.66, 51.64, 50.21, 39.54, 38.87, 36.13, 24.49, 11.56, and 11.29. HRMS (ESI-TOF) m/z: [M+Na]^+^ Calcd for C_23_H_30_N_2_NaO_5_ 437.204693; found. 437.204951.

#### Speciogynine Pseudoindoxyl (Spg PI/10)

7-hydroxyspeciogynine (**9**, 200 mg, 0.48 mmol) was dissolved in dry toluene (6 ml), and Zn(OTf)_2_ (350 mg, two equivalent) was added. The reaction was stirred in a sealed tube for 2 h at 100°C. To the cooled mixture were added 10 ml sat. aqueous NaHCO_3_ solution and water (20 ml), and extracted with EtOAc (30 ml). The organic layer was rinsed with brine (20 ml) and dried over anhydrous Na_2_SO_4_. After evaporation of the solvent under reduced pressure, the residue was redissolved in DCM and purified by flash column chromatography (gradient: 40–75% EtOAc in hexanes) to yield 78 mg (39%) of 10 as a light yellow amorphous powder. ^1^H NMR (500 MHz, Chloroform-d) 7.31 (1H, t, ^3^ *J* = 8.2 Hz, 11), 7.23 (1H, s, 17), 6.36 (1H, d, ^3^ *J* = 8.2 Hz, 12), 6.12 (1H, d, ^3^ *J* = 8.2 Hz, 10), 5.34 (1H, br s, 1), 3.89 (3H, s, 9-OMe), 3.72 (3H, s, 17-OMe), 3.62 (3H, s, 16-COOMe), 3.25 – 3.23 (1H, m, 21/1), 3.22 – 3.21 (1H, m, 5/1), 2.37 – 2.35 (2H, m, 5/2; 6/2), 2.33 – 2.31 (1H, m, 15), 2.29 – 2.28 (1H, m, 3), 2.08 – 2.04 (1H, m, 20), 1.94 – 1.90 (1H, m, 6/1), 1.81 – 1.77 (1H, m, 14/1), 1.75 – 1.73 (1H, m, 21/2), 1.34 –1.30 (1H, br m, 19/1), 1.18–1.15 (1H, m, 14/2), 0.95–0.92 (1H, br m, 19/2), and 0.79 (3H, br, 18). ^13^C NMR (100 MHz, Chloroform-d) 200.18 (7), 168.02 (16-CO), 162.25 (13), 160.27 (17), 158.83, (9), 139.17 (11), 112.22 (16), 109.5 (8), 104.26 (12), 99.17 (10), 74.94 (2), 72.94 (3), 61.51 (17-OMe), 58.42 (21), 55.99 (9-OMe), 53.57 (5), 51.07 (16-COOMe), 38.15 (20), 37.50 (15), 35.48 (6), 28.95 (4), 24.46 (9), and 11.35 (18). Relative configuration was determined based on the NOE cross peaks between the following ^1^H nuclei: 1 – 6/1; 1 – 14/1; 15 – 19; 19 – 21/2 (/1 always indicates the hydrogen pointing towards the reader from the paper; /2 indicate the hydrogen pointing behind the plain of the paper). HRMS (ESI-TOF) m/z: [M+Na]^+^ Calcd for C_23_H_30_N_2_NaO_5_ 437.204693; found. 437.204760.

### Cellular Assays and Associated Statistical Analysis

#### Membrane Isolation and Competitive Radioligand Binding Assay

Membrane isolation and subsequent binding assays were completed as described previously using membranes stably expressing the μOR, δOR, or κOR were isolated from CHO (μOR, δOR) or U2OS cells (κOR) (DiscoverX), and using OR specific radiolabels [^3^H]DAMGO, [^3^H]DPDPE, and [^3^H]U69,593 (Cassell et al., 2019; Creed et al., 2021).

#### GloSensor cAMP Inhibition Assay

cAMP inhibition assays were performed in HEK cells and transiently transfected with pGloSensor22F, and either expressing FLAG-mouse δOR, HA-mouse µOR, or FLAG-mouse κOR, as previously described (Chiang et al., 2016).

#### PathHunter β-Arrestin-2 Recruitment Assay

β-Arrestin recruitment assays were performed in PathHunter cells stably expressing the μOR, δOR, or κOR and β-arrestin-2, as previously described (Chiang et al., 2016).

#### Statistical Analysis

Data and statistical analysis comply with the recommendations on experimental design and analysis in pharmacology (Curtis et al., 2018). The data analysis was completed using GraphPad 9 (GraphPad Prism software, La Jolla, CA, United States) and is presented as mean ± SEM. For findings from cellular assays, composite figures are shown consisting of an averaged curve from a minimum of three independent assays that were normalized to a positive control; best fit values in Table 1 were generated by GraphPad Prism from composite figures.

**TABLE 1:** Pharmacological characterization of kratom derivatives at the µ, δ, and κ opioid receptors.

Compounds	Binding		cAMP			β-arrestin-2	
µOR	pK_i_	K_i_ (µM)	pIC_50_	IC_50_ (µM)	α	pEC_50_	α
DAMGO	9.6 ± 0.1 (1)	0.00024	8.0 ± 0.1 (6)	0.0099	100	6.6 ± 0.1 (6)	100
SPECIO	7.1 ± 0.1 (3)	0.086	6.4 ± 0.2 (5)	0.43	38 ± 3	ND (4)	ND
SPG PI	7.1 ± 0.1 (3)	0.077	6.6 ± 0.2 (5)	0.23	58 ± 4	ND (4)	ND
7OH SPG	7.7 ± 0.1 (3)	0.021	6.2 ± 0.2 (6)	0.61	66 ± 6	ND (4)	ND
7OH PAYN	5.2 ± 0.1 (3)	6.15	4.7 ± 0.5 (5)	21.8	80 ± 40	ND (3)	ND
PAYN PI	6.2 ± 0.1 (3)	0.68	5.3 ± 0.2 (4)	4.82	60 ± 6	ND (3)	ND
δOR	pK_i_	K_i_ (µM)	pIC_50_	IC_50_ (µM)	α	pEC_50_	α
Leu-Enk	9.2 ± 0.1 (3)	0.00070	8.4 ± 0.1 (9)	0.0042	100	7.4 ± 0.1 (7)	100
SPECIO	5.4 ± 0.1 (3)	4.34	ND (3)	ND	ND	ND (5)	ND
SPG PI	6.0 ± 0.1 (3)	0.94	5.1 ± 0.3 (4)	8.53	80 ± 20	ND (4)	ND
7OH SPG	6.3 ± 0.1 (3)	0.46	5.6 ± 0.1 (6)	2.27	76 ± 6	ND (4)	ND
7OH PAYN	4.9 ± 0.2 (4)	12.7	5.2 ± 0.3 (5)	5.74	70 ± 20	ND (3)	ND
PAYN PI	6.0 ± 0.1 (3)	0.92	ND (5)	ND	ND	ND (3)	ND
κOR	pK_i_	K_i_ (µM)	pIC_50_	IC_50_ (µM)	α	pEC_50_	α
U50,488	10.0 ± 0.2 (2)	0.000099	8.5 ± 0.1 (5)	0.0034	100	7.1 ± 0.1 (6)	100
SPECIO	6.2 ± 0.1 (4)	0.59	5.6 ± 0.2 (4)	2.50	60 ± 7	ND (5)	ND
SPG PI	6.1 ± 0.1 (3)	0.75	4.7 ± 0.5 (4)	20.6	80 ± 30	ND (3)	ND
7OH SPG	5.8 ± 0.2 (3)	1.63	5.1 ± 0.3 (3)	7.71	80 ± 20	ND (5)	ND
7OH PAYN	5.1 ± 0.1 (3)	7.46	ND (3)	ND	ND	ND (3)	ND
PAYN PI	5.9 ± 0.1 (4)	1.31	ND (3)	ND	ND	ND (3)	ND

Affinity (pKi, drug concentration at which 50% of receptors is occupied). cAMP inhibition potencies (pIC_50,_ drug concentration at 50% maximal efficacy) and efficacies (α, % inhibition at maximal efficacy normalized to DAMGO [µOR], Leu-enkephalin [δOR], or U50,488 [κOR]) of OR agonists to inhibit cAMP production are indicated ± SEM. β-arrestin-2 recruitment potencies (pEC_50_) and efficacies (α, normalized to DAMGO, Leu-enkephalin or U50,488) of OR agonists to recruit β-arrestin 2 are indicated ± SEM. The number of repetitions for each drug is indicated in parentheses. ND, not detectable. Data for 7-hydroxymitragynine, speciogynine, and paynantheine in the GloSensor cAMP assay and PathHunter β-arrestin-2 recruitment assay was generated in a previous publication (Gutridge et al., 2020) and is shown in Supplemental Table S1 for easy comparison to the kratom derivatives.

### Animals

#### General

The animal protocols (#1305000864 and #1605001408) describing the care and use of experimental animals was approved by the Purdue University Institutional Animal Care and Use Committee (https://www.purdue.edu/research/regulatory-affairs/animal-research/staff.php). Animal studies were carried out in accordance with the ARRIVE guidelines (Kilkenny et al., 2010) and recommendations made by the National Institutes of Health Guide for the Care and Use of Laboratory Animals. Wild-type C57Bl/6N mice (107 male, 10 female; 6–7 weeks old) were purchased from Envigo (Indianapolis, IN, United States) and were acclimated to the facility and to handling and injections for 1 week prior to any experimental procedures. δOR KO mice (27 male, 8–12 weeks old) with a C57Bl/6N background (re-derived in early 2021) were bred in-house and were similarly conditioned to handling and injections prior to experimentation. All mice were housed on a reverse 12-h light (21:30–9:30)/12-h dark cycle under controlled temperature (21–23°C) with *ad libitum* food access. The only exception to this is mice used in the rotarod assay; these mice were housed in 12-h light (6:00–18:00)/12-h dark cycle. All experiments were conducted between 10:30 and 15:00, and all mice were habituated to the test room at least 30 min prior to experimentation. Rotarod, nociception, and seizure experiments were conducted in well-lit rooms, whereas conditioned place preference, two-bottle choice, and locomotor experiments were conducted in the dark.

#### Experimental Groups

For the locomotor assays with 7-hydroxymitragynine, a group of 10 male mice was used. For the paynantheine agonist nociception assays, 10 male mice were treated on different days with 10 and 30 mg∙kg^−1^ (i.p.) paynantheine. For the paynantheine antagonist nociception assays, a separate group of 10 mice were exposed to 6 mg∙kg^−1^ morphine (s.c.) by itself, and then again after treatment with 10 and 30 mg∙kg^−1^ paynantheine (i.p.). For agonist and antagonist antinociception assays with 7-hydroxyspeciogynine, a total of 11 wild-type male mice were used; all received 7-hydroxyspeciogynine for the agonist mode, and then for antagonist mode, *n* = 6 received morphine plus 7-hydroxyspeciogynine and *n* = 5 received vehicle plus 7-hydroxyspeciogynine. For specifics on drug administration timing in the nociception assays, see the Methods section titled Tail Flick Thermal Nociception Assay. For the two-bottle choice alcohol consumption experiments with WT male and female mice, separate groups of wild-type mice were used to test increasing doses of each analog (*n* = 8 males for 7-hydroxypaynantheine, *n* = 12 males and *n* = 10 females for 7-hydroxyspeciogynine). For the two-bottle choice experiments with δOR KO mice, a group of mice (*n* = 9) was repeatedly tested once per week with different drug treatments (consistent baseline ethanol consumption across the drug treatments is shown in Supplementary Figure S5). A second separate group of 10 male δOR KO mice was used to examine speciociliatine in the two-bottle choice paradigm. Following a 3-week period of alcohol withdrawal, five of the δOR KO mice from the first two-bottle choice group were used to examine seizure activity of paynantheine (30 mg∙kg^−1^, i.p.). Similarly, five wild-type mice from the naloxone-block locomotor experiment were reused to assess seizure activity of 30 mg∙kg^−1^ paynantheine (i.p.) following a week of drug washout. In the rotarod assay, *n* = 8 wild-type male and *n* = 8 δOR KO male mice were used to assess motor incoordination effects following treatment with speciociliatine. Note that one δOR KO mouse died after experiencing severe level 5–6 seizures following i.p. administration of 30 mg/kg speciociliatine in the rotarod assay, leading to an overall *n* = 7 instead of *n* = 8 for this genotype. For the CPP paradigms, independent groups of wild-type male mice were used to examine paynantheine by itself (*n* = 16 total), paynantheine with morphine (*n* = 14 total), and 7-hydroxyspeciogynine (*n* = 8).

### Behavioral Assays and Associated Statistical Analysis

#### Locomotor Evaluation

**FIGURE 1:**
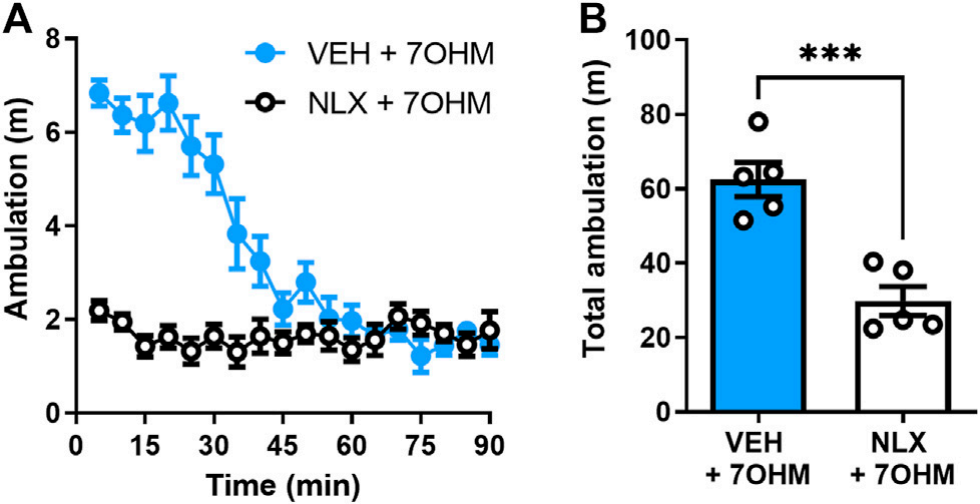
Blocking μOR attenuates 7-hydroxymitragynine (7OHM) induced hyperlocomotion. **(A)** 90-min ambulation time course of wild-type, C57Bl/6 male mice (*n* = 5 per group) treated with 7-hydroxymitragynine (3 mg∙kg^−1^, i.p.) after pretreatment with the vehicle (s.c.) or naloxone (1 mg∙kg^−1^, s.c., NLX) injection (10 min prior to 7-hydroxymitragynine injection). **(B)** Total ambulation (area under the curve) for the same data set. ***p < 0.001 (for details, see Supplementary Table S2).

**FIGURE 2:**
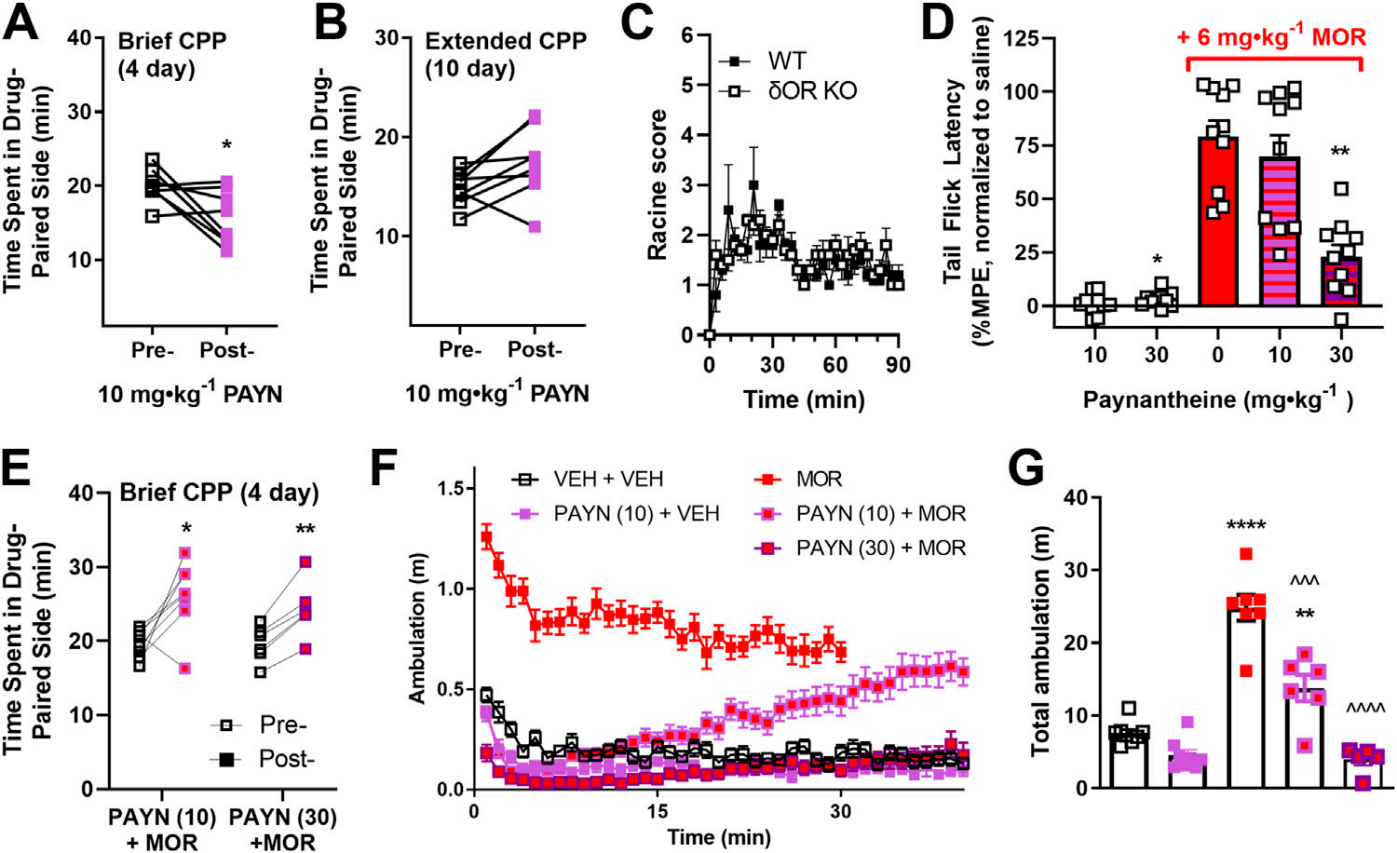
Antagonistic action of paynantheine *in vivo*. The agonistic and antagonistic actions of kratom alkaloid paynantheine were further investigated in C57Bl/6 mice. Paynantheine (10 mg∙kg^−1^, i.p. PAYN) was evaluated in a **(A)** 4-day and **(B)** 10-day model of conditioned place preference (CPP, two vs. four drug conditioning sessions, respectively, *n* = 8 each). **(C)** Seizure activity induced by paynantheine (30 mg∙kg^−1^, i.p.) was evaluated in male δOR KO and WT mice (*n* = 5 per group). **(D)** Paynantheine (10 and 30 mg∙kg^−1^, i.p.) was tested for agonist and antagonistic properties in male mice (*n* = 10 per dose) *via* the tail flick thermal nociception assay. For the antagonist assays, morphine (6 mg∙kg^−1^, s.c., MOR) was administered 10 min following a dose of paynantheine (10 or 30 mg∙kg^−1^, i.p.). Nociception data are expressed as maximum possible effect (%MPE) normalized to a saline baseline (treatment–saline baseline). **(E)** Paynantheine (10 and 30 mg∙kg^−1^, i.p.) was evaluated for agonist and antagonist activity in an acute model of conditioned place preference by administering 10 min prior to morphine (6 mg∙kg^−1^) or the vehicle (*n* = 8 for 10 mg∙kg^−1^ doses, *n* = 6 for 30 mg∙kg^−1^ dose). Locomotor data were extracted from the conditioning sessions of the CPP experiments in **(A**,**E)** and is shown as **(F)** ambulation over time and **(G)** total ambulation (total area under curve). For comparison in **(F**,**G)**, locomotor data for morphine (6 mg∙kg^−1^ morphine) was extracted from a previous CPP experiment with 30-min conditioning sessions. The vehicle locomotor data were extracted from the non–drug-paired side conditioning session for the 10 mg∙kg^−1^ paynantheine + vehicle group. For locomotor data in **(G)**, statistical significance of drug treatment vs. vehicle (VEH + VEH) is shown with stars; statistical significance between paynantheine + morphine treatments and morphine-only treatment (MOR) is shown with carets. *p < 0.05, **p < 0.01, ^^^*p* < 0.001, *** or ^^^^*p* < 0.0001 (for details, see Supplemental Tables S2–S5).

**FIGURE 3:**
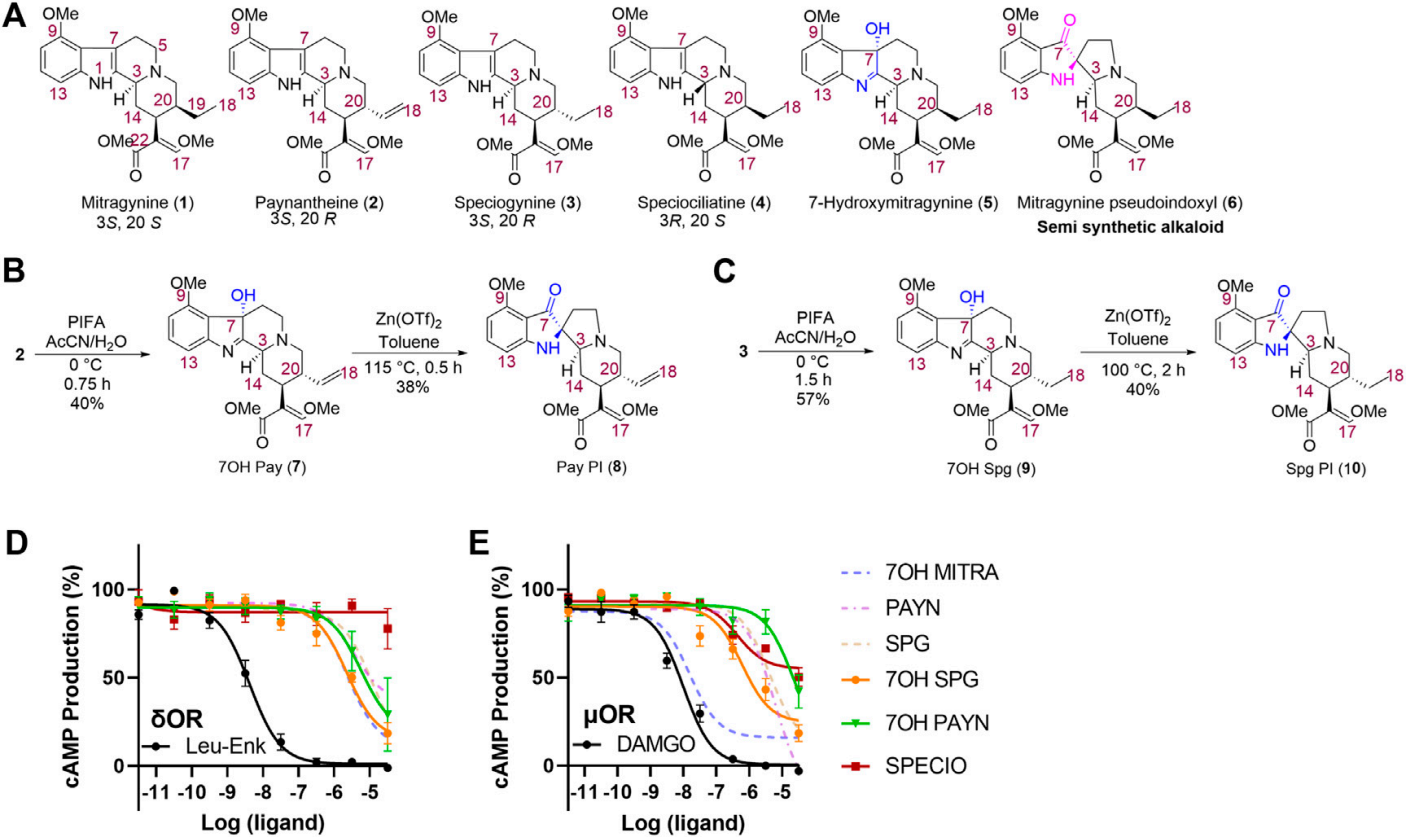
Synthesis and characterization of kratom alkaloid analogs. Structures of naturally occurring kratom alkaloids paynantheine and speciogynine were used as scaffolds for analog synthesis. Analogs with pseudo-indoxyl (PI) rearrangements or hydroxyl group additions were made for both compounds, and a naturally occurring minor kratom alkaloid and speciogynine isomer, speciociliatine, was also synthesized for testing. **(A)** Chemical structures of selected indole-based kratom alkaloids; **(B)** synthesis of 7-hydroxypaynantheine (7) and paynantheine pseudoindoxyl (8); and **(C)** synthesis of 7-hydroxyspeciogynine (9) and speciogynine pseudoindoxyl (10). 7-hydroxyspeciogynine (7OH SPG, 9), 7-hydroxypaynantheine (7OH PAYN, 7), and speciociliatine (SPECIO, 4) are compared to kratom alkaloids (dashed lines; 7-hydroxymitragynine (7OH MITRA), paynantheine (PAYN), and speciogynine (SPG) for inhibition of forskolin-induced cAMP in a GloSensor assay in transfected HEK-293 cells at δOR **(D)** and μOR **(E)**. For additional *in vitro* characterization, see Supplemental Figure S4.

To assess drug-induced effects on ambulation for 7-hydroxymitragynine, locomotor activity was assessed in a 2-day protocol as previously described (Gutridge et al., 2020). To assess drug-induced effects on ambulation for paynantheine and 7-hydroxyspeciogynine, locomotor information was extracted from the data generated in the CPP experiments. Distance traveled during each drug and vehicle conditioning session were pulled from the 30- or 40-min conditioning session (extended or brief CPP paradigm, respectively), and all sessions per treatment were averaged for analysis. A summary of all statistical analyses for the locomotor data can be found in Supplemental Table S2. In brief, for 7-hydroxymitragynine locomotor data in Figure 1, an unpaired, two-tailed *t* test was used. For paynantheine locomotor data in Figure 2G, statistical significance of drug treatment vs. vehicle was obtained by a one-way ANOVA with Dunnett’s multiple comparisons to VEH + VEH. For paynantheine + morphine locomotor data in Figure 2G, statistical significance of paynantheine + morphine vs. morphine alone was obtained *via* a one-way ANOVA with Dunnett’s multiple comparisons to morphine (MOR). For 7-hydroxyspeciogynine locomotor data in Figure 3B, a two-tailed, paired *t* test was used; one mouse was removed from this analysis after being identified as an outlier with Grubb’s test.

#### Brief and Extended Conditioned Place Preference Paradigms

Mice were conditioned to drugs and vehicle as described previously in two-chamber conditioned place preference (CPP) boxes in a counterbalanced, unbiased approach for either two drug conditioning sessions over 2 days (brief) or four drug conditioning sessions over 8 days (extended) (Váradi et al., 2015; Gutridge et al., 2020). For brief and extended conditioned place preference experiments, separate groups of mice were used for each drug dose. A summary of all statistical analyses for the CPP data can be found in Supplemental Table S4. In brief, all CPP data were analyzed with two-tailed, paired t tests comparing time spent on the drug-paired side pre- and post-conditioning.

#### Seizure Assay

To assess drug-induced seizurogenic activity, mice were placed in a clear plastic cylinder (25 cm diameter, 35 cm height) immediately following drug injection and their activity was recorded in a well-lit, quiet room using iSpy camera software (iSpyConnect.com). A recording time of 90 min was chosen for the tested compounds based on previous observations of seizures time lengths in experiments with 30 mg∙kg^−1^ paynantheine. If animals were not presenting with seizure activity after 30 min, the recording time was shortened accordingly. Seizure severity was scored based on the modified Racine scale (half-scores allowed) in bins of 3–5 min. Onset to first seizure symptom, onset to highest Racine score, and highest Racine score were also assessed. A summary of all statistical analyses for the seizure data can be found in Supplemental Table S3. In brief, seizure-like behavior between wild type and δOR KO mice was compared with a two-tailed, unpaired *t* test with Welch’s correction on area under the curve data generated from graphing the highest Racine score per time bin over 90 min for each mouse.

#### Tail Flick Thermal Nociception Assay

Antinociception *via* the tail flick assay was measured as previously described (van Rijn et al., 2012). Mice were first habituated to the handling restraint used during the experimentation. On subsequent test days, a radiant heat tail flick instrument (Columbus Instruments, Columbus, OH, United States) was used to collect duplicate measurements by testing two different regions on the mouse’s tail. The beam intensity was adjusted between each group of mice to elicit reproducible responses between 2 and 3 s (beam intensity of 7–9). At a minimum, mice were given 2 days between experiments to recover from thermal stimuli. For each test day, a baseline tail flick response was collected for each mouse and was used to calculate the testing cutoff time (cutoff time = three times the baseline response time). To test antinociception by drug agonism, a vehicle injection was administered next (i.p. or s.c.), and tail flick responses were collected after 30 min. The drug was then administered (i.p. or s.c.), and tail flick responses were collected after 30 min. To test drug antagonism of morphine antinociception, a response to vehicle injections were similarly collected prior to drug administration with a first vehicle injection (i.p. or s.c.) at 0 min, followed by a second vehicle injection (s.c.) 10 min before collecting tail flick responses at 30 min (20 min after the second vehicle injection). The test compound was then administered (i.p. or s.c.), followed by 6 mg∙kg^−1^ morphine (s.c.) 10 min later. Tail flick responses were collected 20 min after morphine administration. Data are represented as percent maximal possible effect (%MPE) and is calculated as %MPE = (treatment response time − baseline response time)/(cutoff time − baseline response time) * 100. Data are normalized to vehicle treatment: drug treatment %MPE − saline treatment %MPE. A summary of all statistical analyses for the antinociceptive data can be found in Supplemental Table S5. In brief, for agonist antinociception assays, significance was calculated *via* a two-tailed, paired *t* test to compare vehicle and drug treatment. For antagonist antinociception assays with three treatment groups in the same group of mice (Figure 2D), data were analyzed *via* repeated measures (RM) one-way ANOVA with Dunnett’s multiple comparisons to the morphine-only treatment group. For antagonist antinociception assays with two treatment groups in two different groups of mice (Figure 3D), an unpaired *t*-test with Welch’s correction was used to assess significance between the morphine-only group and the morphine plus “antagonist” group.

#### Two-Bottle Choice Alcohol Paradigm

Mice were subject to drinking in the dark (DID), limited access (4 h per day), two-bottle choice (10% ethanol vs. water) paradigm in which they were trained to consume alcohol voluntarily as previously described (Rhodes et al., 2005; van Rijn and Whistler, 2009). Mice reached stable alcohol consumption within 3 weeks of training, and after the third week, drug injections were administered prior to the daily drinking session on Friday. Drug’s effect on alcohol consumption was measured as the change in Friday’s alcohol intake minus the average alcohol intake from the preceding Tuesday–Thursday of that week (g∙kg^−1^). A summary of all statistical analyses for the drinking data can be found in Supplemental Tables S6–S9. In brief, results from two-bottle choice alcohol consumption paradigms were assessed for statistical significance using RM two-way ANOVA for main effects of drug dose, treatment day, and drug dose × treatment day; Sidak’s multiple comparisons (MC) between alcohol consumption baseline (Tuesday–Thursday average) vs. treatment day consumption (Friday) were then used as the post hoc test for each drug dose tested. The same RM two-way ANOVA and Sidak’s MC *post hoc* analyses were used for water consumption and ethanol preference data. For the change in alcohol consumption, change in water consumption, and change in ethanol preference data for 7-hydroxyspeciogynine where male and female data were analyzed together, a mixed-effects model was used (due to missing values) with the Geisser–Greenhouse correction for main effects, followed by Dunnett’s MC between alcohol consumption baseline vs. treatment day consumption. Sex differences between baseline data were evaluated using RM two-way ANOVA for main effects of sex, treatment baseline, and sex × treatment baseline; Sidak’s multiple comparisons (MC) between male and female mice were then used as the post hoc test for each treatment week tested.

#### Accelerating Rotarod Test

Mice were trained to walk on a rotarod apparatus (IITC, United States) with 1.25” diameter drums 2 days prior to drug testing. The rotarod started at 3 rpm and was increased to 30 rpm over 300 s. A trial for a mouse ended when it fell and tripped the sensor, when it rode the rotarod for two consecutive revolutions, or after 300 s (the maximum trial time)(White et al., 2015). Mice received at least 3 min of rest between trials. On test day, baseline performance was assessed as the average latency to fall in three trials per mouse. Mice were then injected with 30 mg∙kg^−1^ speciociliatine (i.p.) and immediately tested for performance on the apparatus (this first data point represented as latency to fall at 5 min), and then tested again at 15, 30, 60, and 120 min post-injection. Each mouse’s performance was normalized to its own baseline and reported as a percentage. A summary of all statistical analyses for the rotarod data can be found in Supplemental Table S2. In brief, data for each tested timepoint were calculated as a percentage of the baseline, and thus statistical significance was calculated in a two-tailed, one sample *t* test vs. a hypothetical mean of 100 (baseline was 100%). Rotarod results between WT and δOR KO genotypes were compared with a mixed-effects model with fixed effects for timepoint, genotype, and timepoint × genotype.

### Nomenclature of Targets and Ligands

Key protein targets and ligands in this article are hyperlinked to corresponding entries in http://www.guidetopharmacology.org, the common portal for data from the IUPHAR/BPS Guide to PHARMACOLOGY (Harding et al., 2018), and are permanently archived in the Concise Guide to PHARMACOLOGY 2019/20 (Alexander et al., 2019).

## Results

### Hyperlocomotion Induced by the Kratom Alkaloid 7-Hydroxymitragynine Is Naloxone-Reversible

The kratom alkaloid 7-hydroxymitragynine was the most potent amongst kratom alkaloids in decreasing alcohol intake (Gutridge et al., 2020); however, it produces significant adverse effects in such as conditioned place preference and hyperlocomotion. This hyperlocomotion induced by 7-hydroxymitragynine was blocked by a low, 1 mg∙kg^−1^ dose of naloxone (unpaired, two-tailed *t* test, t = 5.441, df = 8, *p* = 0.0006) (Figure 1).

### Paynantheine Functionally Antagonizes Morphine Effects *in vivo*

Paynantheine is a naturally occurring G-protein–biased kratom alkaloid with micromolar potency and affinity at the μOR and δOR that dose-dependently decreases alcohol intake in male mice at 10 and 30 mg∙kg^−1^, but unlike 7-hydroxymitragynine does not produce hyperlocomotion at its effective dose (Gutridge et al., 2020). In contrast to 7-hydroxymitragynine, paynantheine produces modest conditioned place aversion (CPA) in a brief CPP paradigm (paired, two-tailed *t* test, t = 2.606, df = 7, *p* = 0.0351) (Figure 2A). However, when using an extended CPP paradigm paynantheine did not produce CPP nor CPA (paired, two-tailed *t* test, t = 2.227, df = 7, *p* = 0.0612) (Figure 2B). Additionally, we observed Racine level 1–2 convulsive behaviors in wild type and δOR KO mice injected with a 30 mg∙kg^−1^ dose (Figure 2C) with no difference between groups (Welch’s *t* test, t = 0.9205, df = 6.738, *p* = 0.3891). In the GloSensor assay of cAMP inhibition, paynantheine displayed partial to full agonism at the ORs (Gutridge et al., 2020) (Supplemental Table S1); however, paynantheine has also been reported as weak antagonist in a BRET-based G-protein assay at human ORs (Kruegel et al., 2016). To obtain a better understanding of paynantheine’s pharmacology *in vivo*, we assessed if paynantheine was antinociceptive in thermal nociception paradigms. Though the 30 mg∙kg^−1^ dose of paynantheine produced a statistically significant difference in %MPE vs. vehicle (paired, two-tailed *t* test, t = 2.925, df = 9, *p* = 0.0169), neither the 10 nor 30 mg∙kg^−1^ dose displayed meaningful antinociceptive effects (Figure 2D, first two columns). Instead, paynantheine dose-dependently blocked antinociception produced by 6 mg∙kg^−1^ morphine (RM one-way ANOVA, overall effect: F (1.943,17.49) = 12.38, *p* = 0.0005, with Dunnett’s MC to 6 mg∙kg^−1^ morphine: *p* = 0.6330 for 10 mg∙kg^−1^ dose, *p* = 0.0019 for 30 mg∙kg^−1^ dose) (Figure 2D, last three columns). Because paynantheine blocked morphine action in a nociception assay and by itself did not produce CPP, we next sought to determine if it could block morphine CPP. However, neither pretreatment with 10 nor 30 mg∙kg^−1^ paynantheine abolished 6 mg∙kg^−1^ morphine CPP (paired, two-tailed t tests, t = 3.214, df = 7, *p* = 0.0148 for the 10 mg∙kg^−1^ dose, t = 6.609, df = 5, *p* = 0.0012 for the 30 mg∙kg^−1^ dose) (Figure 2E). However, when assessing locomotor data from the CPP experiments in Figures 2A,E, we did observe that paynantheine dose-dependently attenuated hyperlocomotion induced by 6 mg∙kg^−1^ morphine (one-way ANOVA, overall effect: F (2,15) = 39.25, *p* < 0.0001, with Dunnett’s MC to 6 mg∙kg^−1^ morphine: *p* = 0.0004 for 10 mg∙kg^−1^ dose, *p* < 0.0001 for 30 mg∙kg^−1^ dose) (Figures 2F,G).

### Kratom Analogs Are OR Partial Agonists With Minimal β-Arrestin-2 Recruitment

In order to produce better lead candidates to treat alcohol use disorder that lack adverse locomotor and rewarding effects, we next aimed to discover kratom alkaloids or alkaloid derivatives with increased δOR affinity and potency but with limited µOR potency. To this end, we extracted paynantheine (2), speciogynine (3), and speciociliatine (4) from dry kratom powder using a modified protocol reported by Varadi et al., 2016. Paynantheine (2) was converted to 7-hydroxypaynantheine (7), (Figure 3B) using PIFA in acetonitrile and water. This 7-hydroxypaynantheine was next transformed to paynantheine pseudoindoxyl (8) using Zn(OTf)_2_ in refluxing toluene. We adopted the same strategy to synthesize 7-hydroxyspeciogynine (9) and speciogynine pseudoindoxyl (10) as shown in Figure 3C.

Affinity wise, we noted that the paynantheine analogs, especially the 7-hydroxyl analog, showed weak µOR affinity, whereas 7-hydroxyspeciogynine displayed the strongest µOR affinity (Table 1 and Supplementary Figure S3A). At the δOR, 7-hydroxyspeciogynine displayed improved binding relative to speciogynine, which was on par with affinities for the two pseudoindoxyl analogs. 7-hydroxypaynantheine was a magnitude weaker in binding the δOR than 7-hydroxyspeciogynine; this same trend was apparent at the κOR (Table 1 and Supplementary Figures S4A–C).

In terms of cAMP inhibition, we noted clear signs of partial agonism for the analogs at the µOR, with paynantheine pseudoindoxyl, 7-hydroxypaynantheine, and 7-hydroxyspeciogynine displaying the lowest potency at the µOR (Figures 3D,E; Table 1, Supplementary Figure S3A). 7-hydroxyspeciogynine was the strongest activator at the δOR (Figure 3D), whereas speciociliatine exhibited the strongest κOR potency out of the tested alkaloids (Table 1 and Suppementary Figure S3F). Notably, while speciociliatine displayed binding at the δOR, it showed minimal activity at this receptor in regards to cAMP inhibition, suggestive of it acting as antagonist at the δOR (Table 1 and Supplemental Figures S3B,E). At the κOR, we did not detect cAMP inhibition for 7-hydroxypaynantheine at the tested dose range (Table 1 and Supplementary Figure S3F). We did not detect any β-arrestin-2 recruitment for speciociliatine and the pseudoindoxyl and 7-hydroxyl analogs within the tested dose range (Table 1 and Supplementary Figures 3G–I), which is line with the reported G-biased nature of the kratom alkaloids (Kruegel et al., 2016; Váradi et al., 2016; Gutridge et al., 2020).

### Speciociliatine Modulation of Alcohol Intake Is Compounded by Drug-Induced Locomotor Incoordination

**FIGURE 4:**
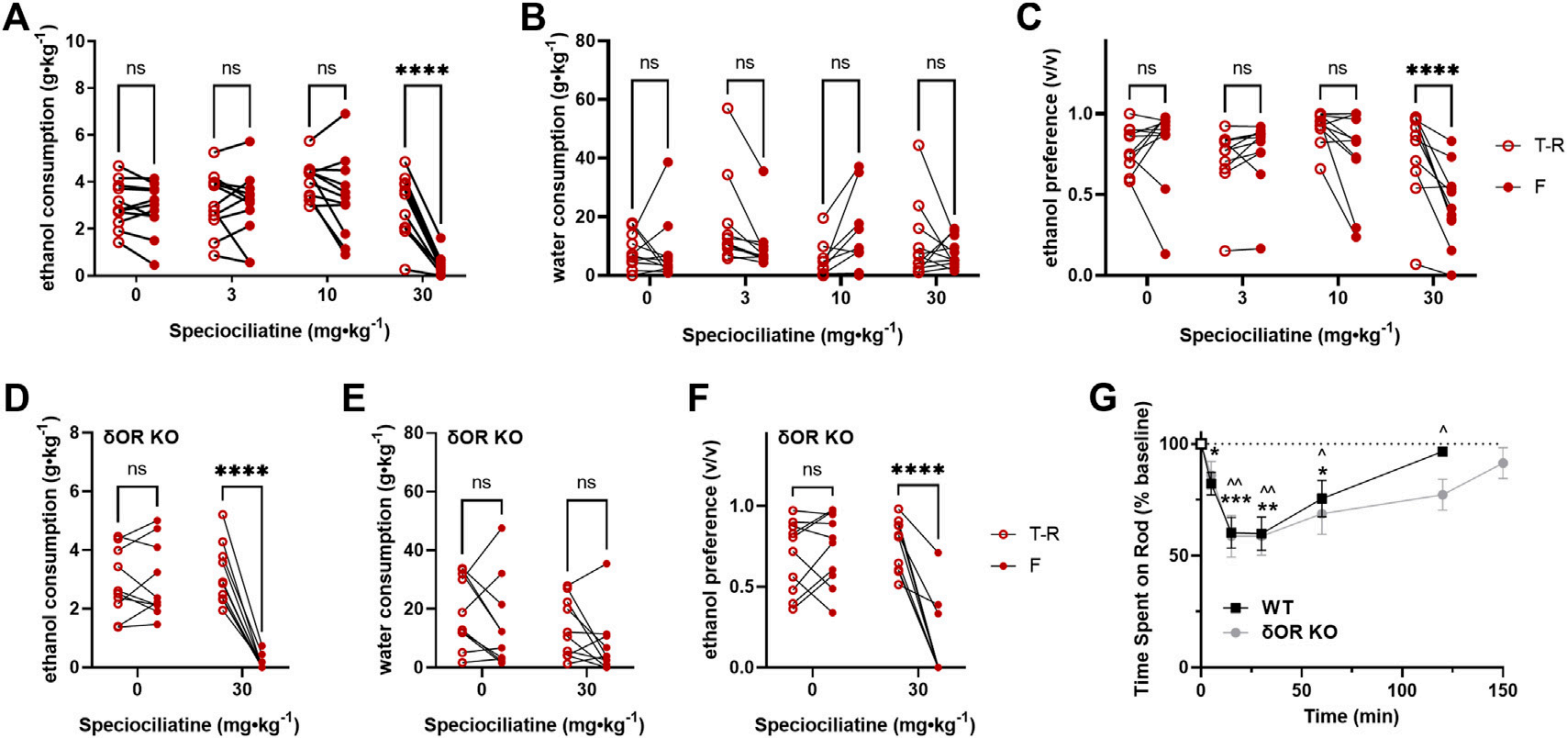
Speciociliatine decreases voluntary ethanol consumption and impairs motor coordination in wild-type and δOR knockout mice. 10% ethanol consumption, water consumption and ethanol preference in male C57BL/6 **(A–C**, respectively) (*n* = 11) and δOR KO **(D–F**, respectively) mice (*n* = 10) in a voluntary twobottle choice, limited access, drinking-in-the-dark paradigm, following treatment with speciociliatine (3, 10, and 30 mg⋅kg⁻^1^, i.p.) **(G)** 150-minute duration rotarod assessment of motor incoordination inWTmice (*n* = 8) and δOR KO mice (*n* = 7), immediately followed by a 30 mg⋅kg⁻^1^ dose of speciociliatine (i.p.); significance for WT mice and δOR KO mice is denoted with stars and carets, respectively. Open circles are the average intake/preference on the preceding 3 days (baseline), and closed circles are the intake on Fridays following drug exposure. * or^*p* < 0.05, ** or^*p* < 0.01, ***p < 0.001, ****p < 0.0001 (for details, see Supplemental Tables S6–S8).

Based on our hypothesis that G-protein–biased δOR agonism drives decreased alcohol intake following kratom alkaloid injection, we did not expect speciociliatine to decrease alcohol intake as it behaves *in vitro* as a partial agonist for μOR and κOR but antagonist at δOR (Table 1). However, speciociliatine significantly decreased ethanol consumption but only at the 30 mg∙kg^−1^ dose (RM two-way ANOVA, dose: F (3, 30) = 36.48, *p* < 0.0001, time: F (1, 10) = 50.17, *p* < 0.0001, dose × time: F (3, 30) = 13.30, *p* < 0.0001, with Sidak’s MC (T-R vs F), *p* < 0.0001 for the 30 mg∙kg^−1^ dose) (Figure 4A) and with surprisingly strong efficacy (an average decrease of 2.5 ± 0.3 g∙kg^−1^ ethanol or a 90 ± 3% reduction, Supplementary Figure S5A). However, the 30 mg∙kg^−1^ dose demonstrated a similar alcohol modulating effect in δOR KO mice (RM two-way ANOVA, dose: F (1, 9) = 25.36, *p* = 0.0007, time: F (1, 9) = 61.69, *p* < 0.0001, dose × time: F (1, 9) = 83.26, *p* < 0.0001, with Sidak’s MC (T-R vs F), *p* < 0.0001 for the 30 mg∙kg^−1^ dose) (Figure 4D). Treatment with speciociliatine did not change water consumption at any of the tested doses in wild type or δOR KO mice (Figures 4B,E, respectively). Taking together the lack of compensatory increase in water consumption and the decrease in ethanol consumption at the 30 mg∙kg^−1^ dose, the ethanol preference was thus significantly decreased at this dose in wild-type mice (Figure 4C) (RM two-way ANOVA, dose: F (3, 30) = 24.20, *p* < 0.0001, time: F (1, 10) = 17.10, *p* = 0.002, dose × time: F (3, 30) = 7.521, *p* = 0.0007, with Sidak’s MC (T-R vs F), *p* < 0.0001 for the 30 mg∙kg^−1^ dose) and δOR KO mice (Figure 4F) (RM two-way ANOVA, dose: F (1, 9) = 32.58, *p* = 0.0003, time: F (1, 9) = 23.26, *p* = 0.0009, dose × time: F (1, 9) = 64.72, *p* < 0.0001, with Sidak’s MC (T-R vs F), *p* < 0.0001 for the 30 mg∙kg^−1^ dose). The 30 mg∙kg^−1^ dose also significantly reduced the ability of treated wild-type mice to perform in the rotarod assessment (Figure 4G). This motor effect had a rapid onset, where time spent on the device significantly decreased at 5 min (one sample *t* test, t = 3.478, df = 7, *p* = 0.0103), with the peak effect occurring between 15 and 30 min (t = 5.809, df = 7, *p* = 0.0007; t = 5.344, df = 7, *p* = 0.0011, respectively), and the mice fully recovering at 120 min (t = 1.953, df = 7, *p* = 0.0918). The same effect was observed in δOR KO mice (mixed effects model with matching for genotype × timepoint, F (1.941,11.26) = 1.930, *p* = 0.1906).

### Kratom Analogs Decrease Ethanol Consumption in a δOR-Dependent Mechanism

**FIGURE 5:**
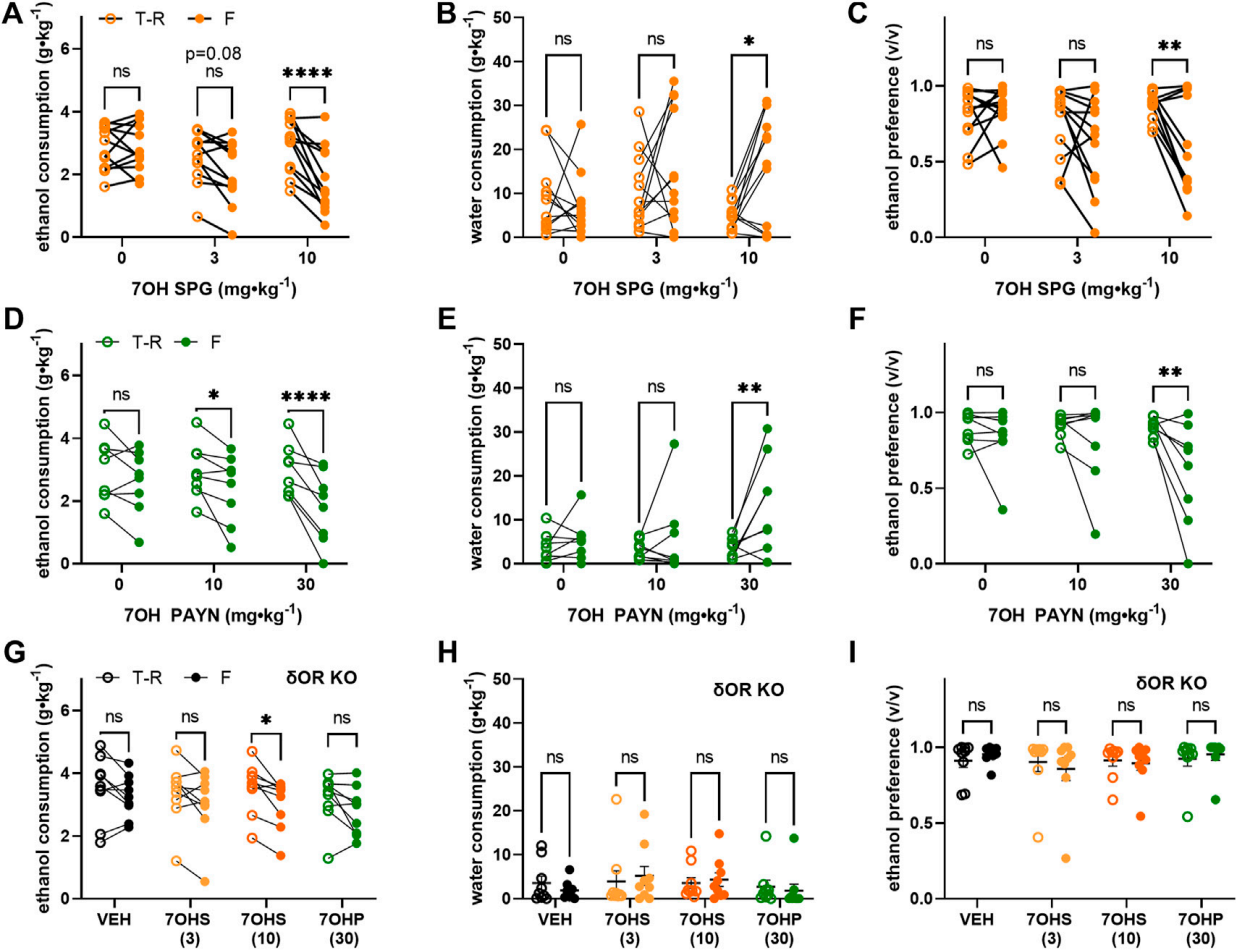
Kratom analogs decrease voluntary ethanol consumption in mechanisms partially dependent on δOR. 10% ethanol consumption (left column), water consumption (middle column), and ethanol preference (right column) in male C57Bl/6 wild-type mice following treatment with **(A–C)** 7-hydroxyspeciogynine (3 and 10 mg⋅kg^−1^, s.c., *n* = 12, 7OH SPG; 7OHS), **(D–F)** 7-hydroxypaynantheine (10 and/or 30 mg⋅kg^− 1^, s.c., *n* = 8, 7OH PAYN; 7OHP), and in **(G–I)** male δOR KO mice (*n* = 9), following treatment with effective doses of both analogs in a voluntary two-bottle choice, limited access, drinking-in-the-dark paradigm. Open circles are the average intake/preference on the preceding 3 days (baseline), and closed circles are the intake on Fridays following drug exposure. *p < 0.05, **p < 0.01, ****p < 0.0001 (for details, see Supplemental Tables S6–S8).

Given the weak µOR potency of 7-hydroxyspeciogynine and 7-hydroxypaynantheine but the clear 0.5–1 log-fold difference in potency at the δOR between the two analogs (Figures 3D,E), we next assessed the *in vivo* potency of these two alkaloids in modulating volitional alcohol consumption in mice. In wild-type male mice, 7-hydroxyspeciogynine more potently reduced alcohol intake in a dose-dependent manner at 3 and 10 mg∙kg^−1^ (Figure 5A, RM two-way ANOVA, dose: F (2, 22) = 6.973, *p* = 0.0045, time: F (1, 7) = 13.79, *p* = 0.0006, dose × time: F (2, 22) = 8.675, *p* = 0.0017, with Sidak’s MC (T-R vs F), *p* = 0.0802 for the 3 mg∙kg^−1^ dose, *p* < 0.0001 for the 10 mg∙kg^−1^ dose). This decrease in ethanol consumption at the 10 mg∙kg^−1^ dose was accompanied by a concomitant increase in water consumption during the time course of the voluntary alcohol consumption paradigm (Figure 5B, RM two-way ANOVA, dose: F (2, 22) = 8.706, *p* = 0.0016, time: F (1, 11) = 4.161, *p* = 0.0661, dose × time: F (2, 22) = 3.489, *p* = 0.0483, with Sidak’s MC (T-R vs F), *p* = 0.0112) as well as a corresponding decrease in ethanol preference (Figure 5C, RM two-way ANOVA, dose: F (2, 22) = 9.997, *p* = 0.0008, time: F (1, 11) = 8.284, *p* = 0.0150, dose × time: F (2, 22) = 4.140, *p* = 0.0298, with Sidak’s MC (T-R vs F), *p* = 0.0036). We found that 7-hydroxypaynantheine was able to significantly reduce alcohol intake at a 10 and 30 mg∙kg^−1^ dose (Figure 5D, RM two-way ANOVA, dose: F (2, 14) = 4.200, *p* = 0.0373, time: F (1, 7) = 13.79, *p* = 0.0075, dose × time: F (2, 14) = 5.515, *p* = 0.0171, with Sidak’s MC (T-R vs F), *p* = 0.0219 for the 10 mg∙kg^−1^ dose, *p* < 0.0001 for the 30 mg∙kg^−1^ dose). Similarly, the decrease in ethanol consumption at the 30 mg∙kg^−1^ dose of 7-hydroxypaynantheine was accompanied by a concomitant increase in water consumption during the time course of the voluntary alcohol consumption paradigm (Figure 5E, RM two-way ANOVA, dose: F (2, 14) = 4.129, *p* = 0.0389, time: F (1, 7) = 4.920, *p* = 0.0621, dose x time: F (2, 14) = 4.149, *p* = 0.0385, with Sidak’s MC (T-R vs F), *p* = 0.0015) and a corresponding decrease in ethanol preference (Figure 5F, RM two-way ANOVA, dose: F (2, 14) = 3.845, *p* = 0.0467, time: F (1, 7) = 5.193, *p* = 0.0567, dose × time: F (2, 14) = 3.980, *p* = 0.0428, with Sidak’s MC (T-R vs F), *p* = 0.0036). In δOR KO mice subject to the same voluntary alcohol consumption paradigm, 10 mg∙kg^−1^ 7-hydroxyspeciogynine significantly decreased ethanol consumption (RM two-way ANOVA, dose: F (4, 32) = 6.407, *p* = 0.0007, time: F (1, 8) = 16.46, *p* = 0.0036, dose × time: F (4, 32) = 1.851, *p* = 0.1435, with Sidak’s MC (T-R vs F), *p* = 0.0269) but not the 3 mg∙kg^−1^dose of 7-hydroxyspeciogynine or the 30 mg∙kg^−1^dose of 7-hydroxypaynantheine (Figure 5G). Water consumption (Figure 5H) and ethanol preference (Figure 5I) were not significantly changed in the δOR KO mice following treatment with the kratom analogs.

**FIGURE 6:**
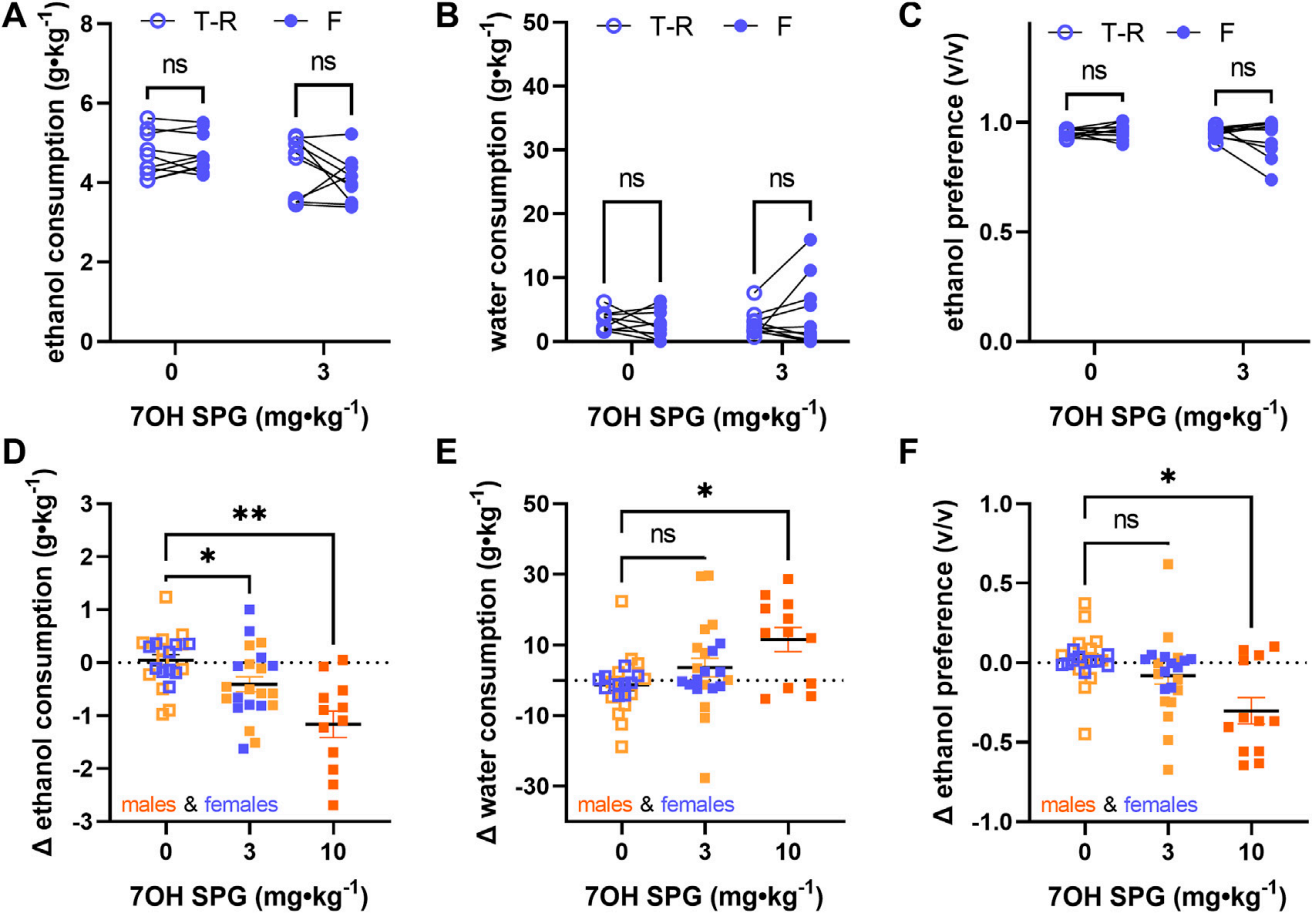
Alcohol-modulating effects of 3 mg⋅kg⁻^1^ 7-hydroxyspeciogynine are not sex specific. In WT female mice (*n* = 10), effects of 3 mg⋅kg⁻^1^ 7-hydroxyspeciogynine (s.c., 7OG SPG) on 10% ethanol consumption **(A)**, water consumption **(B)**, and ethanol preference **(C)** were evaluated in a voluntary two-bottle choice, limited access, drinking-in-the-dark paradigm. Male and female responses to 7-hydroxyspeciogynine (3 and 10 mg⋅kg⁻^1^, s.c.) in the two-bottle choice paradigm were pooled and are shown as **(D)** change (Δ) in 10% ethanol consumption, **(E)** change (Δ) in water consumption, and **(F)** change (Δ) in ethanol preference. In panels **(A–C)**, open circles are the average intake/preference on the preceding 3 days (baseline), and closed circles are the intake on Fridays following drug exposure. In panel **(D–F)**, female and male mice are depicted with blue and orange symbols, respectively. *p < 0.05, **p < 0.01 (for details, see Supplemental Tables S6–S9).

In female mice exposed to the voluntary alcohol consumption paradigm, 7-hydroxyspeciogynine did not significantly modulate ethanol consumption, water consumption, or ethanol preference at the 3 mg∙kg^−1^ dose (Figures 6A–C; see Supplemental Tables S6–S8 for statistical analyses). As previously reported (Rhodes et al., 2005), female mice exhibit a significantly higher baseline of alcohol consumption compared to males (Supplemental Table S9, RM two-way ANOVA, sex: F (1, 20) = 39.05, *p* < 0.0001, time: F (1, 20) = 6.295, *p* = 0.0208, dose × time: F (1, 20) = 0.1027, *p* = 0.7520, with Sidak’s MC (male vs female), *p* < 0.0001 for the vehicle treatment baseline, *p* < 0.0001 for the 3 mg∙kg^−1^ 7-hydroxyspeciogynine treatment baseline). However, no sex difference was apparent in the Δ ethanol intake (Supplemental Table S9, RM two-way ANOVA, sex: F (1, 20) = 0.1974, *p* = 0.6616, dose: F (1, 20) = 7.758, *p* = 0.0114, sex × dose: F (1, 20) = 0.2487, *p* = 0.6234, with Sidak’s MC (male vs female), *p* = 0.9993 for the Δ ethanol consumption following vehicle treatment, *p* = 0.7635 for the Δ ethanol consumption following 3 mg∙kg^−1^ 7-hydroxyspeciogynine treatment). Combining the Δ ethanol intake for males and females, we found that there was a significant ethanol modulation effect at the 3 mg∙kg^−1^ dose when collectively analyzing male and female responses (Figure 6D, Mixed effects model (REML) with Geisser–Greenhouse correction, main effect of treatment: F (1.539, 40.80) = 13.36, *p* = 0.0001, with Dunnett’s MC (treatment vs vehicle), *p* = 0.0165 for the 3 mg∙kg^−1^ dose, *p* = 0.0064 for the 10 mg∙kg^−1^ dose). After finding similar sex differences in water consumption and ethanol preference but not in the Δ of these parameters (see, Supplemental Table S9 for details), pooled male and female responses were similarly analyzed for Δ in response of water consumption and ethanol preference. In the pooled data, a concomitant increase in water consumption was evident at a 10 mg∙kg^−1^ dose (Figure 6E, Mixed effects model (REML) with Geisser–Greenhouse correction, main effect of treatment: F (1.733, 27.74) = 5.978, *p* = 0.0091, with Dunnett’s MC (treatment vs vehicle), *p* = 0.1804 for the 3 mg∙kg^−1^ dose, *p* = 0.0342 for the 10 mg∙kg^−1^ dose). Accordingly, in the pooled data, a significant decrease in ethanol preference was noted at the 10 mg∙kg^−1^ dose (Figure 6F, Mixed effects model (REML) with Geisser–Greenhouse correction, main effect of treatment: F (1.645, 43.58) = 7.889, *p* = 0.0022, with Dunnett’s MC (treatment vs vehicle), *p* = 0.1644 for the 3 mg∙kg^−1^ dose, *p* = 0.0255 for the 10 mg∙kg^−1^ dose).

### 7-Hydroxyspeciogynine Has Limited Side Effects Due to Its Decreased µOR-Dependent Pharmacology

**FIGURE 7:**
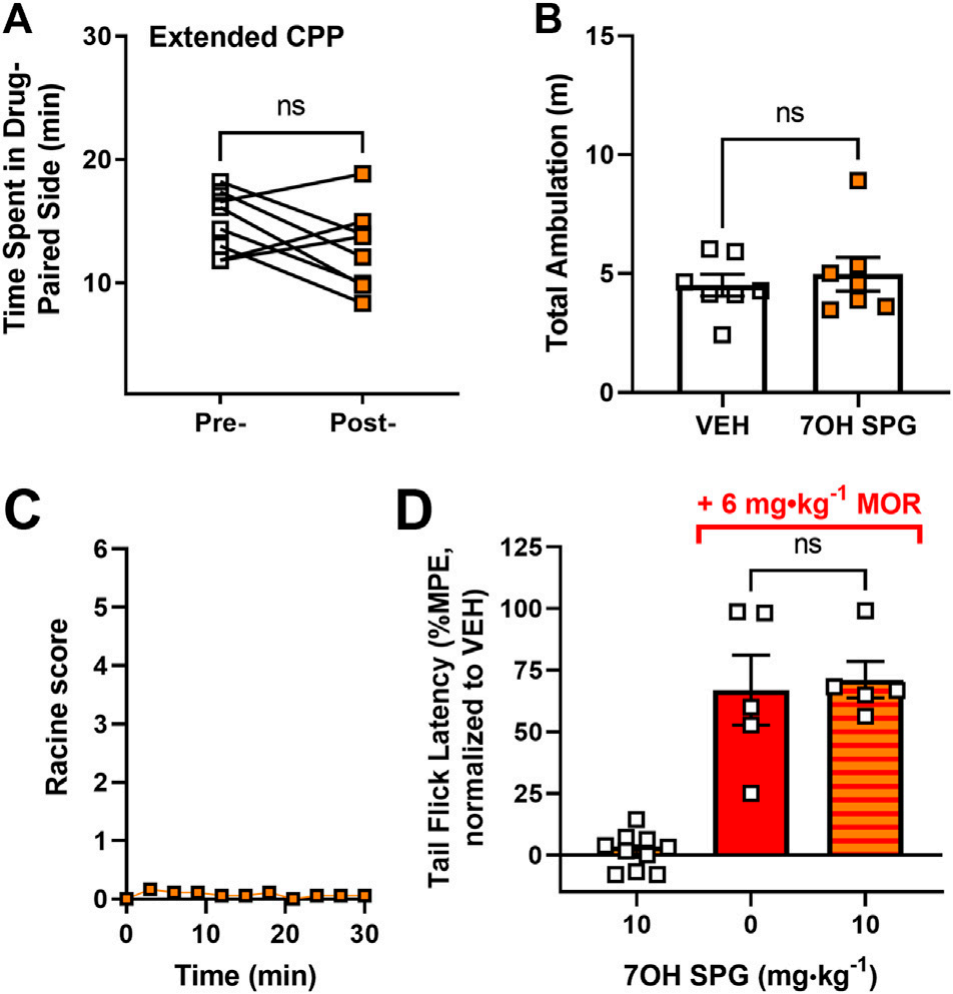
Side effect profile of 10 mg⋅kg⁻^1^ 7-hydroxyspeciogynine. **(A)** In a 10-day conditioned place preference (CPP) paradigm, the rewarding effects of 7-hydroxyspeciogynine (s.c.) were evaluated in male, WT mice (*n* = 8). **(B)** Locomotor data were extracted from the CPP experiment in **(A)** and averaged across all vehicle/ drug treatment days (*n* = 7). **(C)** The highest Racine score collected every 3 min for 30 min following administration of 7-hydroxyspeciogynine was evaluated for 30 min after drug administration (*n* = 9). **(D)** 7-hydroxyspeciogynine was tested for agonist and analgesic properties in male mice *via* the tail flick thermal nociception assay (*n* = 10). In the same paradigm, antagonistic effects were evaluated after administering 7-hydroxypeciogynine, followed by morphine (6 mg⋅kg⁻^1^, s.c.) 10 min later (*n* = 6), and were compared to vehicle plus morphine administration (*n* = 5) (for statistical details, see Supplemental Tables S2–S5).

From the cellular and behavioral experiments, 7-hydroxyspeciogynine emerged as the most promising kratom-derived analog for reducing alcohol use, with relatively equal *in vivo* potency as 7-hydroxymitragynine at the δOR but lower µOR potency. Next, we assessed whether 7-hydroxyspeciogynine exhibited a better side effect profile than 7-hydroxymitragynine due to its limited potency at the µOR. Additionally, to determine if 10 mg∙kg^−1^ 7-hydroxyspeciogynine was the maximum tolerated dose (MTD), we assessed the side effect profile for the 10 mg∙kg^−1^ dose. We found that mice treated with 10 mg∙kg^−1^ 7-hydroxyspeciogynine did not develop conditioned place preference in our “extended” conditioned place preference protocol, which involves four conditioning sessions each for drug and vehicle (paired, two-tailed *t* test, t = 1.592, df = 7, *p* = 0.1554) (Figure 7A). The same 10 mg∙kg^−1^ dose of 7-hydroxyspeciogynine did not significantly alter ambulation (paired, two-tailed *t* test, t = 0.7552, df = 6, *p* = 0.4787) (Figure 7B) or induce seizures (Figure 7C). Akin to 10 mg∙kg^−1^ paynantheine, 10 mg∙kg^−1^ 7-hydroxyspeciogynine did not produce antinociception (paired, two-tailed *t* test, t = 0.6193, df = 9, *p* = 0.5511) or block morphine analgesia (unpaired *t* test with Welch’s correction, t = 0.2660, df = 5.994, *p* = 0.7991) (Figure 7D).

## Discussion

Over the past decade, kratom has been reported as a source for naturally occurring, G-protein–biased opioidergic alkaloids, and has been investigated for its effects on pain management (Matsumoto et al., 2004; Kruegel et al., 2019; Chakraborty et al., 2021b; Chakraborty and Majumdar, 2021), opioid withdrawal (Wilson et al., 2020, 2021), and alcohol abuse (Gutridge et al., 2020) as well as its decreased reward profile relative to traditional opioids (Hemby et al., 2019; Wilson et al., 2021). Here, we further probed the effects of kratom alkaloids and synthetic kratom alkaloid derivatives to obtain a better understanding of its *in vivo* pharmacology and in search of novel treatment options for alcohol use disorder. We report 7-hydroxyspeciogynine as an effective lead compound to reduce alcohol with an MTD of at least 10 mg∙kg^−1^.

We previously demonstrated that 7-hydroxymitragynine as well as paynantheine could decrease alcohol consumption (Gutridge et al., 2020). However, we were unable to obtain a MTD for 7-hydroxymitragyinine as it caused both hyperlocomotion and CPP at a 3 mg∙kg^−1^ dose, which was the minimal effective dose to reduce alcohol intake (Gutridge et al., 2020). It has been well-established that µOR agonism can cause CPP, and that these rewarding effects can be blocked by μOR antagonists (Negus et al., 1993; Piepponen et al., 1997) as well as μOR KO (Matthes et al., 1996). Here, we show that 7-hydroxymitragynine–induced hyperlocomotion also appears to be μOR-mediated as it is completely blocked by a dose of naloxone considered to be μOR-selective (Takemori and Portoghese, 1984; Pastor et al., 2005). Since the alcohol-reducing effect of 7-hydroxymitragynine was dependent on δORs (Gutridge et al., 2020), μOR potency may be a liability when exploring kratom alkaloids as treatment option for AUD. Paynantheine has much lower μOR potency, while retaining δOR potency and decreases alcohol intake in mice at a 10 mg∙kg^−1^ dose without causing hyperlocomotion (Gutridge et al., 2020). In line with the lower μOR potency, we find that 10 mg∙kg^−1^ paynantheine does not produce place preference in an extended CPP paradigm. In a brief CPP paradigm, however, the same dose of paynantheine induces conditioned place aversion (CPA). Kratom use can lead to seizures (Coonan and Tatum, 2021) and we noticed that at 30 mg∙kg^−1^, paynantheine induced minor seizure activity. It is possible that mice administered a dose of 10 mg∙kg^−1^ paynantheine did not feel well despite not showing overt tonic-clonic seizure activity that could contribute to the observed CPA at this dose. δOR agonism can cause seizures (Hong et al., 1998; Broom et al., 2002; Jutkiewicz et al., 2006); however, it is reported mostly for δOR agonists that are strong recruiters of β-arrestin, such as SNC80 and BW373U86 (O’Neill et al., 1997; Hong et al., 1998; Jutkiewicz et al., 2005). As such, we were not surprised that the G-protein–biased paynantheine-induced seizures were still present in δOR KO mice, indicating the seizures may be caused by an off-target interaction. Paynantheine can decrease alcohol consumption in wild-type mice (Gutridge et al., 2020); however, it also decreases alcohol consumption in δOR KO mice (Supplementary Figure S5; RM two-way ANOVA, dose: F (4, 32) = 6.407, *p* = 0.0007, time: F (1, 8) = 16.46, *p* = 0.0036, dose × time: F (4, 32) = 1.851, *p* = 0.1435, with Sidak’s MC (T-R vs F), *p* < 0.0001). This analysis provides further evidence that many of paynantheine’s *in vivo* effects are not mediated by δOR.

While antinociception has been reported for 7-hydroxymitragynine, the weaker μOR affinity alkaloid mitragynine reportedly lacks antinociceptive ability, and has been suggested to act as a μOR antagonist (Obeng et al., 2021); although in the cAMP assay, we previously identified mitragynine as a partial agonist (Gutridge et al., 2020), which is in line with a couple of other reports (Kruegel et al., 2016; Váradi et al., 2016). Paynantheine has weaker potency for the μOR than mitragynine in the cAMP assay but is more efficacious (Gutridge et al., 2020), which begged the question whether paynantheine possessed antinociceptive activity. However, both the 10 and 30 mg∙kg^−1^ doses of paynantheine failed to produce meaningful antinociception in the tail flick paradigm. In contrast, paynantheine blocks morphine analgesia at a 30 mg∙kg^−1^ dose but not at 10 mg∙kg^−1^, yet neither dose blocks morphine CPP. Additionally, paynantheine both at 10 and 30 mg∙kg^−1^ doses can block morphine hyper-ambulation. Paynatheine, at a 10 mg∙kg^−1^ dose, only blocks morphine hyper-ambulation within the first 15–20 min of the 40-min conditioning session. Detailed pharmacokinetic data for paynantheine have yet to be reported, but a recent study has shown that following oral administration in rats, a 1.1 mg∙kg^−1^ dose of paynantheine had a T_max_ of 10 min in plasma and was undetectable after an hour (Kamble et al., 2021). We suspect that in our hands paynantheine is similarly being rapidly metabolized and/or cleared from the brain and plasma, such that it may not block morphine’s CPP long enough to inhibit it significantly. This may also explain why the 10 mg∙kg^−1^ dose does not block morphine analgesia, which was tested at 20–30 min after administration. Furthermore, a day-by-day analysis of the locomotor activity revealed that the 30 mg∙kg^−1^ dose of paynantheine does not fully block morphine hyper ambulation within the last 5 min of the day 2 conditioning session (Supplementary Figures S1C,D). Because even one exposure to morphine is known to cause place preference in mice (Bardo and Neisewander, 1986), it is possible that mice administered with 30 mg∙kg^−1^ paynantheine experienced enough rewarding effects from morphine on day 2 to express CPP. However, since we did not measure CPP for 30 mg∙kg^−1^ paynantheine, we cannot rule out that paynantheine is responsible or positively contributed to the observed CPP. Taking together previous findings and the data collected here, we conclude that paynantheine is a weak partial agonist at the μOR and δOR, with functional antagonistic activity at the µOR in the presence of a more potent agonist *in vivo*. Overall, our conditioned place preference findings indicate that paynantheine has a low risk of reward, but its use may be limited by its low potency *in vivo*, and seizure effects that are not δOR-mediated.

We next decided to utilize the G-protein–biased nature of the kratom alkaloid scaffold to discover opioids that have increased δOR potency but that exhibits relatively low μOR potency. 7-hydroxymitragynine and mitragynine pseudoindoxyl, two previously characterized analogs of mitragynine, had higher δOR as well as µOR affinity and activity in cell lines compared to the indole-based template of mitragynine, and showed unique binding poses in computational models (Váradi et al., 2016; Zhou et al., 2021). To extend the structure–activity relationship (SAR) to the paynantheine and related speciogynine templates, we synthesized the hydroxylated and spiropseudoindoxyl variants of these natural products. We identified 7-hydroxyspeciogynine and 7-hydroxypaynantheine as having reduced µOR potency but similar δOR potency relative to 7-hydroxymitragynine. In contrast to the mitragynine-derived spiropseudoindoxyls, no advantage with respect to potency at the ORs was seen with the pseudoindoxyls derived from paynantheine or speciogynine. Both the novel 7-hydroxyl analogs dose-dependently decreased alcohol consumption, with 7-hydroxyspeciogynine displaying efficacious activity at a dose of 3 mg∙kg^−1^ and 7-hydroxypaynantheine at a 30 mg∙kg^−1^ dose. We confirmed that the alcohol-modulating effects of these analogs are at least partially acting through a δOR-mediated mechanism as we did not observe statistically significant reductions alcohol consumption in δOR KO mice for the two analogs at their effective doses. Because 7-hydroxyspeciogynine decreases ethanol consumption in δOR KO at a 10 mg∙kg^−1^dose but not 3 mg∙kg^−1^, this suggests that 7-hydroxyspeciogynine’s ethanol modulation is no longer solely mediated by δOR at higher doses.

Additionally, the *in vivo* potency of these compounds correlates well with their *in vitro* pharmacology at the δOR where 7-hydroxyspeciogynine is about 0.5–1 log-fold more potent than 7-hydroxypaynantheine (Table 1). While 7-hydroxyspeciogynine displays more potent activity at the μOR relative to 7-hydroxypaynantheine in the GloSensor assay (pIC_50_s of 6.2 ± 0.3 and 4.7 ± 0.5, respectively), the activity at this receptor is still less potent than 7-hydroxymitragynine (pIC_50_ = 7.8 ± 0.1). The G-protein–biased μOR activity of 7-hydroxyspeciogynine likely does not contribute to decreased alcohol use because of the lack of effect in δOR KO mice at the 3 mg∙kg^−1^ dose and because we have previously shown that selective activation of μOR G-protein signaling using Oliceridine/TRV130 did not decrease alcohol consumption (Gutridge et al., 2020).

Kratom-based natural products, including paynantheine and speciociliatine examined here, have been predicted and shown to have activity at adrenergic 2A, 2B, and 2C receptors and serotonin 2A receptors (Boyer et al., 2008; Ellis et al., 2020; Foss et al., 2020; Obeng et al., 2020; León et al., 2021). Since we did not screen the kratom analogs for activity at these or other receptors, it is probable that non-δOR activity contributes to the observed alcohol intake modulation, especially at higher doses. Though there is support for targeting adrenergic and serotonin receptors for treatment of alcohol abuse (Haass-Koffler et al., 2018; DiVito and Leger, 2020; Berquist and Fantegrossi, 2021; Sessa et al., 2021), our data about δOR KO animals shown here and in Gutridge et al., 2020 builds on our hypothesis of an ancillary, if not primary, role of δOR in decreasing alcohol consumption for kratom opioids and derivatives.

Relative to the GTPyS assay, the GloSensor assay of cAMP inhibition uses recombinant overexpressed cell systems and is amplified relative to measuring G-protein activity directly. As such, it is plausible that the partial agonism we detect for the kratom analogs *in vitro* does not resemble how they act *in vivo*. For example, at the δOR, mitragynine has partial agonism in the cAMP assay but acts as an antagonist in the GTPγS assay (Váradi et al., 2016; Gutridge et al., 2020). Therefore, it may be suggested that the kratom analogs are acting as functional δOR antagonists *in vivo*, competing with the fully efficacious activation of δORs by the endogenous Leu-enkephalin. However, our speciociliatine data counters this argument. At the δOR, speciociliatine binds with a pKi of 5.4 ± 0.1 which is in between the binding affinities of 7-hydroxyspeciogynine and 7-hydroxypaynantheine (6.3 ± 0.1 and 4.9 ± 0.2, respectively), yet speciociliatine acts as a δOR antagonist in the cAMP assay. When tested in mice, speciociliatine did cause a significant and sharp decrease in alcohol consumption at a relatively high 30 mg∙kg^−1^ dose (Supplementary Figure S4A–C, an average decrease of 2.5 ± 0.3 g∙kg^−1^ ethanol or a 90 ± 3% reduction, compared to a decrease of 1.2 ± 0.2 g∙kg^−1^ ethanol (40 ± 7%) for 10 mg∙kg^−1^ 7-hydroxyspeciogynine, and 1.1 ± 0.3 g∙kg^−1^ ethanol (40 ± 11%) for 30 mg∙kg^−1^ 7-hydroxypaynantheine), which indicates an off-target effect. In support of this explanation, a 30 mg∙kg^−1^ dose of speciociliatine similarly decreases ethanol consumption in δOR KO mice and significantly impairs motor incoordination in wild-type and δOR KO mice, which likely contributes to the effects we see in the alcohol consumption paradigm. We did not test the kratom analogs or alkaloids in conjunction with δOR antagonists because the role of δOR antagonists in these behaviors is not well defined. For example, we have previously found that δOR-selective antagonist naltrindole does not decrease alcohol intake at a 10 mg∙kg^−1^ dose in this alcohol model, whereas another δOR-selective antagonist, naltriben, dose-dependently decreases alcohol consumption at 6 and 10 mg∙kg^−1^ doses (van Rijn and Whistler, 2009). Although in rats, both naltrindole and naltriben decrease alcohol intake (Krishnan-Sarin et al., 1995a; 1995b). These discrepant responses may be explained by mediation of distinct δOR subtypes by these specific antagonists (Dietis et al., 2011; van Rijn et al., 2013). Therefore, evaluating alcohol consumption responses in δOR KO mice provide a more straightforward and unambiguous approach for broadly determining δOR-mediated responses for the purposes of the experiments completed here.

At the µOR, it has recently been demonstrated that a reduction in G-protein efficacy is responsible for lessened adverse side effect profiles, rather than a lack of β-arrestin recruitment (Gillis et al., 2020). In the GloSensor cAMP assay, 7-hydroxyspeciogynine and 7-hydroxypaynantheine act as partial agonists at δOR and *in vivo* they reduce alcohol use. This begs the question whether partial agonism rather than full agonism is driving the δOR mediated effects on alcohol intake. The δOR agonist TAN-67 efficaciously reduces alcohol use in the two-bottle choice paradigm, and is a full agonist in the cAMP assay (Chiang et al., 2016) and the [^35^S]GTPγS assay (Quock et al., 1997). However, a more recent [^35^S]GTPγS study has suggested TAN-67 may be a partial agonist (Stanczyk et al., 2019), and thus the answer for now is not clear as to whether partial agonism and/or weak β-arrestin recruitment drives reduced alcohol use by δOR agonists.

Given that agonist-bound structures of both the μOR and δOR are available (Huang et al., 2015; Claff et al., 2019), it may be possible to identify strategies by which to enhance 7-hydroxyspeciogynine affinity selectively at δOR and not μOR. Additionally, *in vivo* characterization of 7-hydroxyspeciogynine for pharmacokinetic parameters including half-life and metabolism (e.g. role of CYP3A4 and CYP2D6) will be insightful. Further behavioral analysis, including modulation of respiratory depression and anxiety-like behavior (van Rijn et al., 2010; Ko et al., 2021) would establish 7-hydroxyspeciogynine’s potential as clinical lead compound. Similarly, assessing off-target effects in a panel screen could identify other targets, including serotonin receptors (León et al., 2021) that contribute to 7-hydroxyspeciogynine’s modulation of alcohol intake.

In summary, our current and past pharmacological characterization of kratom analogs suggest that alkaloids with sub-micromolar δOR potency, micromolar potency at the μOR, and G-protein bias provide the strongest opportunity to reduce alcohol use in mice with limited side effects. We discovered 7-hydroxyspeciogynine as a novel kratom-derived analog that decreases alcohol intake by activating δORs *in vitro* and *in vivo* but with limited μOR *in vivo* agonist activity, leading to a broadened therapeutic window as evident from a lack of rewarding, locomotive, and seizurogenic effects and a MTD of at least 10 mg∙kg^−1^. Our findings support the utility of targeting the δOR to reduce volitional alcohol consumption, and further demonstrate the effectiveness of using the kratom alkaloids as lead scaffolds for developing G-protein–biased δOR agonists for treatment of AUD.

## Data Availability

The original contributions presented in the study are included in the article/Supplementary Materials, further inquiries can be directed to the corresponding author/s.

## Abbreviations

CPA: conditioned place aversion; CPP: conditioned place preference; DAMGO: (2S)-2-[[2-[[(2R)-2-[[(2S)-2-Amino-3-(4-hydroxyphenyl)propanoyl]amino]propanoyl] amino]acetyl]-methylamino]-N-(2-hydroxyethyl)-3-phenylpropanamide; MTD: maximum tolerated dose; δOR: delta opioid receptor; κOR: kappa opioid receptor; µOR: mu opioid receptor; U50,488: 2-(3,4-dichlorophenyl)-N-methyl-N-[(1R,2R)-2-pyrrolidin-1-ylcyclohexyl]acetamide.
