# Supplementary material for: Evaluation of Kratom Opioid Derivatives as Potential Treatment Option for Alcohol Use Disorder
Source: Front Pharmacol. 2021 Nov 3;12:764885. doi: 10.3389/fphar.2021.764885 (PMC8596301; doi:10.3389/fphar.2021.764885)
Supplement: Supplementary file 1 [file DataSheet1.PDF]

Supplementary Material

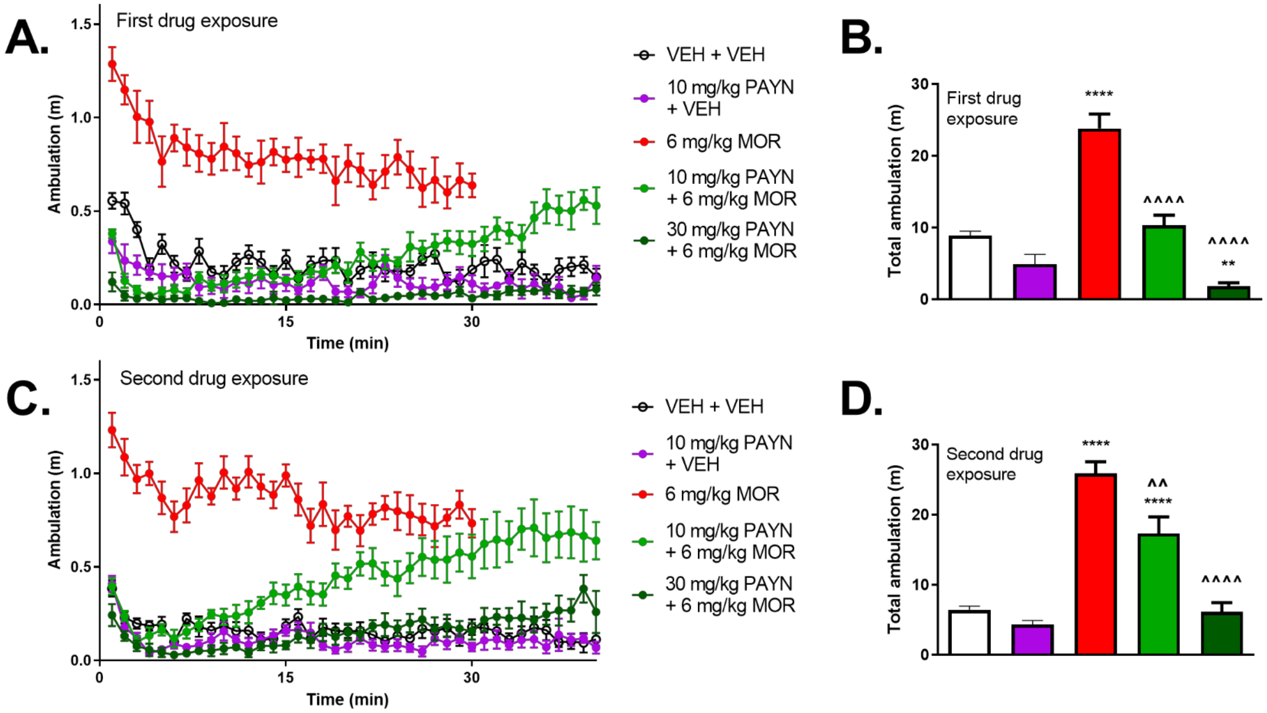

Supplemental Figure 1. Paynantheine locomotor effects across two drug exposures. Locomotor data was extracted from the conditioning sessions of the CPP experiments shown in Figure 2 A and G (data from both drug exposures is averaged in Figure 2) and is shown as ambulation over time **(A** and **C)** and total ambulation **(B** and **D)**. For comparison, locomotor data for morphine (6 mg·kg^{-1} morphine) was extracted from a previous CPP experiment with 30-minute conditioning sessions. The vehicle locomotor data was extracted from the non-drug paired side conditioning session for 10 mg·kg^{-1} paynantheine + vehicle group. For locomotor data, statistical significance was obtained by a one-way ANOVA with Dunnett's multiple comparisons to VEH + VEH (stars), and as a one-way ANOVA between the treatments including morphine with Dunnett's multiple comparisons to MOR (carets). ** p<0.01, ^^^^ or **** p<0.0001 (for details see Supplemental Table 2.)

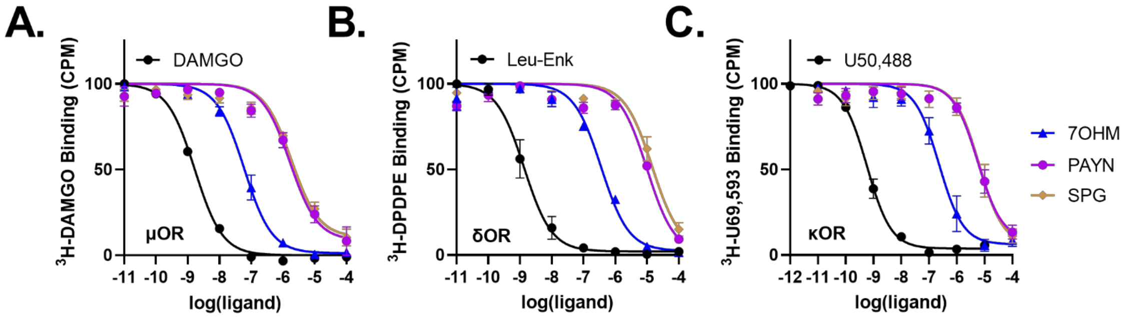

Supplemental Figure 2. Kratom alkaloid binding to opioid receptors. Kratom alkaloids 7-hydroxymitragynine (7OHM), paynantheine (PAYN), and speciogynine (SPG) were characterized for binding affinity to μOR, δOR, and κOR in competitive radioligand binding assays with [^{3}H]DAMGO, [^{3}H]DPDPE, [^{3}H]U69,593, respectively (**A, B, C**). Curves for the kratom opioids are representative of the averaged values from a minimum of 3 independent assays.

Supplemental Table 1. Kratom opioids characterization summary							
**Compounds**	**Binding**		***cAMP***			***β-arrestin 2***	
**μOR**	**pK_i**	**K_i (μM)**	**pIC_{50}**	**IC_{50} (μM)**	**α**	**pEC_{50}**	**α**
DAMGO	9.6 ± 0.1 (1)	0.00024	8.0 ± 0.1 (6)	0.0099	100	6.6 ± 0.1 (6)	100
7-OH MITRA	7.7 ± 0.1 (6)	0.019	7.8 ± 0.1 (5)	0.016	84 ± 3	ND (3)	ND
SPG	6.2 ± 0.1 (5)	0.59	5.5 ± 0.1 (5)	4.21	87 ± 6	ND (3)	ND
PAYN	6.3 ± 0.1 (4)	0.52	5.4 ± 0.1 (5)	4.08	100 ± 0	ND (3)	ND
**δOR**	**pK_i**	**K_i (μM)**	**pIC_{50}**	**IC_{50} (μM)**	**α**	**pEC_{50}**	**α**
Leu-Enk	9.2 ± 0.1 (3)	0.00070	8.4 ± 0.1 (9)	0.0042	100	7.4 ± 0.1 (7)	100
7-OH MITRA	6.7 ± 0.1 (4)	0.19	5.7 ± 0.2 (8)	0.96	80 ± 8	6.4 ± 0.3 (6)	14 ± 1
SPG	5.1 ± 0.1 (6)	5.34	5.0 ± 0.3 (5)	12.4	94 ± 4	ND (3)	ND
PAYN	5.3 ± 0.1 (5)	7.82	5.6 ± 0.2 (4)	3.55	64 ± 13	ND (3)	ND
**κOR**	**pK_i**	**K_i (μM)**	**pIC_{50}**	**IC_{50} (μM)**	**α**	**pEC_{50}**	**α**
U50,488	10.0 ± 0.2 (2)	0.000099	8.5 ± 0.1 (5)	0.0034	100	7.1 ± 0.1 (6)	100
7-OH MITRA	6.9 ± 0.1 (4)	0.14	6.2 ± 0.3 (9)	1.04	77 ± 5	ND (4)	ND
SPG	5.4 ± 0.1 (5)	3.0	4.7 ± 0.3 (5)	6.55	70 ± 20	ND (4)	ND
PAYN	5.5 ± 0.1 (5)	4.0	5.3 ± 0.2 (4)	7.43	95 ± 5	ND (6)	ND

Data for 7-hydroxymitragynine, speciogynine, and paynantheine in the GloSensor cAMP assay and the β-arrestin2 recruitment assay was generated in a previous publication (Gutridge et al. 2020) and is shown here for comparison to the kratom analogs.

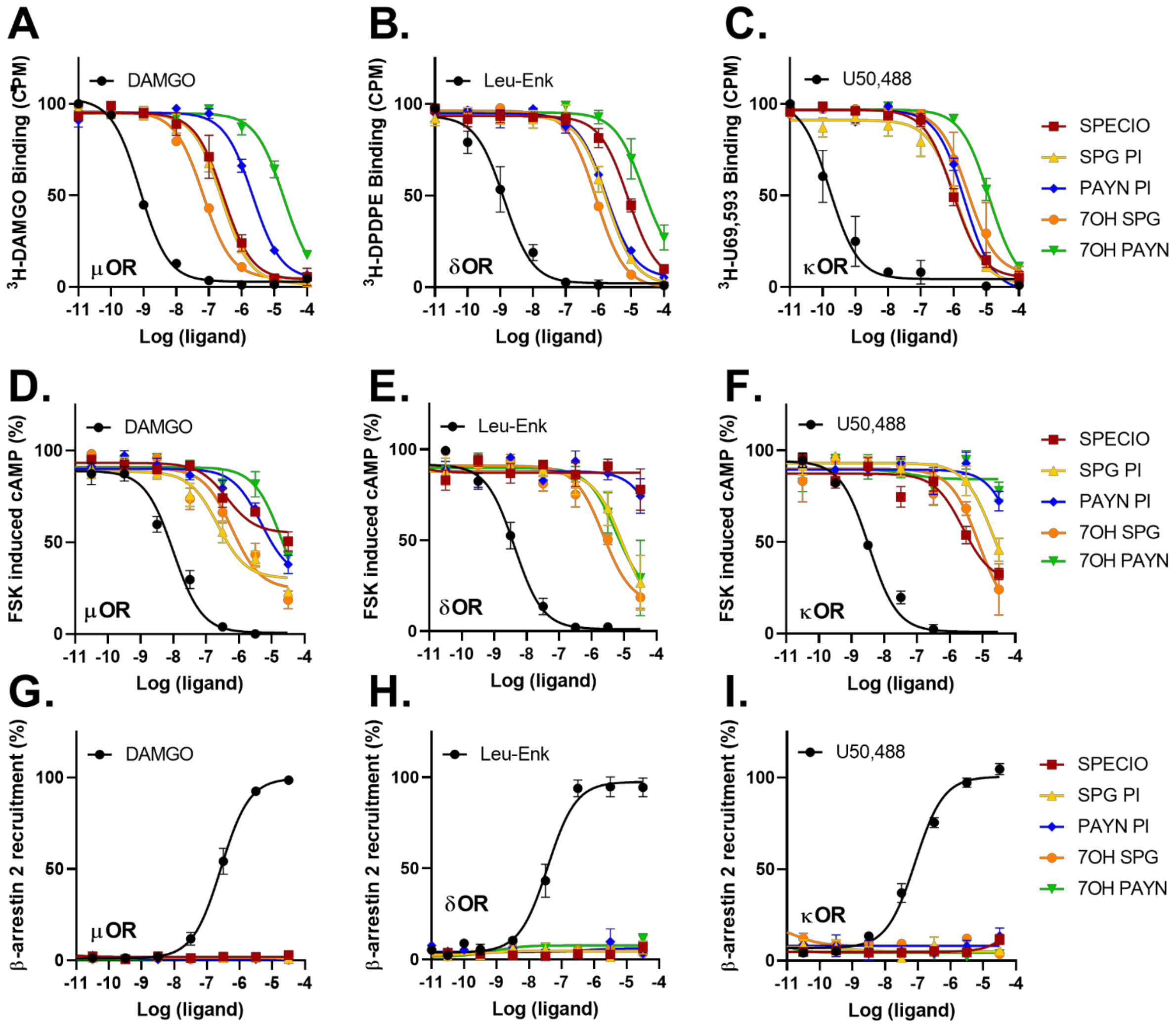

Supplemental Figure 3. Pharmacological characterization of kratom analogs at opioid receptors. Kratom alkaloid derivatives speciociliatine (SPECIO), speciogynine pseudo indoxyl (SPG PI), paynantheine pseudo indoxyl (PAYN PI), 7-hydroxy speciogynine (7OH SPG), and 7-hydroxy paynantheine (7OH PAYN) were characterized for binding affinity using [^{3}H]DAMGO, [^{3}H]DPDPE, [^{3}H]U69,593 (**A, B, C**), inhibition of forskolin-induced cAMP in a Glo-sensor assay in transfected HEK-293 cells (**D, E, F)** and the ability of the alkaloids to recruit β-arrestin 2 in a PathHunter assay. (**G, H, I**) at μOR (**A, D, G**), δOR (**B, E, H**), and κOR (**C, F, I**). All curves are representative of the averaged values from a minimum of 3 independent assays.

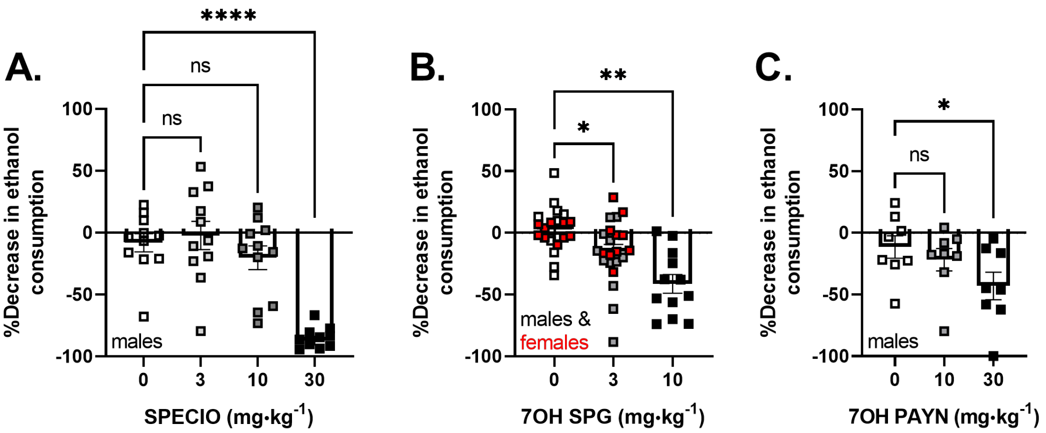

Supplemental Figure 4. Percent decrease in ethanol consumption by kratom analogs. 2-bottle choice ethanol consumption data from Figure 4 is revisualized as percent decreases in 10% ethanol consumption following treatment with **(A)** speciociliatine (3, 10, and 30 mg·kg^{-1}, i.p., n=11 WT male), **(B)** 7-hydroxyspeciogynine (3 and 10 mg·kg^{-1}, s.c., n=12 WT male, n=9 WT female), or **(C)** 7-hydroxypaynantheine (10 and 30 mg·kg^{-1}, s.c., n=8 WT male). * p<0.05, ** p<0.01, **** p<0.0001 (for details see Supplemental Table 6.)

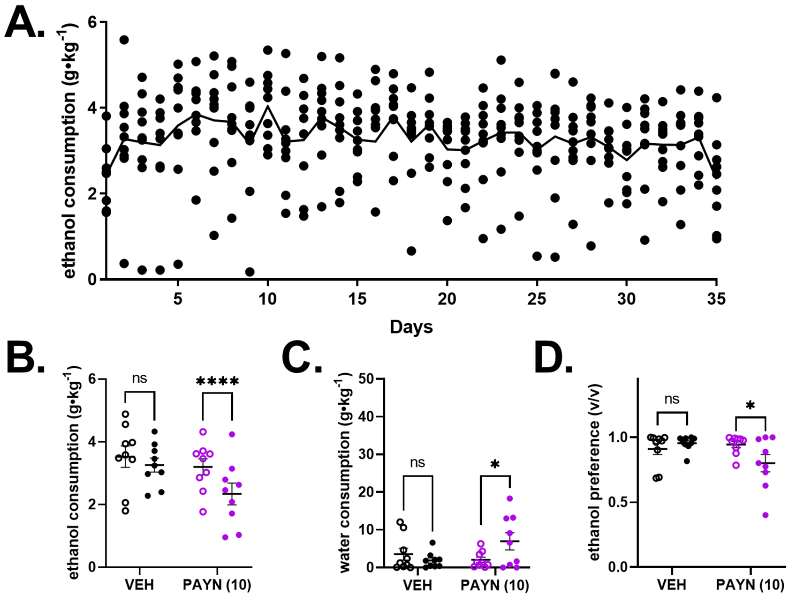

Supplemental Figure 5. Ethanol consumption timeline and full data set of δOR knockout mice 2-bottle choice experiment. (A) Consistent ethanol consumption across seven weeks for male δOR KO mice in a 2-bottle choice experiment used to measure δOR contributions of 7-hydroxyspeciogynine, 7-hydroxypaynantheine, and paynantheine (paynantheine data not shown in main text). Effect of 10 mg·kg^{-1} paynantheine in δOR KO mice on ethanol modulation **(B)**, water consumption **(C)**, and ethanol preference **(D)**. * p<0.05, **** p<0.0001 (for details see Supplemental Table 6-8.)

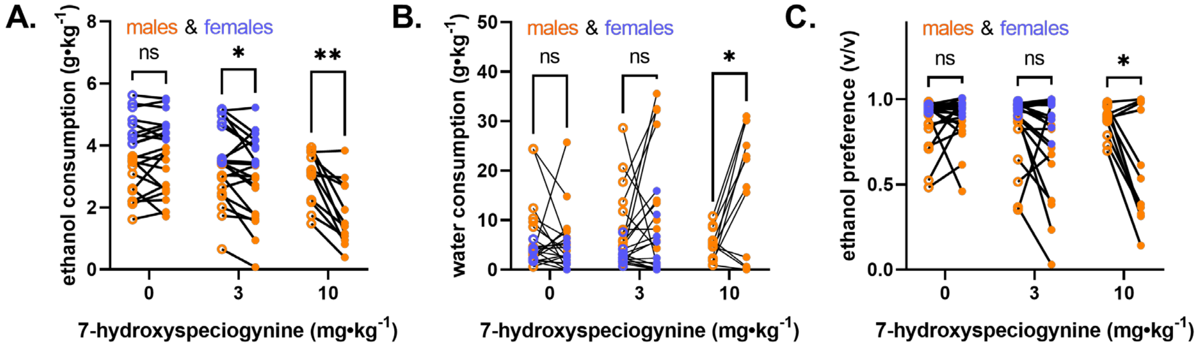

Supplemental Figure 6. Raw data for male and female mice treated with 7-hydroxyspeciogynine in the 2-bottle choice paradigm. Pooled data for 10% ethanol consumption, water consumption and ethanol preference **(A-C**, respectively**)** in wildtype male (n=12) and female mice (n = 10) in a voluntary two-bottle choice, limited access, drinking-in-the-dark paradigm, following treatment with 7-hydroxyspeciogynine (3, and 10 mg·kg^{-1}, s.c.)
Male and female mice are depicted with orange and blue symbols, respectively. Open circles are the average intake/preference on the preceding three days (baseline), and closed circles are the intake on Fridays following drug exposure. Statistical analyses summarized in Supplemental Tables 6-8. Thresholds for statistical significance: *p<0.05, ** p<0.01.

Supplemental Table 2. Behavioral statistics for locomotor and motor incoordination effects

Drugs Tested	Statistical Test	T or F (df) value	P value	Significance	Post-hoc test	P-value	Significance
(Figure 1B)<br>3 mg/kg 7OHM<br>1 mg/kg NLX + 3 mg/kg 7OHM	Unpaired, t-test (two-tailed)	T=5.441, df=8	0.0006	***	n/a		
(Figure 2G)<br>VEH + VEH<br>10 mg/kg PAYN + VEH<br>6 mg/kg MOR<br>10 mg/kg PAYN + 6 mg/kg MOR<br>30 mg/kg PAYN + 6 mg/kg MOR	1-way ANOVA (pulled from CPP data)	F (4, 29) = 50.66	<0.0001	****	Dunnett's Multiple Comparisons (vs VEH + VEH)	 --<br>0.2026<br><0.0001<br>0.0044<br>0.1694	 <br>ns<br>****<br>**<br>ns
(Figure 2G)<br>6 mg/kg MOR<br>10 mg/kg PAYN + 6 mg/kg MOR<br>30 mg/kg PAYN + 6 mg/kg MOR	1-way ANOVA (pulled from CPP data)	F (2, 15) = 39.25	<0.0001	****	Dunnett's Multiple Comparisons (vs MOR)	 --<br>0.0004<br><0.0001	 <br>***<br>****
(Figure 4G) WT mice<br>30 mg/kg Speciociliatine at:<br>5 min<br>15 min<br>30 min<br>60 min<br>120 min	One-sample t-test (two-tailed) vs hypothetical mean of 100	 <br>t=3.478, df=7<br>t=5.809, df=7<br>t=5.344, df=7<br>t=2.994, df=7<br>t=1.953, df=7	 <br>0.0103<br>0.0007<br>0.0011<br>0.0201<br>0.0918	 <br>*<br>***<br>**<br>*<br>ns	n/a		
(Figure 4G) δOR KO mice<br>30 mg/kg Speciociliatine at:<br>5 min<br>15 min<br>30 min<br>60 min<br>120 min<br>150 min	One-sample t-test (two-tailed) vs hypothetical mean of 100	 <br>t=2.282, df=6<br>t=4.396, df=6<br>t=4.827, df=6<br>t=3.407, df=6<br>t=3.264, df=6<br>t=1.240, df=6	 <br>0.0626<br>0.0046<br>0.0029<br>0.0144<br>0.0172<br>0.2612	 <br>ns<br>**<br>**<br>*<br>*<br>ns	n/a		
(Figure 4G) WT vs δOR KO mice, 30 mg/kg Speciociliatine:<br>Timepoint<br>Genotype<br>Timepoint x Genotype	Mixed-effects model (REML) with Geisser-Greenhouse correction	 <br>F (2.234, 15.64) = 22.03<br>F (1.000, 7.000) = 0.3393<br>F (1.941, 11.26) = 1.930	 <br>< 0.0001<br>0.5785<br>0.1906	 <br>****<br>ns<br>ns			
(Figure 7B)<br>VEH<br>10 mg/kg 7OH SPG	Paired t-test (two-tailed) (pulled from CPP data)	T=0.7552, df=6	0.4787	ns	n/a		

Supplemental Table 2. Behavioral statistics for locomotor & motor incoordination effects, continued							
**Drugs Tested**	**Statistical Test**	**T or F (df) value**	**P value**	**Significance**	**Post-hoc test**	**P-value**	**Significance**
(Supplemental Figure 1B, first drug exposure)<br>VEH + VEH<br>10 mg/kg PAYN + VEH<br>6 mg/kg MOR<br>10 mg/kg PAYN + 6 mg/kg MOR<br>30 mg/kg PAYN + 6 mg/kg MOR	1-way ANOVA (pulled from CPP data)	F (4, 28) = 38.45	<0.0001	****	Dunnett's Multiple Comparisons (vs VEH + VEH)	--<br>0.1197<br><0.0001<br>0.8671<br>0.0051	ns<br>****<br>ns<br>**
(Supplemental Figure 1B, first drug exposure)<br>6 mg/kg MOR<br>10 mg/kg PAYN + 6 mg/kg MOR<br>30 mg/kg PAYN + 6 mg/kg MOR	1-way ANOVA (pulled from CPP data)	F (2, 15) = 46.67	<0.0001	****	Dunnett's Multiple Comparisons (vs MOR)	--<br><0.0001<br><0.0001	****<br>****
(Supplemental Figure 1D, second drug exposure)<br>VEH + VEH<br>10 mg/kg PAYN + VEH<br>6 mg/kg MOR<br>10 mg/kg PAYN + 6 mg/kg MOR<br>30 mg/kg PAYN + 6 mg/kg MOR	1-way ANOVA (pulled from CPP data)	F (4, 29) = 48.45	<0.0001	****	Dunnett's Multiple Comparisons (vs VEH + VEH)	--<br>0.6162<br><0.0001<br><0.0001<br>0.9998	ns<br>****<br>****<br>ns
(Supplemental Figure 1D, second drug exposure)<br>6 mg/kg MOR<br>10 mg/kg PAYN + 6 mg/kg MOR<br>30 mg/kg PAYN + 6 mg/kg MOR	1-way ANOVA (pulled from CPP data)	F (2, 15) = 26.31	<0.0001	****	Dunnett's Multiple Comparisons (vs MOR)	--<br>0.0090<br><0.0001	**<br>****

Supplemental Table 3. Behavioral statistics for seizure-like effects							
**Drugs Tested**	**Statistical Test**	**T (df) value**	**P value**	**Significance**	**Post-hoc test**	**P-value**	**Significance**
(Figure 2C, Area under the curve)<br>δOR KO, 30 mg/kg paynantheine<br>WT, 30 mg/kg paynantheine	Unpaired t-test (two-tailed) with Welch's correction	t=0.9205, df=6.738	0.3891	ns	n/a		

Supplemental Table 4. Behavioral statistics for conditioned place preference effects							
**Drugs Tested**	**Statistical Test**	**T (df) value**	**P value**	**Significance**	**Post-hoc test**	**P-value**	**Significance**
(Figure 2A, brief CPP)<br>Pre-test, 10 mg/kg PAYN + VEH<br>Post-test, 10 mg/kg PAYN + VEH	Paired t-test (two-tailed)	t=2.606, df=7	0.0351	*	n/a		
(Figure 2B, extended CPP)<br>Pre-test, 10 mg/kg PAYN<br>Post-test, 10 mg/kg PAYN	Paired t-test (two-tailed)	t=2.227, df=7	0.0612	ns	n/a		
(Figure 2E, brief CPP)<br>Pre-test, 10 mg/kg PAYN + MOR<br>Post-test, 10 mg/kg PAYN + MOR	Paired t-test (two-tailed)	t=3.214, df=7	0.0148	*	n/a		
(Figure 2E, brief CPP)<br>Pre-test, 30 mg/kg PAYN + MOR<br>Post-test, 30 mg/kg PAYN + MOR	Paired t-test (two-tailed)	t=6.609, df=5	0.0012	**	n/a		
(Figure 7A, extended CPP)<br>Pre-test, 10 mg/kg 7OH SPG<br>Post-test, 10 mg/kg 7OH SPG	Paired t-test (two-tailed)	t=1.592, df=7	0.1554	ns	n/a		

Supplemental Table 5. Behavioral statistics for antinociceptive effects							
**Drugs Tested**	**Statistical Test**	**T or F (df) value**	**P value**	**Significance**	**Post-hoc test**	**P-value**	**Significance**
(Figure 2D)<br>VEH<br>10 mg/kg PAYN	Paired t-test (two-tailed)	t=0.7256, df=9	0.4865	ns	n/a		
(Figure 2D)<br>VEH<br>30 mg/kg PAYN	Paired t-test (two-tailed)	t=2.925, df=9	0.0169	*	n/a		
(Figure 2D)<br>6 mg/kg MOR<br>10 mg/kg PAYN + 6 mg/kg MOR<br>30 mg/kg PAYN + 6 mg/kg MOR	Repeated Measures 1-way ANOVA	F (1.943, 17.49) = 12.38	0.0005	***	Dunnett's Multiple Comparisons (to 6 mg/kg MOR)	--<br>0.6330<br>0.0019	ns<br>**
(Figure 7D)<br>VEH<br>10 mg/kg 7OH SPG	Paired t-test (two-tailed)	T=0.6193, df=9	0.5511	ns	n/a		
(Figure 7D)<br>6 mg/kg MOR<br>10 mg/kg 7OH SPG + 6 mg/kg MOR	Unpaired t-test with Welch's correction	T=0.2660<br>df=5.994	0.7991	ns	n/a		

Supplemental Table 6. Behavioral statistics for ethanol consumption in Figures 4-6 and Supplemental Figure 4-6							
**Drugs Tested**	**Statistical Test**	**F (df) value**	**P value**	**Significance**	**Post-hoc test**	**P-value**	**Significance**
Fig. 4A<br>SPECIO<br>WT males	Repeated Measures 2-way ANOVA<br>Dose:<br>Time:<br>Dose x Time:	 F (3, 30) = 36.48<br>F (1, 10) = 50.17<br>F (3, 30) = 13.30	 <0.0001<br><0.0001<br><0.0001	 ****<br>****<br>****	Sidak's MC (T-R vs F)<br>VEH<br>3 mg/kg<br>10 mg/kg<br>30 mg/kg	 0.9615<br>0.9911<br>0.0994<br><0.0001	 ns<br>ns<br>ns<br>****
Fig. 4D<br>SPECIO<br>δOR KO males	Repeated Measures 2-way ANOVA<br>Dose:<br>Time:<br>Dose x Time:	 F (1, 9) = 25.36<br>F (1, 9) = 61.69<br>F (1, 9) = 83.26	 0.0007<br><0.0001<br><0.0001	 ***<br>****<br>****	Sidak's MC (T-R vs F)<br>VEH<br>30 mg/kg	 0.9681<br><0.0001	 ns<br>****
Fig. 5A<br>7-OH SPG<br>WT males	Repeated Measures 2-way ANOVA<br>Dose:<br>Time:<br>Dose x Time:	 F (2, 22) = 6.973<br>F (1, 11) = 22.58<br>F (2, 22) = 8.675	 0.0045<br>0.0006<br>0.0017	 **<br>***<br>**	Sidak's MC (T-R vs F)<br>VEH<br>3 mg/kg<br>10 mg/kg	 0.9957<br>0.0802<br><0.0001	 ns<br>ns<br>****
Fig. 5D<br>7-OH PAYN<br>WT males	Repeated Measures 2-way ANOVA<br>Dose:<br>Time:<br>Dose x Time:	 F (2, 14) = 4.200<br>F (1, 7) = 13.79<br>F (2, 14) = 5.515	 0.0373<br>0.0075<br>0.0171	 *<br>**<br>*	Sidak's MC (T-R vs F)<br>VEH<br>10 mg/kg<br>30 mg/kg	 0.3102<br>0.0219<br><0.0001	 ns<br>*<br>****
Fig. 5G<br>(multiple drugs)<br>δOR KO males	Repeated Measures 2-way ANOVA<br>Dose:<br>Time:<br>Dose x Time:	 F (4, 32) = 6.407<br>F (1, 8) = 16.46<br>F (4, 32) = 1.851	 0.0007<br>0.0036<br>0.1435	 ***<br>**<br>ns	Sidak's MC (T-R vs F)<br>VEH<br>3 mg/kg 7-OH SPG<br>10 mg/kg 7-OH SPG<br>30 mg/kg 7-OH PAYN<br>10 mg/kg PAYN	 0.5103<br>0.2658<br>0.0269<br>0.1128<br><0.0001	 ns<br>ns<br>*<br>ns<br>****
Fig. 6A<br>7-OH SPG<br>WT females	Repeated Measures 2-way ANOVA<br>Dose:<br>Time:<br>Dose x Time:	 F (1, 9) = 12.01<br>F (1, 9) = 0.9069<br>F (1, 9) = 2.196	 0.0071<br>0.3658<br>0.1725	 **<br>ns<br>ns	Sidak's MC (T-R vs F)<br>VEH<br>3 mg/kg	 0.9507<br>0.1981	 ns<br>ns
Fig. 6D<br>7-OH SPG<br>Δ ethanol<br>WT males &<br>females	Mixed-effects model (REML) with<br>Geisser-Greenhouse correction<br>Main effect: treatment	 <br>F (1.539, 40.80) = 13.36	 <br>0.0001	 <br>***	Dunnett's MC (vs<br>VEH)<br>3 mg/kg<br>10 mg/kg	 <br>0.0165<br>0.0064	 <br>*<br>**
Supp Fig. 4A<br>SPECIO<br>WT male	Repeated Measures 1-way ANOVA	F (1.607, 16.07) = 28.71	<0.0001	****	Dunnett's MC (to veh)<br>3 mg/kg<br>10 mg/kg<br>30 mg/kg	 0.9165<br>0.4171<br><0.0001	 ns<br>ns<br>****
Supp Fig. 4B<br>7-OH SPG<br>WT male &<br>female	Mixed-effects model (REML) with<br>Geisser-Greenhouse correction,<br>fixed effect: drug dose	F (1.617, 42.84) = 14.13	<0.0001	****	Dunnett's MC (to veh)<br>3 mg/kg<br>10 mg/kg	 0.0296<br>0.0041	 *<br>**
Supp Fig. 4C<br>7-OH PAYN<br>WT male	Repeated Measures 1-way ANOVA	F (1.403, 9.819) = 4.442	0.0523	ns	Dunnett's MC (to veh)<br>10 mg/kg<br>30 mg/kg	 0.6927<br>0.0148	 ns<br>*

Supplemental Table 6 Continued. Behavioral statistics for ethanol consumption in Figures 4-6 and Supplemental Figure 4-6							
Supp Fig. 6A<br>7-OH SPG<br>WT males &<br>females	Mixed-effects model (REML) with<br>Geisser-Greenhouse correction<br>Dose:<br>Time:<br>Dose x Time:	 <br>F (1.379, 28.97) = 10.33<br>F (1.000, 21.00) = 17.25<br>F (1.560, 17.16) = 7.350	 <br>0.0014<br>0.0004<br>0.0076	 <br>**<br>***<br>**	Sidak's MC (T-R vs F)<br>VEH<br>3 mg/kg<br>10 mg/kg	 0.9623<br>0.0265<br>0.0020	 ns<br>*<br>**

Supplemental Table 7. Behavioral statistics for water consumption in Figures 4-6 and Supplemental Figures 5-6							
**Drugs Tested**	**Statistical Test**	**F (df) value**	**P value**	**Significance**	**Post-hoc test**	**P-value**	**Significance**
Fig. 4B<br>SPECIO<br>WT males	Repeated Measures 2-way ANOVA<br>Dose:<br>Time:<br>Dose x Time:	 F (3, 30) = 5.262<br>F (1, 10) = 1.164<br>F (3, 30) = 3.009	 0.0049<br>0.3061<br>0.0456	 **<br>ns<br>*	Sidak's MC (T-R vs F)<br>VEH<br>3 mg/kg<br>10 mg/kg<br>30 mg/kg	 >0.9999<br>0.2007<br>0.1653<br>0.8500	 ns<br>ns<br>ns<br>ns
Fig 4E<br>SPECIO<br>δOR KO<br>males	Repeated Measures 2-way ANOVA<br>Dose:<br>Time:<br>Dose x Time:	 F (1, 9) = 2.859<br>F (1, 9) = 2.703<br>F (1, 9) = 0.07905	 0.1251<br>0.1346<br>0.7849	 ns<br>ns<br>ns	Sidak's MC (T-R vs F)<br>VEH<br>30 mg/kg	 0.1766<br>0.0949	 ns<br>ns
Fig. 5B<br>7-OH SPG<br>WT males	Repeated Measures 2-way ANOVA<br>Dose:<br>Time:<br>Dose x Time:	 F (2, 22) = 8.706<br>F (1, 11) = 4.161<br>F (2, 22) = 3.489	 0.0016<br>0.0661<br>0.0483	 **<br>ns<br>*	Sidak's MC (T-R vs F)<br>VEH<br>3 mg/kg<br>10 mg/kg	 0.9478<br>0.3955<br>0.0112	 ns<br>ns<br>*
Fig. 5E<br>7-OH PAYN<br>WT males	Repeated Measures 2-way ANOVA<br>Dose:<br>Time:<br>Dose x Time:	 F (2, 14) = 4.129<br>F (1, 7) = 4.920<br>F (2, 14) = 4.149	 0.0389<br>0.0621<br>0.0385	 *<br>ns<br>*	Sidak's MC (T-R vs F)<br>VEH<br>10 mg/kg<br>30 mg/kg	 0.8304<br>0.5975<br>0.0015	 ns<br>ns<br>**
Fig. 5H<br>(multiple<br>drugs)<br>δOR KO<br>males	Repeated Measures 2-way ANOVA<br>Dose:<br>Time:<br>Dose x Time:	 F (4, 32) = 0.9791<br>F (1, 8) = 0.8157<br>F (4, 32) = 2.188	 0.4327<br>0.3928<br>0.0927	 ns<br>ns<br>ns	Sidak's MC (T-R vs F)<br>VEH<br>3 mg/kg 7-OH SPG<br>10 mg/kg 7-OH SPG<br>30 mg/kg 7-OH PAYN<br>10 mg/kg PAYN	 0.8781<br>0.9597<br>0.9946<br>0.9916<br>0.0369	 ns<br>ns<br>ns<br>ns<br>*
Fig. 6B<br>7-OH SPG<br>WT females	Repeated Measures 2-way ANOVA<br>Dose:<br>Time:<br>Dose x Time:	 F (1, 9) = 0.5787<br>F (1, 9) = 0.3734<br>F (1, 9) = 2.021	 0.4663<br>0.5562<br>0.1889	 ns<br>ns<br>ns	Sidak's MC (T-R vs F)<br>VEH<br>3 mg/kg	 0.8402<br>0.3216	 ns<br>ns
Fig. 6E<br>7-OH SPG<br>Δ water<br>WT males &<br>females	Mixed-effects model (REML) with<br>Geisser-Greenhouse correction<br>Main effect: treatment	 <br>F (1.733, 27.74) = 5.978	 <br>0.0091	 <br>**	Dunnett's MC (vs<br>VEH)<br>3 mg/kg<br>10 mg/kg	 <br>0.1804<br>0.0342	 <br>ns<br>*
Supp Fig. 6B<br>7-OH SPG<br>WT males &<br>females	Mixed-effects model (REML) with<br>Geisser-Greenhouse correction<br>Dose:<br>Time:<br>Dose x Time:	 <br>F (1.812, 38.05) = 3.378<br>F (1.000, 21.00) = 4.518<br>F (1.761, 19.37) = 7.674	 <br>0.0490<br>0.0456<br>0.0046	 <br>*<br>*<br>**	Sidak's MC (T-R vs F)<br>VEH<br>3 mg/kg<br>10 mg/kg	 0.8483<br>0.4627<br>0.0190	 ns<br>ns<br>*

Supplemental Table 8. Behavioral statistics for ethanol preference in Figures 4-6 and Supplemental Figures 5-6							
**Drugs Tested**	**Statistical Test**	**F (df) value**	**P value**	**Significance**	**Post-hoc test**	**P-value**	**Significance**
Fig. 4C<br>SPECIO<br>WT males	Repeated Measures 2-way ANOVA<br>Dose:<br>Time:<br>Dose x Time:	 F (3, 30) = 24.20<br>F (1, 10) = 17.10<br>F (3, 30) = 7.521	 <0.0001<br>0.0020<br>0.0007	 ****<br>**<br>***	Sidak's MC (T-R vs F)<br>VEH<br>3 mg/kg<br>10 mg/kg<br>30 mg/kg	 0.9872<br>0.9428<br>0.0980<br><0.0001	 ns<br>ns<br>ns<br>****
Fig. 4F<br>SPECIO<br>δOR KO<br>males	Repeated Measures 2-way ANOVA<br>Dose:<br>Time:<br>Dose x Time:	 F (1, 9) = 32.58<br>F (1, 9) = 23.26<br>F (1, 9) = 64.72	 0.0003<br>0.0009<br><0.0001	 ***<br>***<br>****	Sidak's MC (T-R vs F)<br>VEH<br>30 mg/kg	 0.6980<br><0.0001	 ns<br>****
Fig. 5C<br>7-OH SPG<br>WT males	Repeated Measures 2-way ANOVA<br>Dose:<br>Time:<br>Dose x Time:	 F (2, 22) = 9.997<br>F (1, 11) = 8.284<br>F (2, 22) = 4.140	 0.0008<br>0.0150<br>0.0298	 ***<br>*<br>*	Sidak's MC (T-R vs F)<br>VEH<br>3 mg/kg<br>10 mg/kg	 0.9804<br>0.3480<br>0.0036	 ns<br>ns<br>**
Fig. 5F<br>7-OH PAYN<br>WT males	Repeated Measures 2-way ANOVA<br>Dose:<br>Time:<br>Dose x Time:	 F (2, 14) = 3.845<br>F (1, 7) = 5.193<br>F (2, 14) = 3.980	 0.0467<br>0.0567<br>0.0428	 *<br>ns<br>*	Sidak's MC (T-R vs F)<br>VEH<br>10 mg/kg<br>30 mg/kg	 0.7024<br>0.3805<br>0.0010	 ns<br>ns<br>**
Fig. 5I<br>(multiple drugs)<br>δOR KO<br>males	Repeated Measures 2-way ANOVA<br>Dose:<br>Time:<br>Dose x Time:	 F (4, 32) = 0.9705<br>F (1, 8) = 1.630<br>F (4, 32) = 2.313	 0.4372<br>0.2375<br>0.0789	 ns<br>ns<br>ns	Sidak's MC (T-R vs F)<br>VEH<br>3 mg/kg 7-OH SPG<br>10 mg/kg 7-OH SPG<br>30 mg/kg 7-OH PAYN<br>10 mg/kg PAYN	 0.9077<br>0.8891<br>0.9971<br>0.9849<br>0.0296	 ns<br>ns<br>ns<br>ns<br>*
Fig. 6C<br>7-OH SPG<br>WT females	Repeated Measures 2-way ANOVA<br>Dose:<br>Time:<br>Dose x Time:	 F (1, 9) = 1.006<br>F (1, 9) = 0.2648<br>F (1, 9) = 2.053	 0.3420<br>0.6193<br>0.1857	 ns<br>ns<br>ns	Sidak's MC (T-R vs F)<br>VEH<br>3 mg/kg	 0.8094<br>0.3399	 ns<br>ns
Fig. 6F<br>7-OH SPG<br>Δ ethanol pref<br>WT males & females	Mixed-effects model (REML) with Geisser-Greenhouse correction<br>Main effect: treatment	F (1.645, 43.58) = 7.889	0.0022	**	Dunnett's MC (vs VEH)<br>3 mg/kg<br>10 mg/kg	 0.1644<br>0.0255	 ns<br>*
Supp Fig. 6C<br>7-OH SPG<br>WT males & females	Mixed-effects model (REML) with Geisser-Greenhouse correction<br>Dose:<br>Time:<br>Dose x Time:	 F (1.496, 31.41) = 4.542<br>F (1.000, 21.00) = 15.69<br>F (1.667, 18.34) = 10.43	 0.0273<br>0.0007<br>0.0015	 *<br>***<br>**	Sidak's MC (T-R vs F)<br>VEH<br>3 mg/kg<br>10 mg/kg	 0.9059<br>0.3682<br>0.0117	 ns<br>ns<br>*

Supplemental Table 9. Comparing effect of sex on ethanol consumption, water consumption, and ethanol preference in Figures 5 & 6							
**Drugs Tested**	**Statistical Test**	**F (df) value**	**P value**	**Significance**	**Post-hoc test**	**P-value**	**Significance**
Fig. 5 & 6<br>Baseline ethanol consumption,<br>WT male vs female	Repeated Measures 2-way ANOVA<br>Sex<br>Time<br>Sex x Time	 F (1, 20) = 39.05<br>F (1, 20) = 6.295<br>F (1, 20) = 0.1027	 <0.0001<br>0.0208<br>0.7520	 ****<br>*<br>ns	Sidak's MC (M vs F)<br>VEH baseline<br>3 mg/kg baseline	 <0.0001<br><0.0001	 ****<br>****
Fig. 5 & 6<br>Baseline water consumption,<br>WT male vs female	Repeated Measures 2-way ANOVA<br>Sex<br>Time<br>Sex x Time	 F (1, 20) = 7.511<br>F (1, 20) = 0.1950<br>F (1, 20) = 0.5752	 0.0126<br>0.6636<br>0.5752	 *<br>ns<br>ns	Sidak's MC (M vs F)<br>VEH baseline<br>3 mg/kg baseline	 0.0865<br>0.0164	 ns<br>*
Fig. 5 & 6<br>Baseline ethanol preference,<br>WT male vs female	Repeated Measures 2-way ANOVA<br>Sex<br>Time<br>Sex x Time	 F (1, 20) = 7.734<br>F (1, 20) = 1.017<br>F (1, 20) = 1.311	 0.0115<br>0.3252<br>0.2657	 *<br>ns<br>ns	Sidak's MC (M vs F)<br>VEH baseline<br>3 mg/kg baseline	 0.0690<br>0.0091	 ns<br>**
Fig. 6<br>Δ ethanol consumption following 3 mg/kg 7OHSPG,<br>WT male vs female	Repeated Measures 2-way ANOVA<br>Sex<br>Dose<br>Sex x Dose	 F (1, 20) = 0.1974<br>F (1, 20) = 7.758<br>F (1, 20) = 0.2487	 0.6616<br>0.0114<br>0.6234	 ns<br>*<br>ns	Sidak's MC (M vs F)<br>Δ ethanol (VEH)<br>Δ ethanol (3 mg/kg)	 0.9993<br>0.7635	 ns<br>ns
Fig. 6<br>Δ water consumption following 3 mg/kg 7OHSPG,<br>WT male vs female	Repeated Measures 2-way ANOVA<br>Sex<br>Dose<br>Sex x Dose	 F (1, 20) = 0.1365<br>F (1, 20) = 2.613<br>F (1, 20) = 0.6681	 0.7156<br>0.1217<br>0.4233	 ns<br>ns<br>ns	Sidak's MC (M vs F)<br>Δ water (VEH)<br>Δ water (3 mg/kg)	 0.9647<br>0.6687	 ns<br>ns
Fig. 6<br>Δ ethanol preference following 3 mg/kg 7OHSPG,<br>WT male vs female	Repeated Measures 2-way ANOVA<br>Sex<br>Dose<br>Sex x Dose	 F (1, 20) = 0.3695<br>F (1, 20) = 2.776<br>F (1, 20) = 1.054	 0.3695<br>0.1113<br>0.3168	 ns<br>ns<br>ns	Sidak's MC (M vs F)<br>Δ ethanol pref (VEH)<br>Δ ethanol pref (3 mg/kg)	 0.9765<br>0.4627	 ns<br>ns

NMR spectra of the new compounds

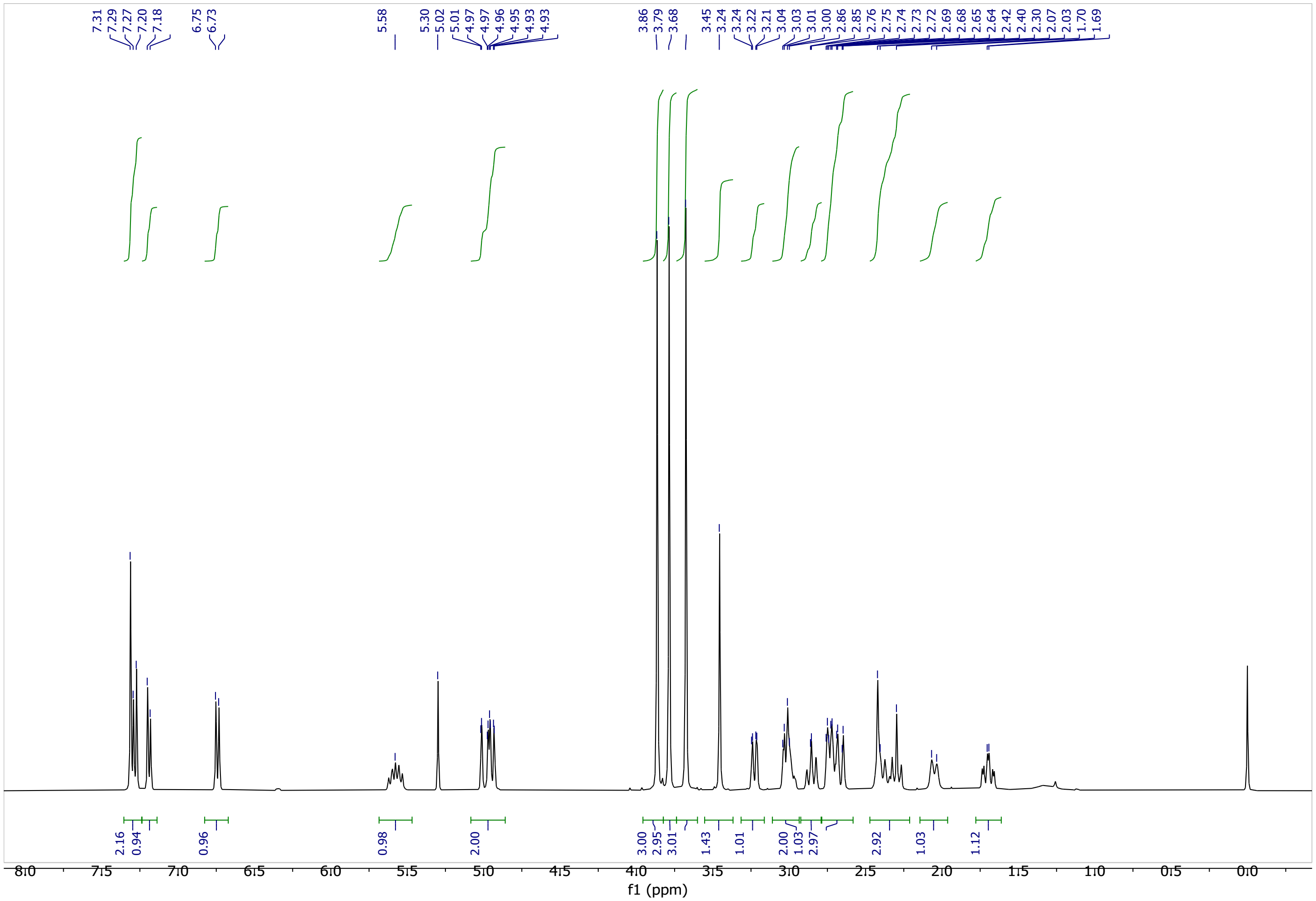

Supplemental Figure 8. [1]**H NMR of 7-hydroxypaynantheine (7OH PAYN/7) (400 MHz, $CDCl_3$)**

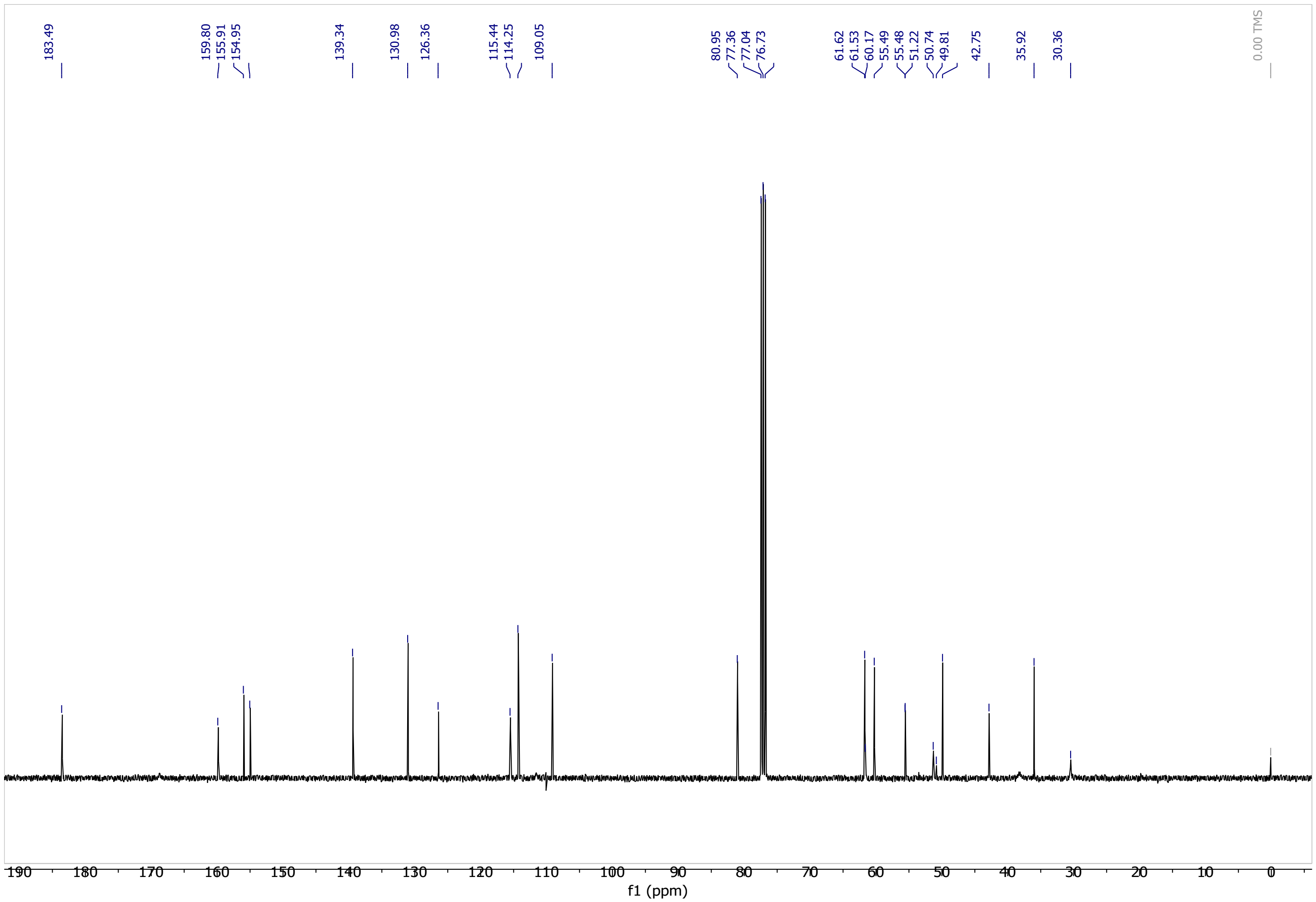

Supplemental Figure 9. ^{13}**C NMR of 7-hydroxypaynantheine (7OH PAYN/7) (100 MHz, $CDCl_3$)**

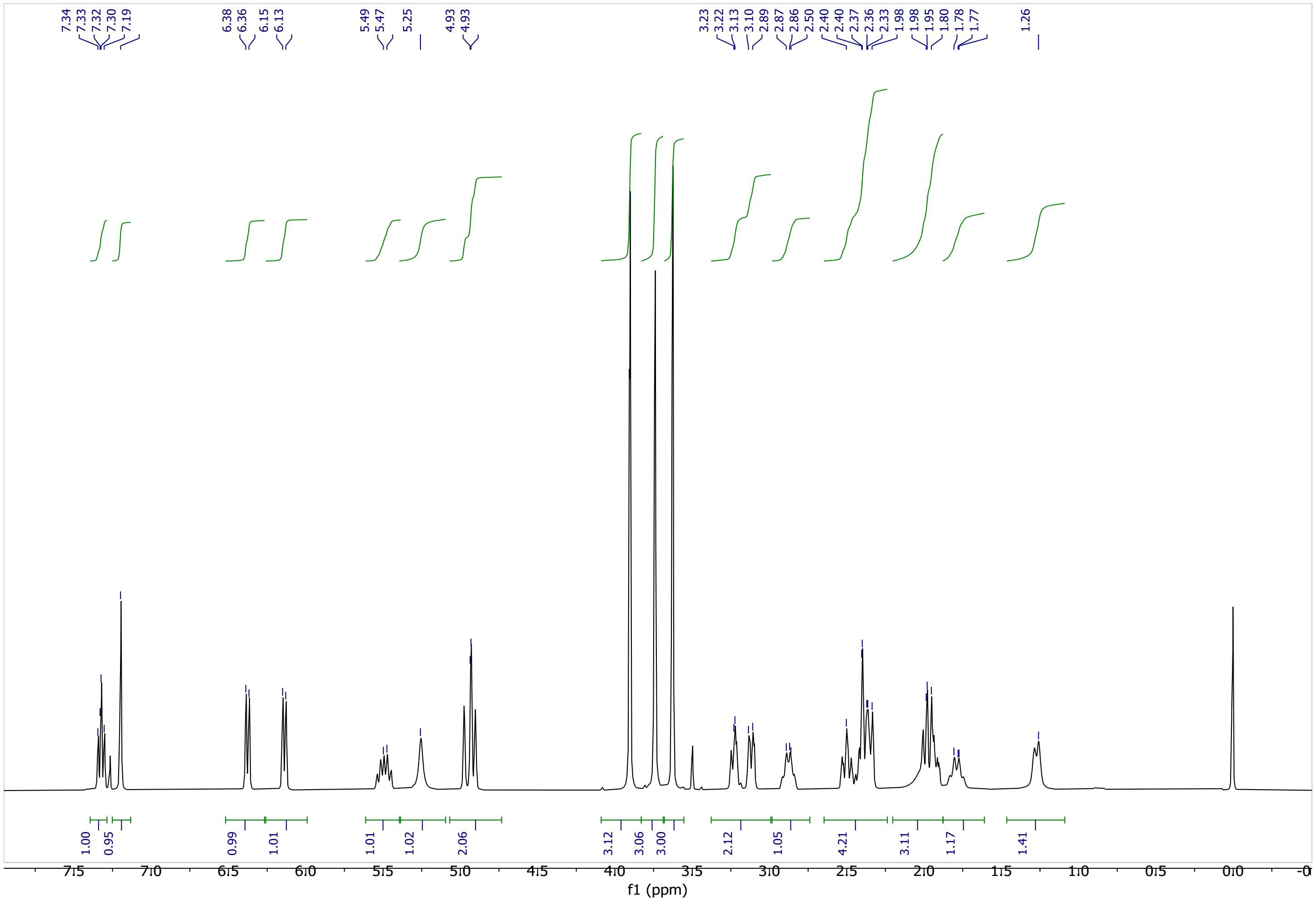

Supplemental Figure 10. ^{1}H NMR of Paynantheine pseudoindoxyl (PAYN PI/8) (500 MHz, CDCl₃)

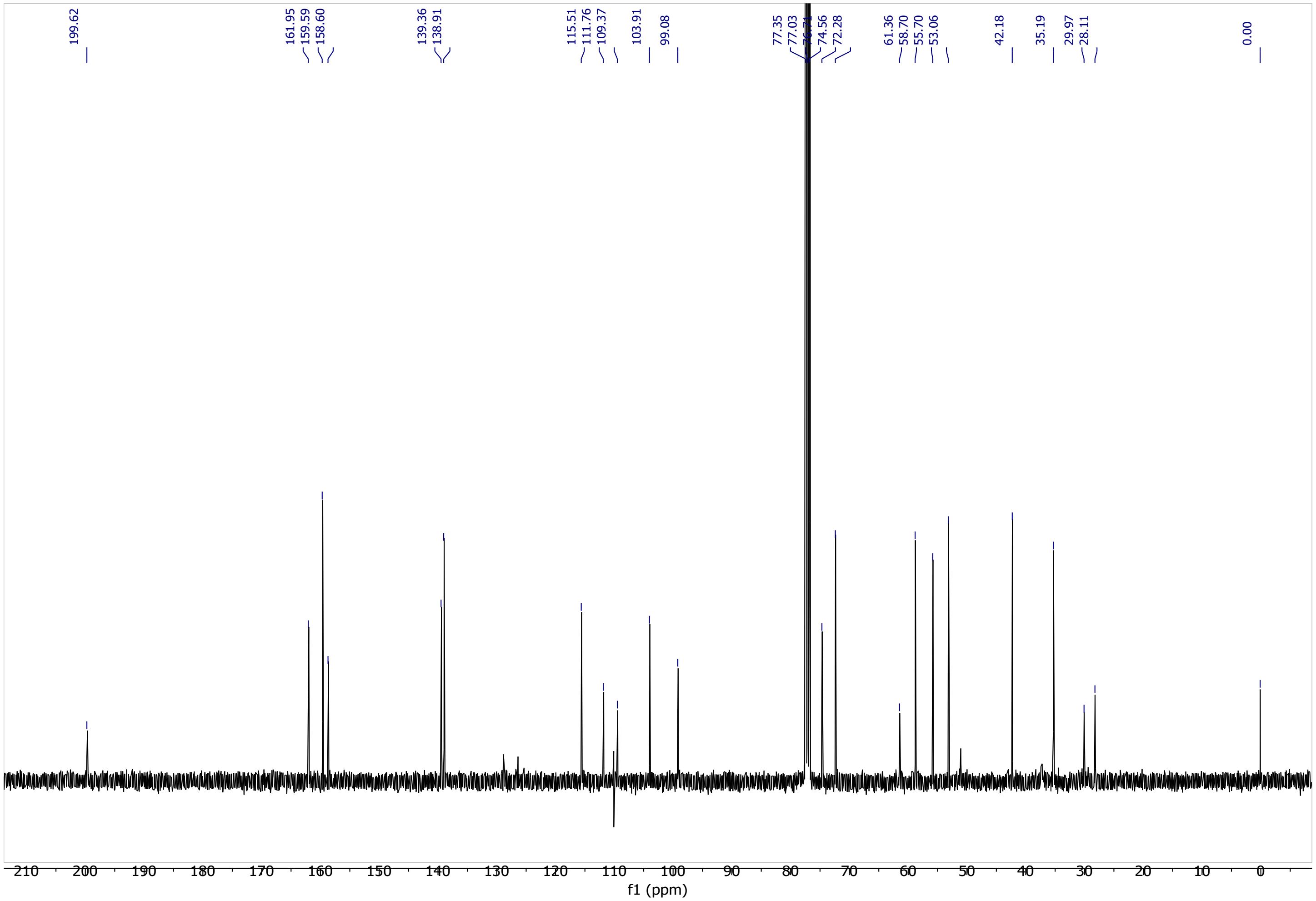

Supplemental Figure 11. ^{13}C NMR of Paynantheine pseudoindoxyl (PAYN PI/8) (100 MHz, $CDCl_3$)

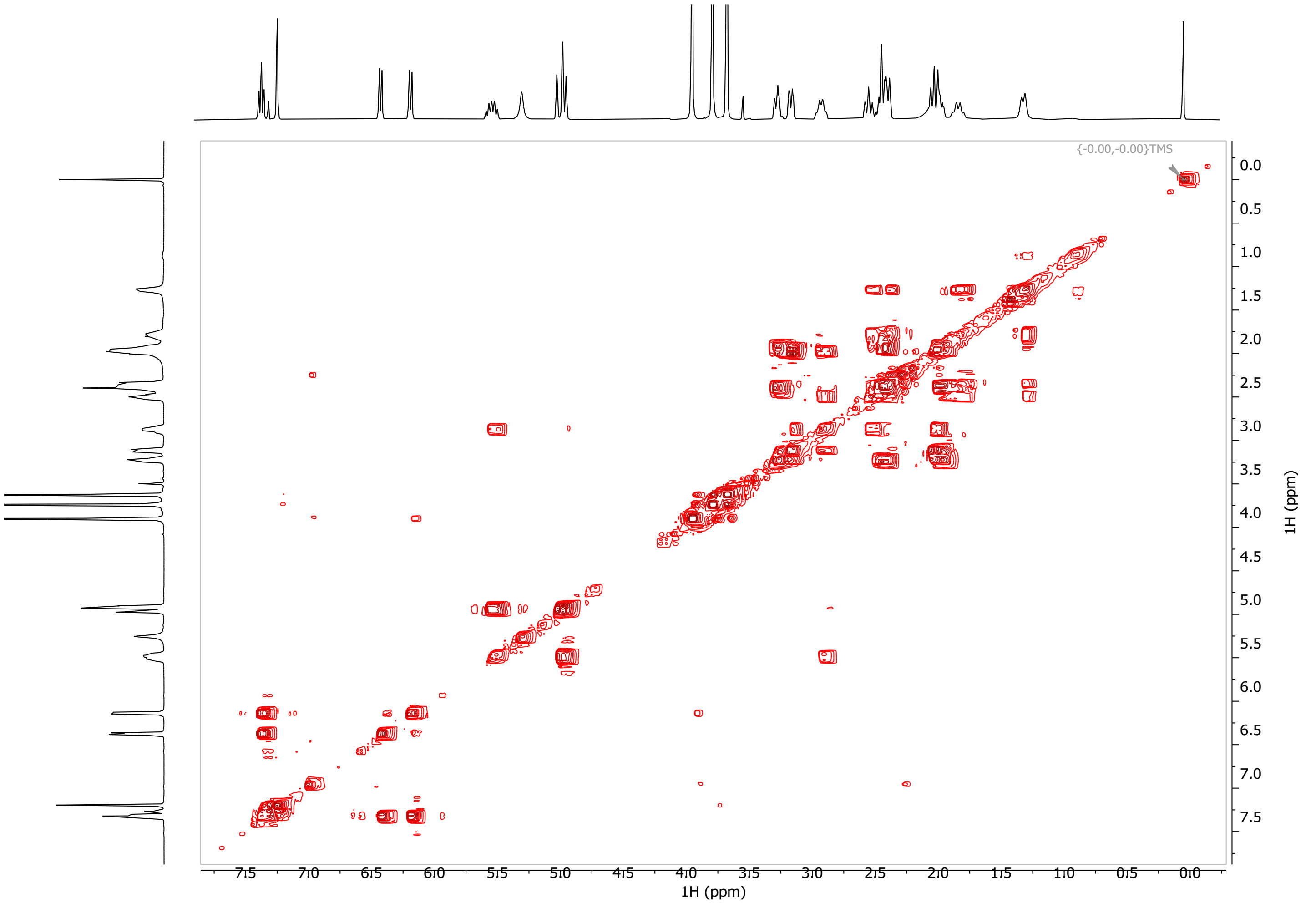

Supplemental Figure 12. COSY Spectrum of Paynantheine pseudoindoxyl (PAYN PI/8) (400 MHz, $CDCl_3$)

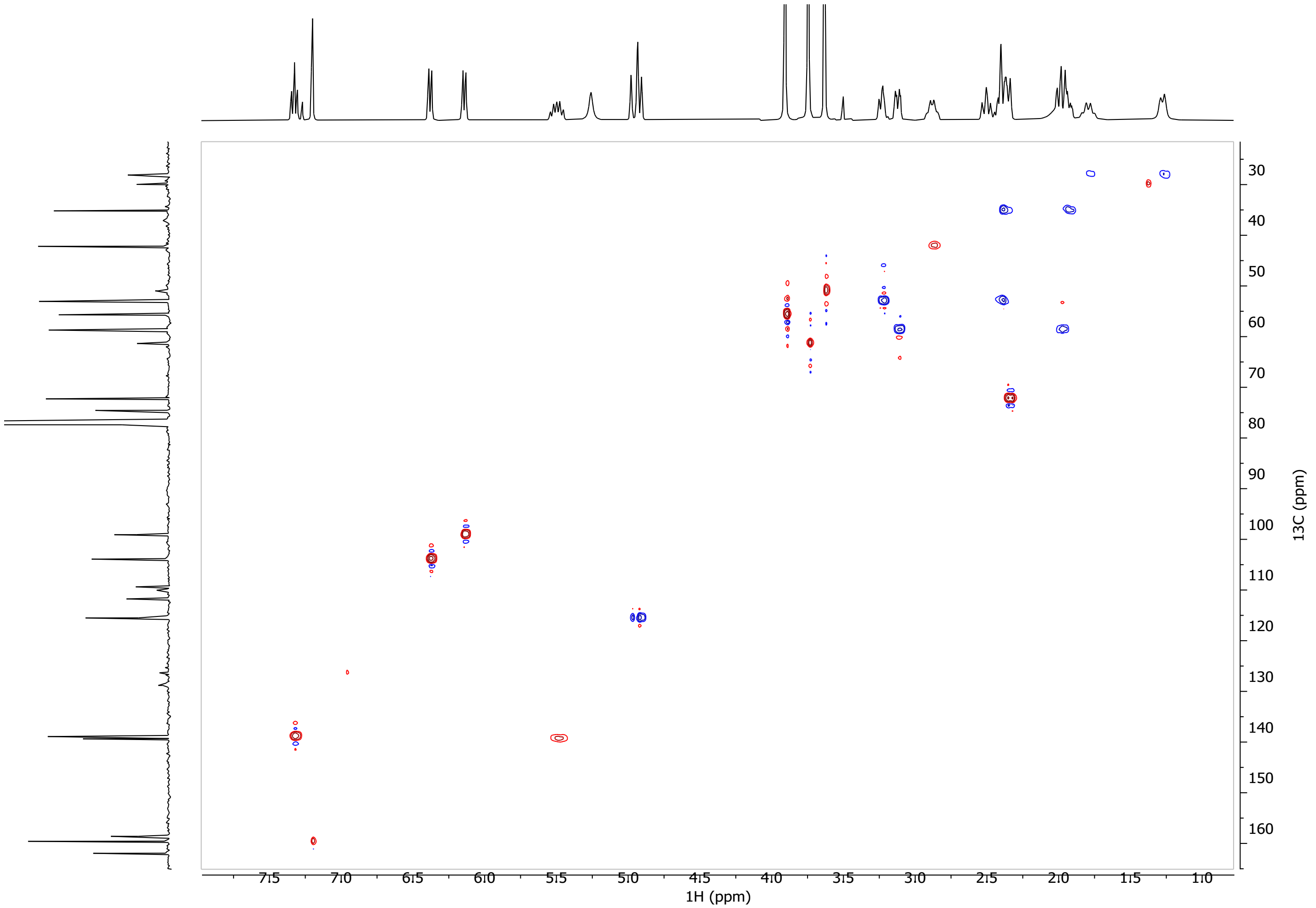

Supplemental Figure 13. HSQC Spectrum of Paynantheine pseudoindoxyl (PAYN PI/8) (400 MHz, $CDCl_3$)

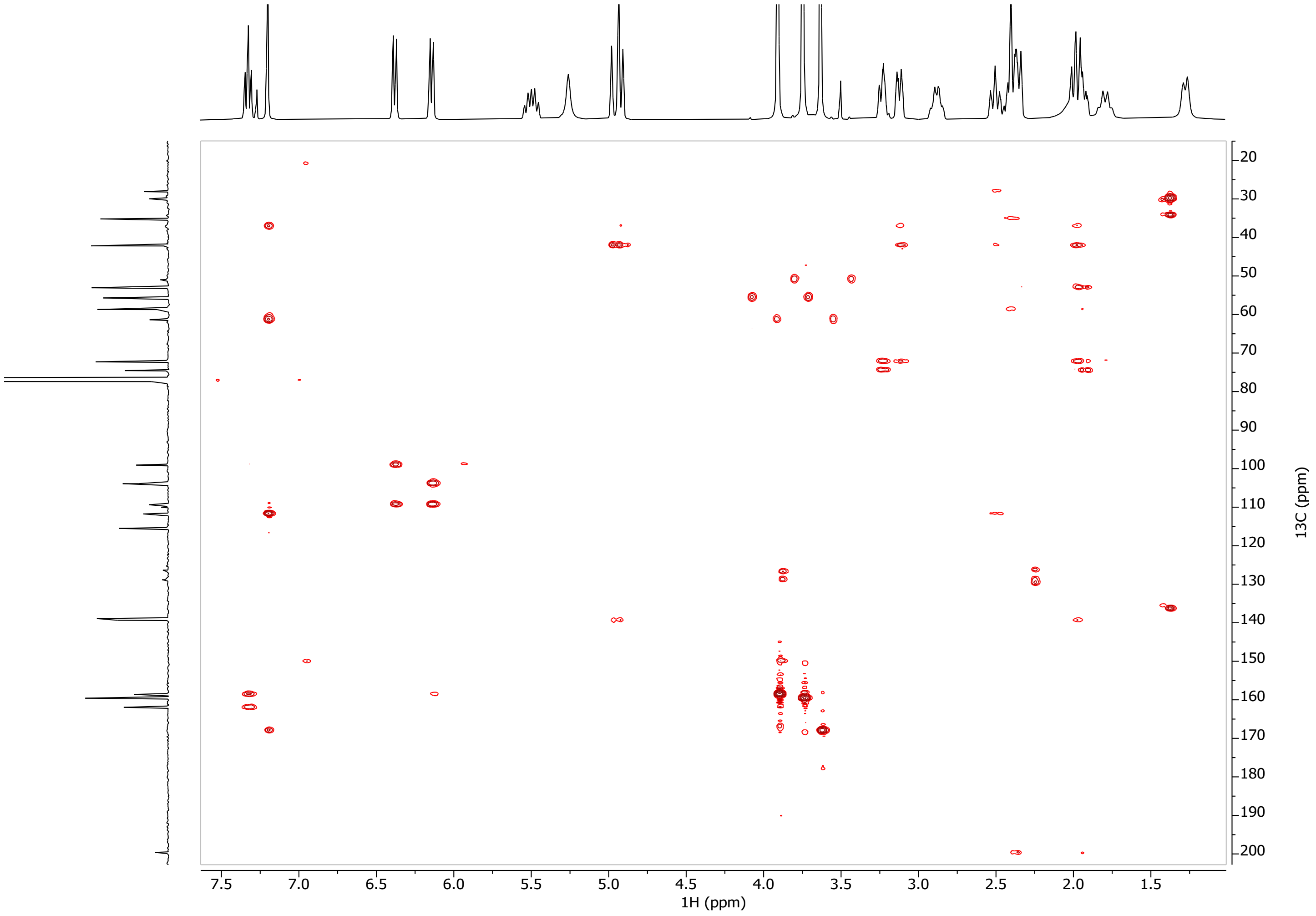

Supplemental Figure 14. HMBC Spectrum of Paynantheine pseudoindoxyl (PAYN PI/8) (400 MHz, $CDCl_3$)

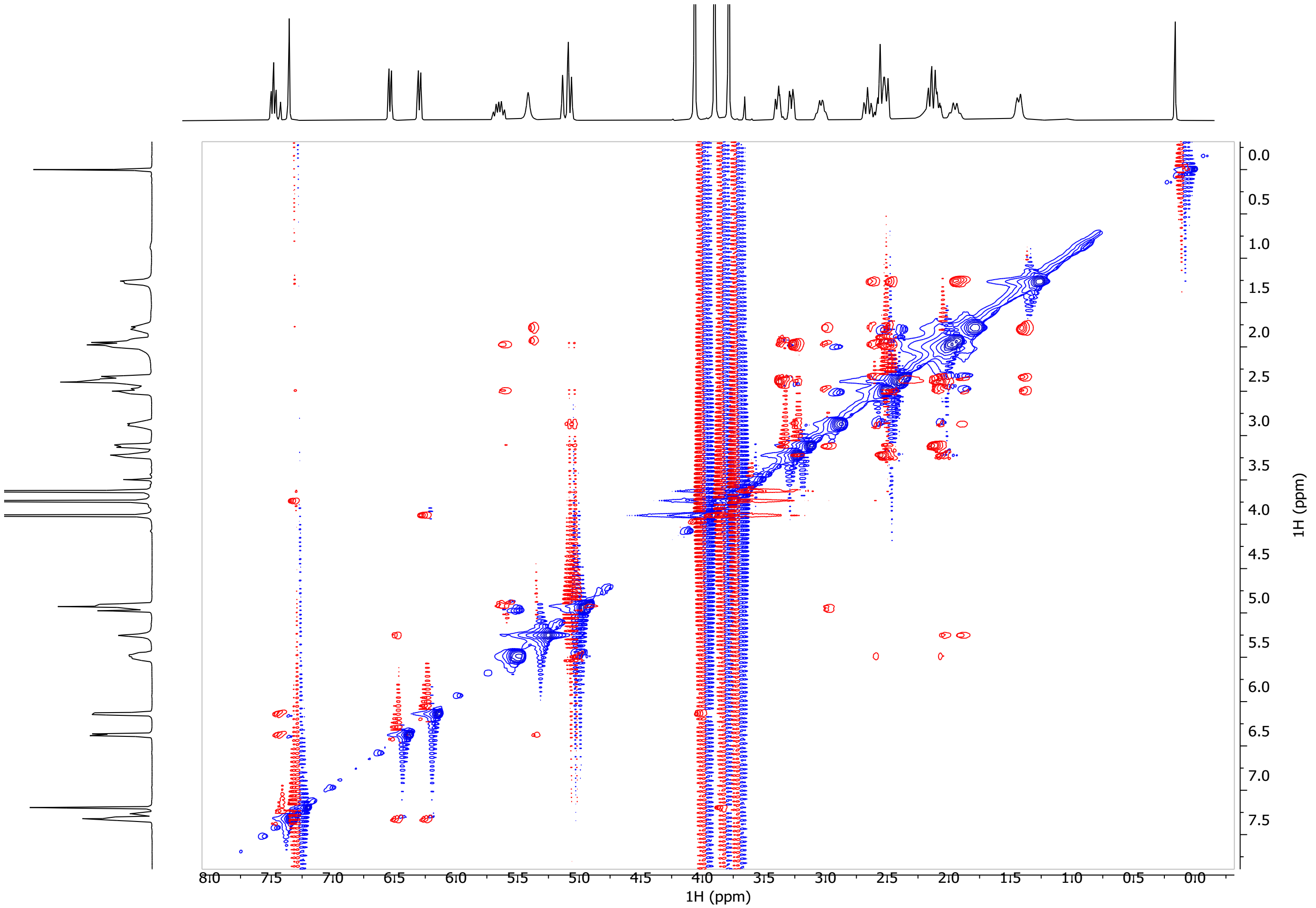

Supplemental Figure 15. NOESY Spectrum of Paynantheine pseudoindoxyl (PAYN PI/8) (400 MHz, $CDCl_3$)

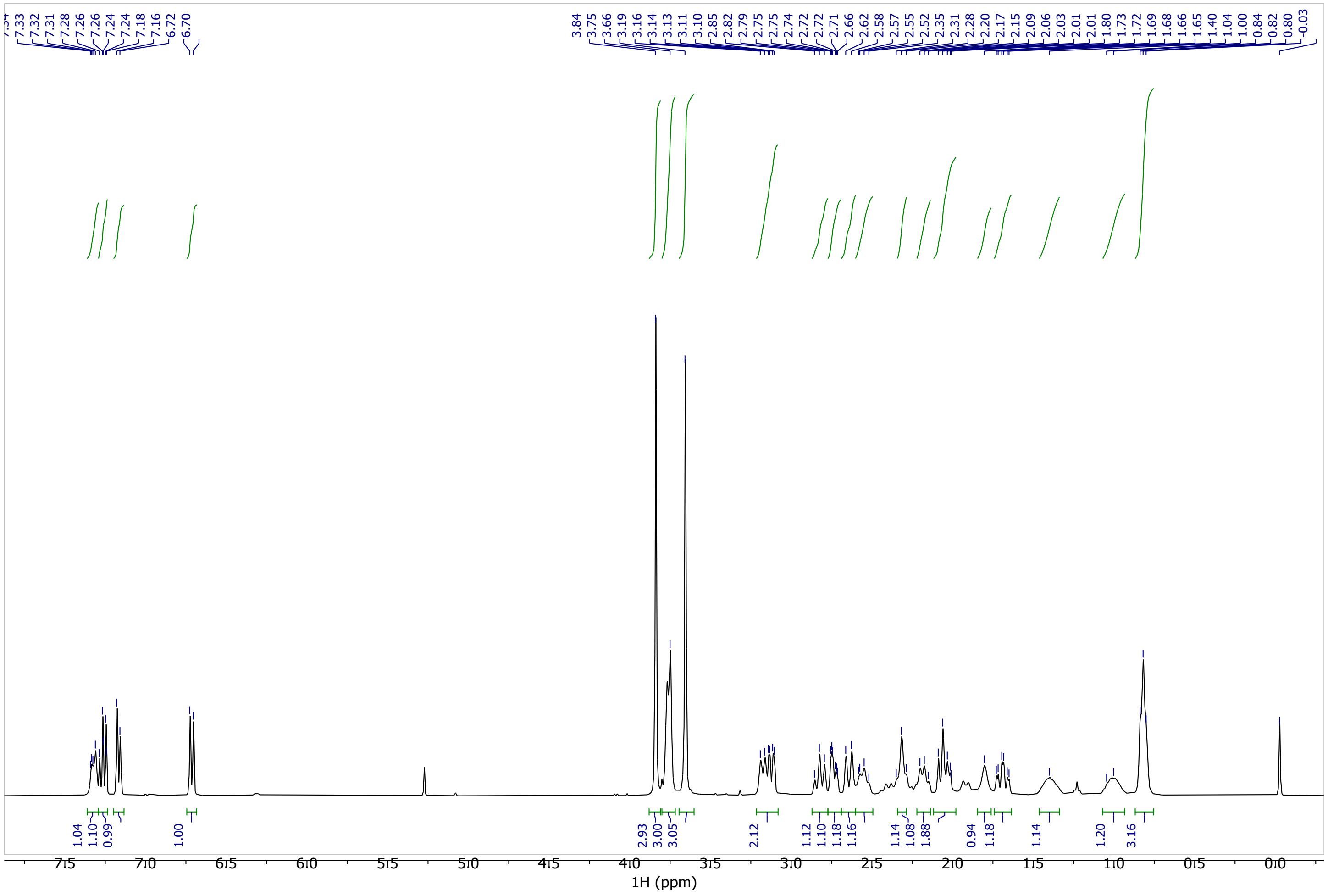

Supplemental Figure 16. ^{1}H NMR of 7-hydroxyspeciogynine (7OH SPG/9) (400 MHz, $CDCl_3$)

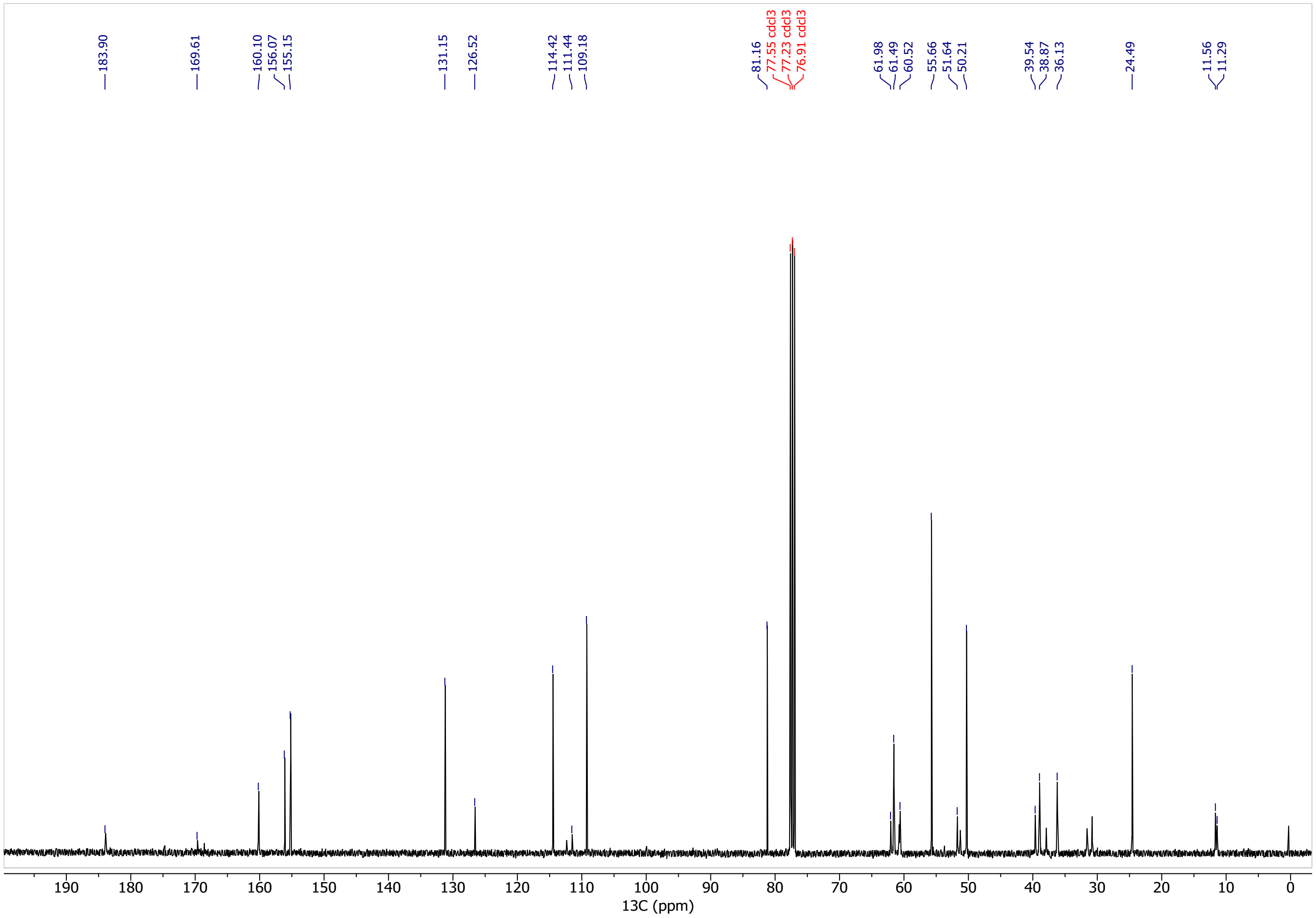

Supplemental Figure 17. ^{13}**C NMR of 7-hydroxyspeciogynine (7OH SPG/9) (100 MHz, CDCl₃)**

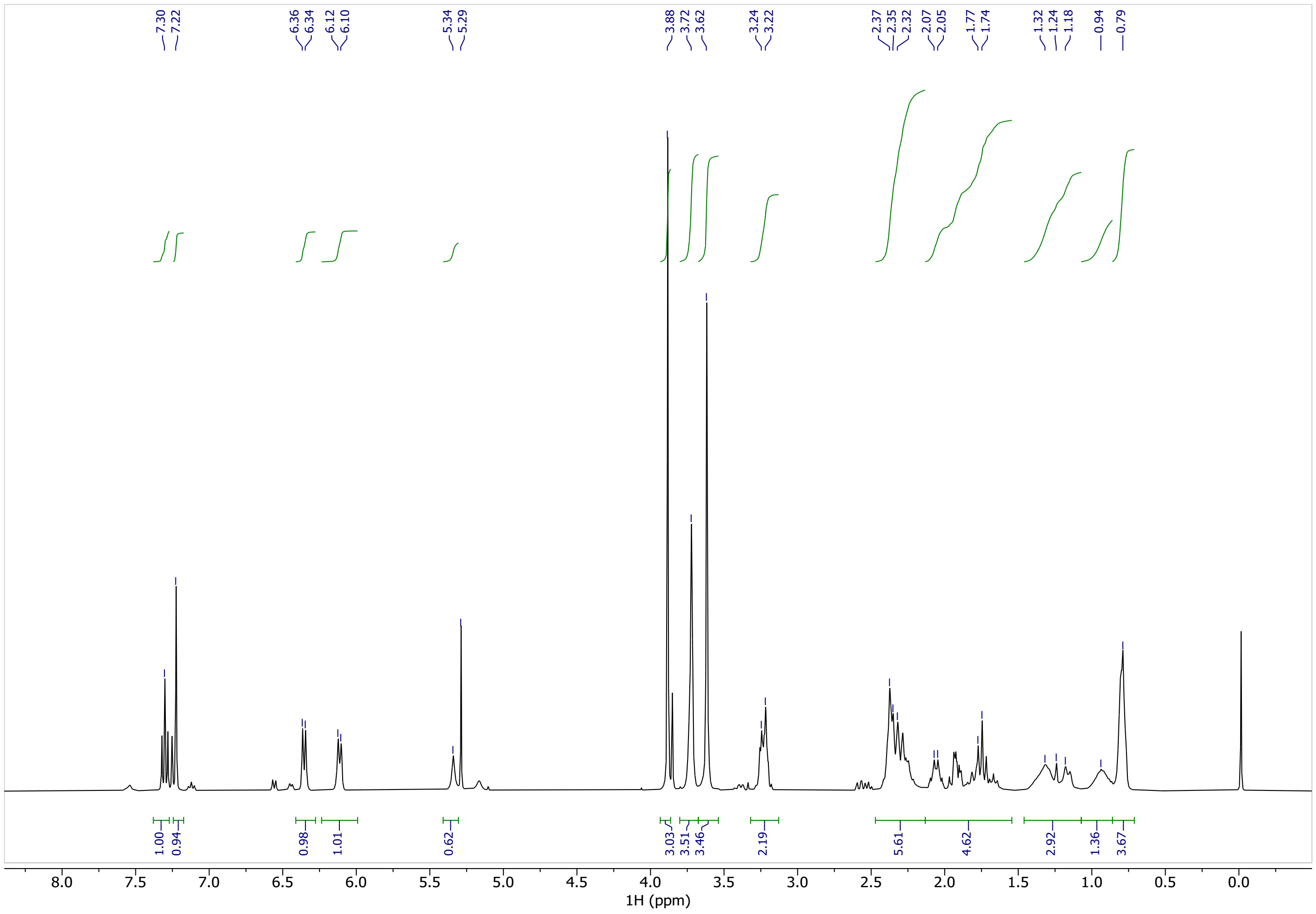

Supplemental Figure 18. ^{1}H NMR of Speciogynine pseudoindoxyl (SPG PI/10) (500 MHz, $CDCl_3$)

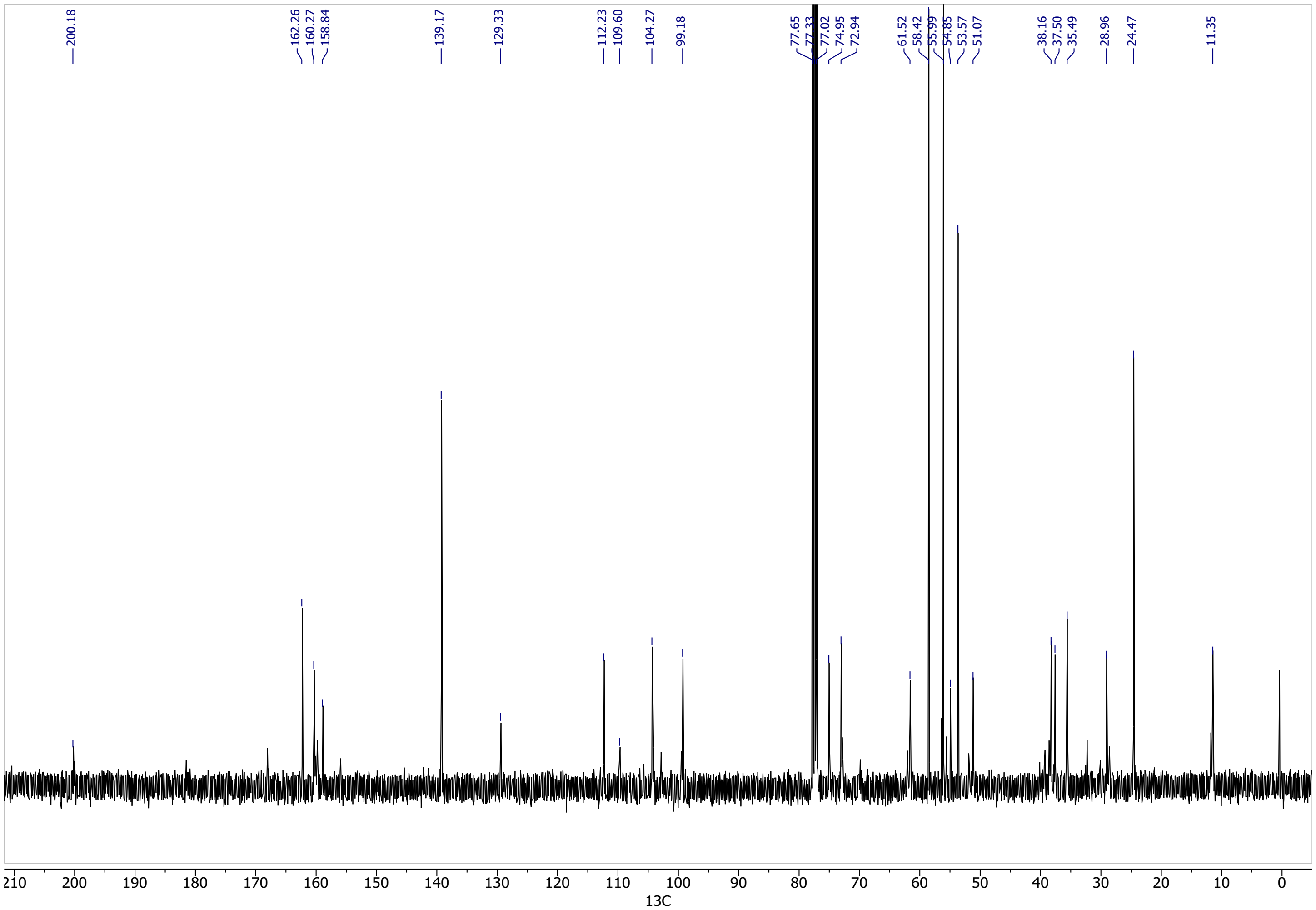

Supplemental Figure 19. ^{13}C NMR of Speciogynine pseudoindoxyl (SPG PI/10) (100 MHz, $CDCl_3$)

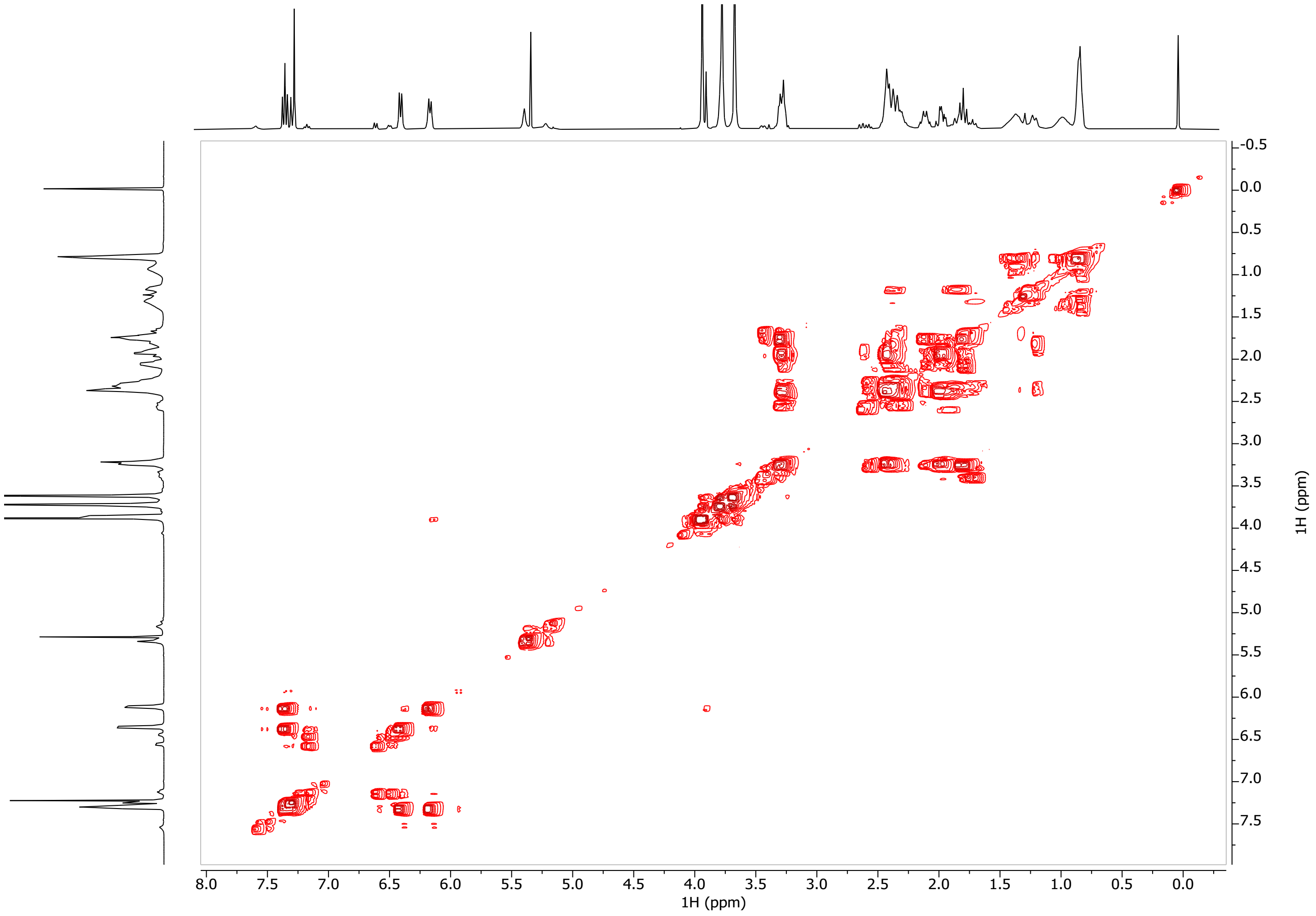

Supplemental Figure 20. COSY Spectrum of Speciogynine pseudoindoxyl (SPG PI/10) (400 MHz, $CDCl_3$)

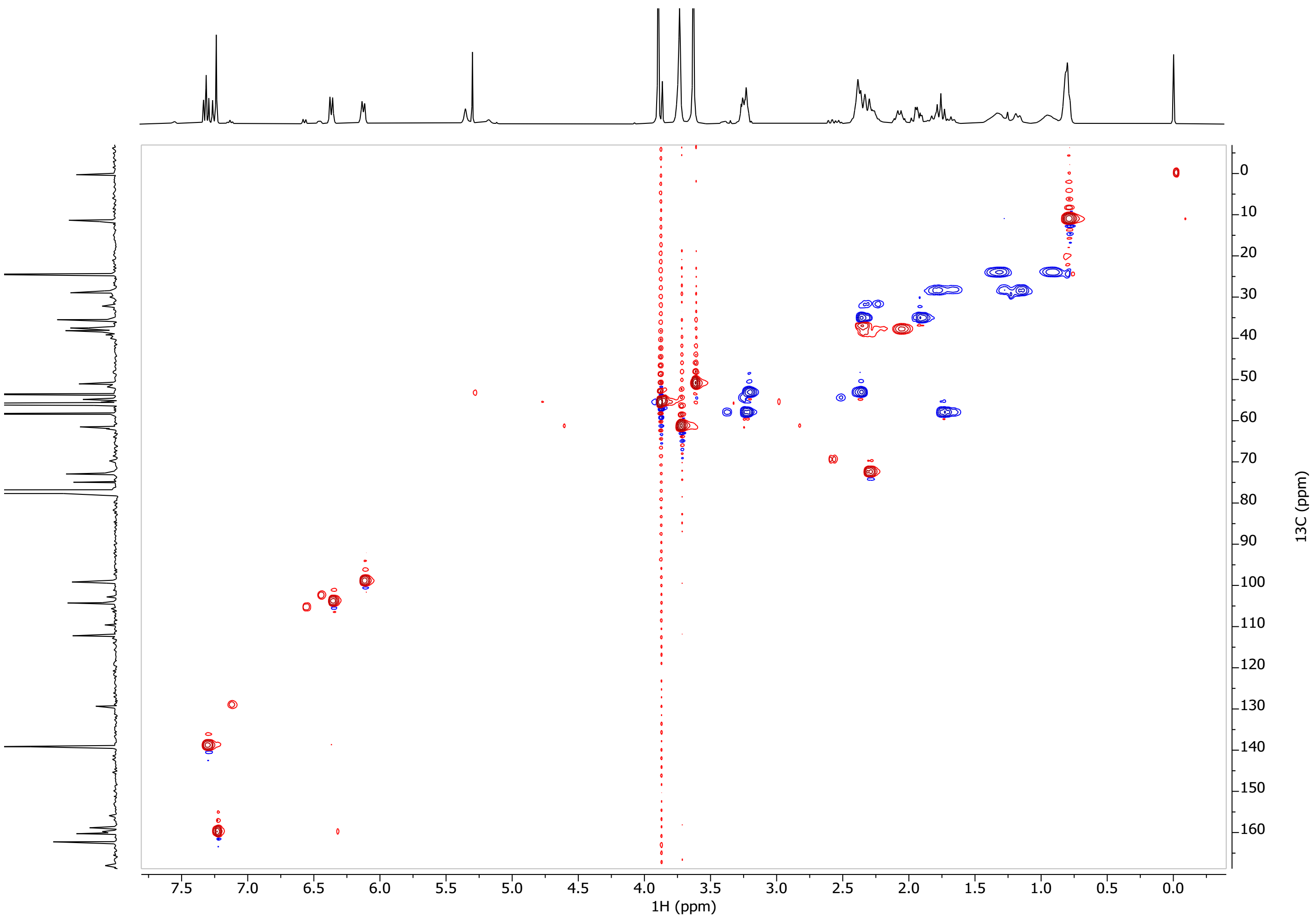

Supplemental Figure 21. HSQC Spectrum of Speciogynine pseudoindoxyl (SPG PI/10) (400 MHz, $CDCl_3$)

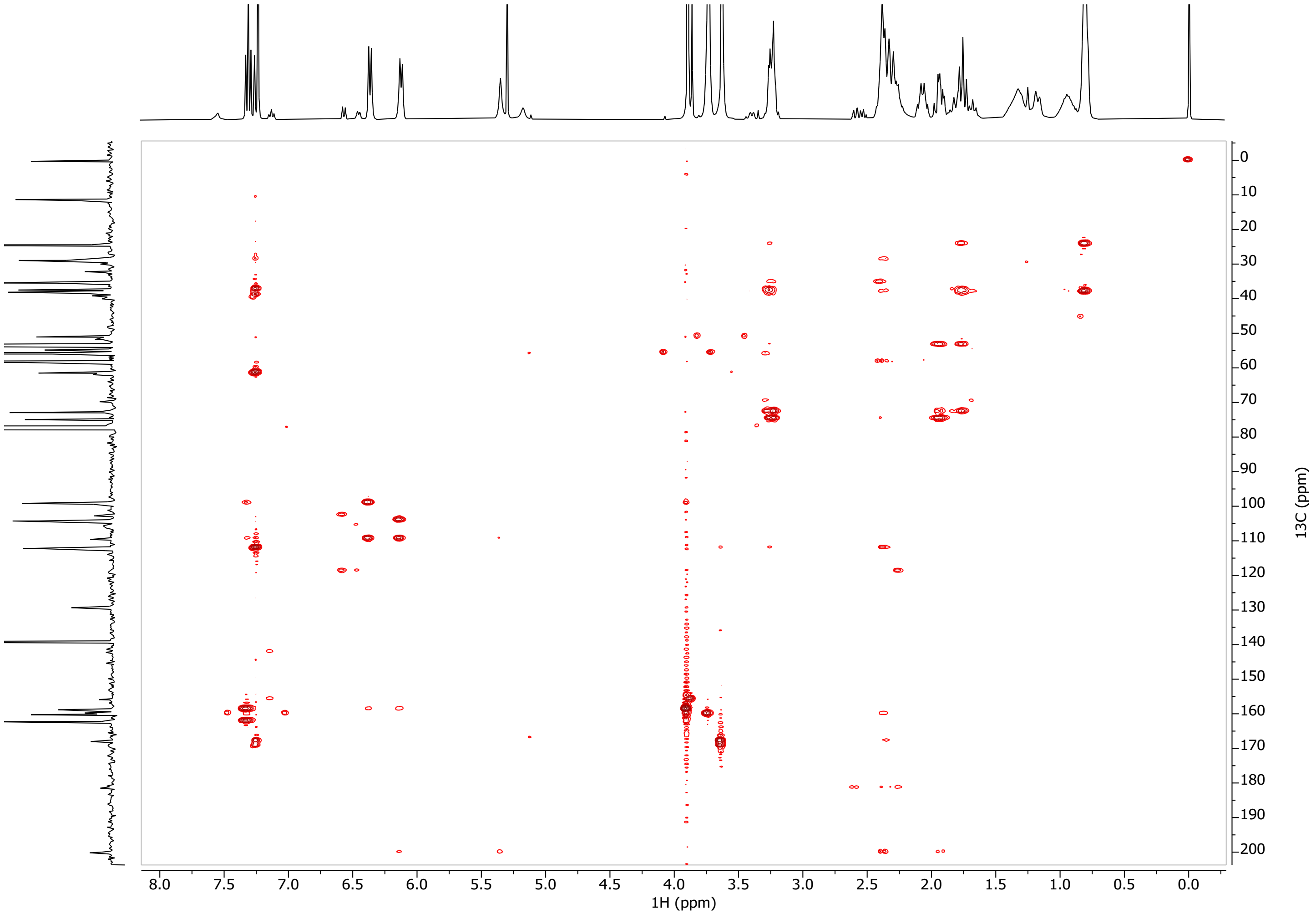

Supplemental Figure 22. HMBC Spectrum of Speciogynine pseudoindoxyl (SPG PI/10) (400 MHz, $CDCl_3$)

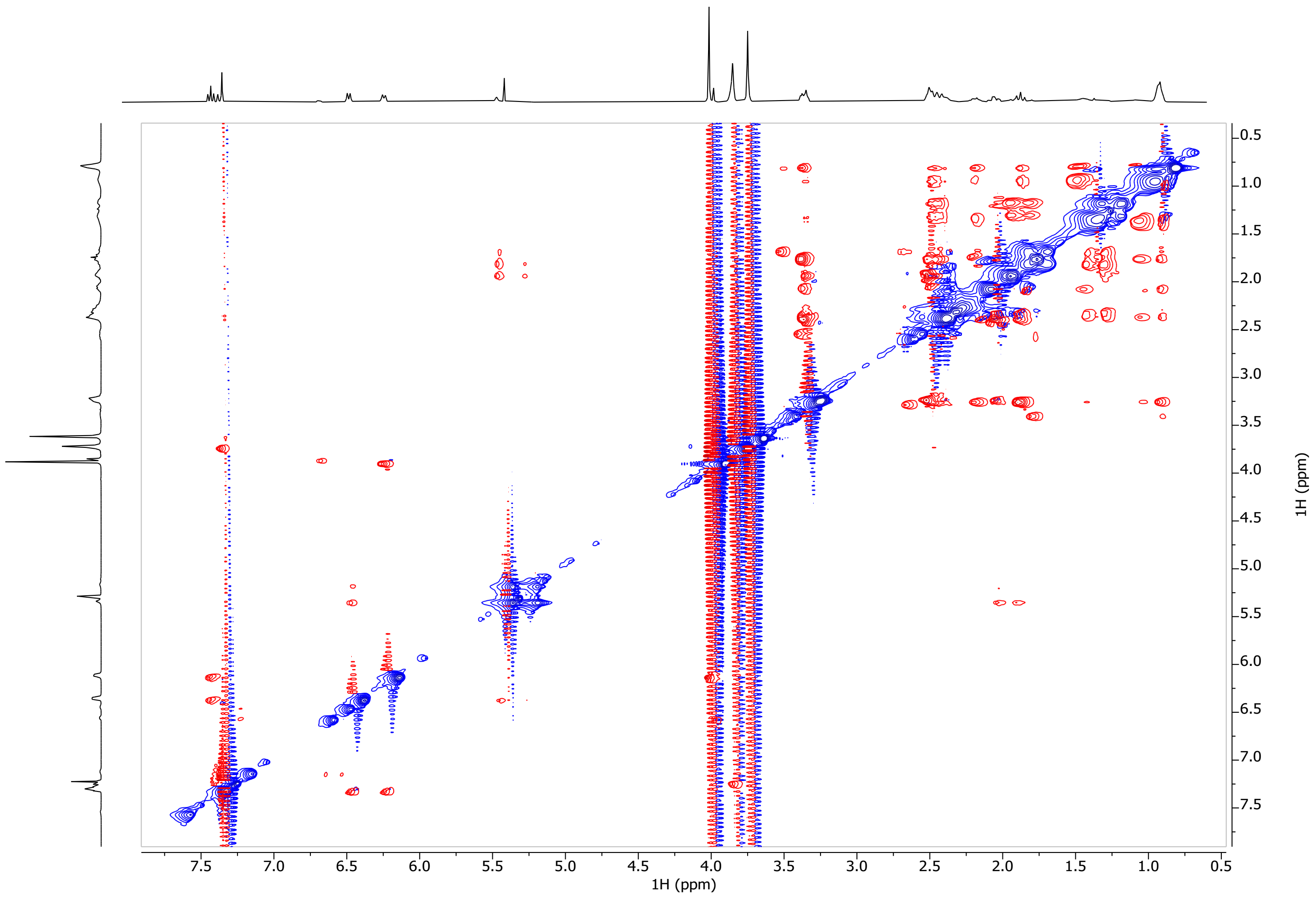

Supplemental Figure 23. NOESY Spectrum of Speciogynine pseudoindoxyl (SPG PI/10) (400 MHz, $CDCl_3$)
